# 3D Urban Outdoor WiFi 7 Network Planning and Analysis Using Ray-Tracing and Machine Learning: Transformer-Based Surrogate Modeling for High-Resolution Digital Twin †

**DOI:** 10.3390/s26072223

**Published:** 2026-04-03

**Authors:** Emanuel-Crăciun Trînc, Cosmin Ancuți, Andy Vesa, Călin Simu, Valentin-Adrian Niță, Cristina Stolojescu-Crişan

**Affiliations:** Department of Communications, Polytechnic University of Timisoara, 30006 Timisoara, Romaniaandy.vesa@upt.ro (A.V.);

**Keywords:** digital twin, wireless, ray-tracing, WiFi 7, machine learning, FT-Transformer

## Abstract

Accurate modeling of outdoor wireless propagation in dense urban environments is essential for smart city connectivity. Deterministic ray-tracing techniques provide high-fidelity multipath insight; however they suffer from high computational cost and limited scalability in large 3D environments. This work proposes a hybrid framework combining MATLAB-based (MATLAB 2024b 24.2.0.2773142, 64-bit, 22 October 2024) ray tracing and Machine Learning for scalable Wi-Fi 7 channel analysis. A large dataset is generated over a realistic university campus across multiple frequency bands, transmit powers, and reflection/diffraction configurations. Several regression models are evaluated, with emphasis on transformer-based architectures. The FT-Transformer achieves a Mean Absolute Error (MAE) of 3.49 dB, RMSE of 5.36 dB, and an $R^{2}$ of 99.63% for validation, reducing computation time from months of simulation to seconds at inference. The framework enables accurate and efficient surrogate modeling for network planning and digital twin applications.

## 1. Introduction

**Figure 1:**
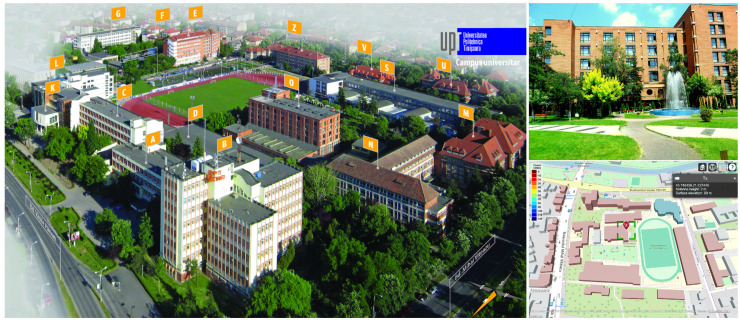
Real-world view of the Politehnica University Timișoara campus used as the study area, indicating the placement of the Wi-Fi access point (AP) on top of Building O (Mechatronics), which acts as the transmitter reference for the digital twin and ray-tracing analysis (image source https://arhiva.upt.ro/Informatii_contact-upt_122_ro.html, accessed on 23 March 2026).

Smart city infrastructures are becoming increasingly reliant on dense and reliable wireless connectivity to support public services, large-scale Internet of Things (IoT) deployments, campus networks, and outdoor urban applications. In such scenarios, wireless propagation is inherently three-dimensional and is significantly affected by the complex geometry and spatial layout of buildings, as observed in the real-world campus environment considered in this study (see Figure 1). In this context, Wi-Fi has evolved beyond its traditional indoor role to become a key access technology for urban-scale connectivity owing to its cost-efficiency, operation in the unlicensed spectrum, and continuous evolution towards higher throughput and lower latency [1,2]. Recent studies have emphasized that next-generation Wi-Fi systems are expected to complement cellular technologies in smart cities, enabling flexible, high-capacity access in outdoor and semi-outdoor environments such as campuses, pedestrian zones, and transport hubs [3,4].

Accurately predicting Wi-Fi coverage in urban environments is challenging because of their inherently three-dimensional (3D) nature. The presence of buildings of varying heights, irregular layouts, vegetation, and street canyons significantly influences radio propagation through reflection, diffraction, and shadowing effects. This results in strong spatial variability in signal strength and link quality. Therefore, simplified two-dimensional planning assumptions and empirical propagation models are often insufficient to capture the dominant physical mechanisms governing outdoor Wi-Fi performance in dense urban areas [5,6,7].

Deterministic ray-tracing techniques are widely regarded as one of the most physically accurate approaches for modeling wireless propagation in complex three-dimensional (3D) environments [5]. By explicitly simulating the interactions between radio waves and surrounding structures, ray tracing enables the detailed analysis of phenomena such as multipath propagation, frequency-dependent attenuation, and non-line-of-sight behavior [5,6]. The increasing availability of high-resolution geographic data, such as OpenStreetMap (OSM), has further facilitated the construction of large-scale, realistic 3D urban radio environments. These simulation-based representations closely align with the concept of a digital twin, whereby a virtual replica of the physical environment is employed to analyze, predict, and optimize system behavior [8,9].

Despite their high fidelity, ray-tracing simulations suffer from two fundamental limitations. First, the computational cost increases rapidly with environmental complexity, carrier frequency, and the number of permitted reflections and diffractions. This often requires weeks or months of computation on powerful multicore server infrastructures to exhaustively explore scenarios (see Appendix C) [5,10]. Second, it is typically infeasible to validate ray-tracing results on a large scale in real urban deployments, as acquiring comprehensive measurements across millions of transmitter–receiver combinations and diverse propagation conditions is prohibitively expensive and logistically impractical [6,7]. Consequently, ray tracing should not be considered as a direct substitute for measurements, but rather as a digital twin mechanism that allows the controlled exploration of propagation phenomena that cannot be directly observed on a large scale.

Although ray tracing-based digital twins provide valuable physical insights, there is a lack of comprehensive studies offering a statistical and system-level understanding of outdoor Wi-Fi propagation in realistic 3D urban environments. Existing Wi-Fi literature predominantly focuses on indoor scenarios or protocol-level optimizations, and comparatively few studies address outdoor propagation behavior across multiple frequency bands under realistic geometric conditions [1,5]. This gap is particularly relevant for modern Wi-Fi systems operating in higher frequency bands, where propagation is more sensitive to obstructions and multipath complexity.

Recent advances in Artificial Intelligence and Machine Learning provide a promising approach to overcome the computational limitations of ray tracing. By learning directly from large datasets generated with ray tracing, data-driven models can approximate complex propagation behavior at a computational cost that is orders of magnitude lower at inference time [1]. Transformer-based architectures, in particular, have demonstrated superior performance on structured and tabular data by modeling global feature dependencies through self-attention mechanisms [11,12]. In the field of wireless communication, attention-based models have shown great promise in channel modeling and radio map generation, facilitating the rapid and scalable prediction of spatial signal characteristics.

Inspired by these developments, this study proposes a Digital Twin Framework for smart cities oriented towards 3D outdoor Wi-Fi analysis. This framework combines large-scale ray-tracing simulations with Transformer-based Machine Learning models. Using a detailed OpenStreetMap (OSM)-derived 3D (geographic) model of the Politehnica University of Timișoara campus and the surrounding urban area, we generate an extensive dataset that captures simulated outdoor Wi-Fi propagation across multiple frequencies, transmission powers, and propagation complexities (see Appendix A). Furthermore, we demonstrate that Transformer-based models can accurately learn the underlying ray-tracing behavior. This enables the generation of detailed Wi-Fi propagation estimates in seconds, which would otherwise require weeks or months of simulation time on high-performance computing infrastructure, such as that presented in Appendix C.

The main contributions of this work can be summarized as follows:

- The design and implementation of a scalable, 3D, outdoor, wireless digital twin framework for campus-scale and smart city Wi-Fi environments, enabling the systematic exploration of propagation behavior across complex urban geometries.
- The generation of a large-scale, physics-consistent wireless propagation dataset comprising millions of transmitter–receiver interactions across multiple frequencies, transmission powers, and propagation configurations.
- The development and evaluation of a transformer-based surrogate modeling approach that accurately approximates deterministic ray-tracing outputs while reducing inference time from months of simulation to near-instantaneous prediction.
- The integration of physically grounded electromagnetic simulation with Artificial Intelligence-driven acceleration, establishing a hybrid modeling paradigm for scalable wireless network planning and analysis.
- The provision of a foundational dataset and methodological framework intended to support subsequent research in channel modeling, beamforming optimization, AI-assisted network planning, and educational applications in wireless communication engineering.

The remainder of this paper is organized as follows. Section 2 defines the research scope and motivates the dataset-driven digital twin approach. Section 3 reviews related work and positions the proposed framework within existing ray-tracing and machine learning-based channel modeling literature. Section 4 presents the theoretical foundation, including IEEE 802.11 evolution and the electromagnetic principles underlying deterministic ray tracing. Section 5 describes the materials and methods, detailing the MATLAB-based simulation environment, dataset generation process, and antenna configuration. Section 6 reports the experimental results and evaluates the performance of the proposed machine learning models. Finally, Section 7, Section 8 and Section 9 conclude the paper and outline directions for future research.

## 2. Research Scope and Dataset-Driven Digital Twin Motivation

The primary objective of this study is to establish a scalable and physically grounded dataset generation framework for outdoor wireless propagation modeling in complex urban environments. Rather than focusing exclusively on point-level validation against real-world measurements (see, for example, [13]), this work emphasizes the creation of large, physically consistent propagation datasets that can serve as a foundational platform for subsequent research and simulation-driven wireless system development.

Deterministic ray tracing is widely regarded as one of the most physically grounded and accurate approaches for modeling electromagnetic wave propagation in complex environments. For example, Ron Levie et al. [14] state that, “Given the fact that ray tracing commercial tools are routinely used for wireless networks layout planning, it is clear from engineering experience that high accuracy ray tracing can predict real-life measurements sufficiently well”. By explicitly simulating wave interactions with surrounding structures based on geometric optics principles and electromagnetic theory, ray-tracing methods are capable of capturing reflection, diffraction, and multipath effects with high spatial fidelity [5,15].

Extensive validation studies have demonstrated strong agreement between ray tracing predictions and real-world measurements when sufficient environmental detail is incorporated [15,16]. Owing to its explicit modeling of electromagnetic wave interactions with environmental structures based on geometric optics principles, deterministic ray tracing is widely regarded as one of the most physically grounded and realistic propagation modeling approaches available for large-scale wireless radiation analysis. By simulating reflection, diffraction, and shadowing mechanisms in complex three-dimensional environments, ray tracing provides a high-fidelity approximation of the real-world large-scale propagation behavior. Therefore, the present study adopts ray tracing as the foundational propagation model and builds upon this established framework to generate physics-consistent digital twin datasets. Rather than replacing the underlying propagation model, the proposed approach leverages ray-tracing outputs as a reliable baseline for scalable machine learning-driven surrogate modeling and wireless network planning analysis.

The dataset generated in this study is intended to support multiple research and industrial applications, including wireless coverage planning, radio channel modeling, beamforming optimization, and network deployment strategy evaluation. Furthermore, large-scale propagation datasets provide valuable training data for Machine Learning models designed to approximate complex electromagnetic interactions, thereby enabling the development of next-generation simulation platforms and intelligent wireless network planning tools.

Although real-world measurement validation remains an essential component of wireless propagation research, such campaigns typically require extensive logistical coordination, specialized measurement equipment, and controlled experimental environments [13]. Consequently, simulation-driven datasets represent an important intermediate step toward enabling scalable wireless system analysis and Machine Learning model development.

This work therefore positions ray-tracing simulation not as a replacement for measurement-driven validation, but as a complementary digital twin framework that enables systematic dataset generation. As simulation tools and environmental reconstruction technologies continue to evolve, future studies (see Section 9) may further refine propagation accuracy and integrate measurement-based calibration. In addition to research applications, the generated datasets also provide valuable educational resources for academic training in wireless communication and radio-frequency propagation analysis.

## 3. Related Work

### 3.1. Wi-Fi Evolution and Smart City Connectivity

The IEEE 802.11 family of standards has evolved continuously to address increasing demands for throughput, latency reduction, and spectral efficiency. Recent surveys and technical analyses describe the transition from Wi-Fi 6/6E to Wi-Fi 7 (IEEE 802.11be), highlighting features such as ultra-wide 320 MHz channels, 4096-QAM modulation, multi-link operation, and enhanced multi-user coordination [17,18]. These advancements position Wi-Fi 7 as a key enabler for high-capacity wireless access in dense environments, complementing cellular technologies in smart city deployments.

Beyond physical-layer improvements, several studies emphasize the growing role of Wi-Fi in smart cities and large-scale outdoor scenarios, including campuses, transportation hubs, and public spaces. Szott et al. [1] provide a comprehensive survey on the application of machine learning to IEEE 802.11 systems, noting that most existing work focuses on indoor environments and protocol optimization, with limited attention given to outdoor propagation and spatial coverage analysis. This observation motivates further investigation into realistic outdoor Wi-Fi behavior under complex urban geometries.

### 3.2. Digital Twin-Based Ray Tracing for Antenna Optimization vs. Structured Multi-Task Digital Twin Modeling

Recent research has increasingly explored the integration of digital twin environments with deterministic ray-tracing techniques for wireless network optimization. A representative example is the work of Yildiz [19], which investigates antenna orientation optimization within a digital twin framework using ray-tracing analysis. In that study, the digital twin is employed to evaluate how different antenna orientation configurations influence received power distributions in a given environment. The primary objective is performance optimization through simulation-driven antenna alignment, with ray tracing serving as the core physical modeling engine.

Although both the work of Yildiz [19] and the present study utilize deterministic ray tracing within a digital twin framework, the underlying modeling abstractions and research objectives differ fundamentally. The work in [19] focuses on configuration-level optimization within a specific deployment scenario, where the digital twin is used to evaluate orientation-dependent coverage patterns. In contrast, the present study constructs a large-scale, structured dataset derived from ray-tracing simulations, where each transmitter–receiver interaction is represented as a parametrized sample enriched with geometric, environmental, frequency-dependent, weather-dependent, and multipath interaction metadata.

Rather than optimizing a single configuration parameter (e.g., antenna orientation), the proposed framework aims to enable multi-target regression and classification tasks spanning signal prediction, channel complexity modeling, propagation regime identification, reliability assessment, and directional beam-alignment-aware decision modeling. This shifts the focus from scenario-specific optimization toward a generalized data-driven surrogate modeling layer that can support scalable network planning, multi-band analysis, and AI-assisted propagation intelligence.

Furthermore, whereas [19] employs ray tracing primarily as a deterministic evaluation tool, the present work leverages raw ray interaction statistics (e.g., reflection counts, diffraction events, interaction depth, and ray density) as explicit structured features for transformer-based learning. This enables modeling at the physical interaction level rather than solely at the configuration outcome level. As a result, the proposed digital twin framework establishes a foundation for extensible multi-objective learning tasks beyond antenna orientation optimization, including weather-aware reliability modeling and propagation morphology classification.

Therefore, the present study should be viewed as complementary to digital twin-based optimization works such as [19]. While prior research demonstrates the effectiveness of ray-tracing-driven configuration tuning within digital twin environments, this work extends the paradigm by constructing a physics-informed, structured dataset that supports scalable, multi-task AI modeling of wireless propagation phenomena.

In addition to configuration-level digital twin optimization, recent work has explored hybrid integration of deterministic ray tracing with advanced learning architectures for next-generation channel modeling. Li et al. [20] introduced DeepRT, a framework that combines large neural models with ray-tracing principles to enhance scalability and efficiency in 6G digital twin channel prediction. DeepRT focuses on accelerating channel modeling while preserving physical consistency derived from ray-tracing simulations, effectively bridging deterministic simulation and data-driven inference within emerging 6G environments.

While DeepRT advances hybrid channel modeling within digital twin systems, its primary emphasis remains on improving or accelerating channel prediction itself. In contrast, the present study operates at a different abstraction layer by transforming raw ray-tracing outputs into a structured, multi-feature dataset designed to support multi-task regression and classification modeling. Rather than modifying the ray-tracing engine or embedding it within a hybrid architecture, this work exposes physically meaningful interaction metadata as explicit learning features, enabling propagation regime classification, reliability-aware planning, weather-sensitive modeling, and beam-alignment-oriented decision tasks within a unified digital twin framework.

### 3.3. Image-Based Radio Map Learning vs. Structured Digital Twin Modeling

Deep learning approaches for radio environment map (REM) construction have recently gained considerable attention. A prominent example is RadioUNet [14], which formulates propagation prediction as an image-to-image classification task. In this paradigm, environmental layouts and transmitter locations are encoded as rasterized spatial grids, and a convolutional U-Net architecture predicts dense received power maps. Similarly, the dataset introduced in [21] provides large-scale pathloss and time-of-arrival (ToA) radio maps specifically designed to support supervised training of CNN-based spatial prediction models.

Convolutional architectures are well known to excel in image-domain problems due to their ability to exploit local spatial correlations through translation-equivariant filters and hierarchical feature extraction [22,23]. Consequently, CNN-based radio map predictors such as RadioUNet achieve strong performance when the propagation problem is expressed as dense spatial interpolation within fixed rasterized environments.

In contrast, the present work adopts a fundamentally different modeling abstraction. Rather than representing the propagation environment as a 2D image and predicting a coverage map directly, we construct a structured digital twin dataset derived from deterministic ray-tracing simulations. Each data sample corresponds to an individual transmitter–receiver interaction and includes detailed geometric, environmental, and system-level descriptors, as well as low-level multipath interaction statistics generated by the shooting-and-bouncing ray (SBR) engine. While the current study focuses on predicting a single target variable (RSSI), the dataset intrinsically captures richer channel information that enables future multi-target learning tasks, including rain attenuation modeling, multipath complexity estimation, propagation regime classification, and parametric network optimization.

This structured representation shifts the modeling focus from spatial interpolation to parametric propagation understanding. Unlike image-based REM datasets, which implicitly encode propagation physics within spatial pixel correlations, our dataset explicitly exposes physically meaningful features such as reflection counts, diffraction statistics, interaction depth, frequency variation, antenna configuration, and weather-dependent signal attenuation (heavy rain, 50 mm/m^2^). This provides a more direct bridge between deterministic electromagnetic simulation and data-driven modeling.

For heterogeneous tabular datasets of this nature, recent studies have demonstrated that transformer-based architectures outperform classical machine learning methods and convolutional networks in capturing complex feature interactions [11,12]. Self-attention mechanisms enable modeling of global dependencies across diverse propagation parameters without requiring rasterization of the environment. This makes transformer-based surrogate models particularly suitable for multi-frequency, multi-configuration digital twin simulations, where interactions between geometry, frequency, antenna height, weather, and multipath statistics are inherently non-local and parameter-dependent.

This structured digital twin perspective enables not only map reconstruction but also systematic exploration of wireless propagation mechanisms, opening avenues for interpretable, physics-aware AI models in next-generation network planning.

Therefore, the proposed framework should be viewed as complementary rather than competitive with CNN-based REM approaches such as RadioUNet. While image-domain models focus on reconstructing spatial coverage maps within predefined grids, the present work establishes a structured, physics-informed digital twin foundation for scalable wireless network planning, multi-target channel modeling, and AI-assisted propagation analysis. By leveraging raw ray-tracing interaction data instead of rasterized coverage images, this study extends the scope of data-driven wireless modeling toward richer parametric analysis and future multi-objective learning.

**Figure 2:**
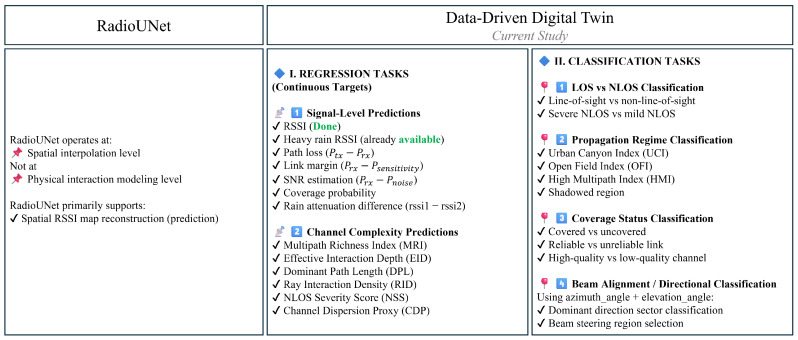
Conceptual comparison between image-based radio map learning (RadioUNet) and the structured data-driven digital twin framework proposed in this study. While RadioUNet operates primarily at the spatial interpolation level for RSSI map reconstruction, the structured digital twin approach enables multi-target regression, propagation regime classification, reliability assessment, and beam-alignment-aware directional modeling based on explicit ray-tracing interaction metadata.

This conceptual distinction between image-based spatial interpolation and structured digital twin modeling is summarized in Figure 2, which highlights the broader regression and classification capabilities enabled by the proposed framework. More details can be found in Appendix E.

### 3.4. Ray-Tracing Techniques for Wireless Propagation Modeling

Deterministic ray tracing has long been recognized as one of the most accurate approaches for modeling wireless propagation in complex environments. Classical works by Fuschini et al. [5] and Yun and Iskander [6] provide detailed overviews of ray-tracing principles, acceleration techniques, and practical applications in indoor and small-cell scenarios. These studies demonstrate the ability of ray tracing to capture multipath effects, non-line-of-sight propagation, and frequency-dependent attenuation with high physical fidelity.

Ray tracing has also been applied to more advanced wireless systems, including coordinated multi-point transmission [7], reconfigurable intelligent surfaces, and satellite-to-ground communications [24]. Liu et al. [25] further extended three-dimensional ray-tracing-based propagation modeling to macrocellular environments at sub-6 GHz frequencies. Their work emphasizes detailed 3D environmental reconstruction and calibrated electromagnetic modeling to improve large-scale propagation prediction accuracy in realistic outdoor deployments. Such studies highlight the continued relevance of deterministic ray tracing as a physically grounded tool for modeling next-generation wireless systems.

More recently, simulation platforms such as WiThRay [10] have been proposed to support flexible ray-tracing-based channel modeling in smart wireless environments. Despite these advances, the computational complexity of ray tracing remains a fundamental limitation, particularly in large-scale 3D urban environments and at higher frequencies. As a result, exhaustive ray-tracing simulations are generally confined to offline studies and a limited subset of the full parameter space, since exploring all combinations of frequencies, transmitter locations, and propagation conditions is computationally prohibitive.

### 3.5. Validation of Ray-Tracing Simulations Using Real-World Measurements

Although deterministic ray-tracing techniques provide high-fidelity modeling of wireless propagation, their reliability must be assessed through comparison with real-world measurements. A representative validation study by Militaru et al. [13] compared MATLAB-based ray-tracing simulations with empirical 5G measurements collected in a dense urban environment using commercial user equipment operating in the n78 band. The measurements included key channel indicators such as RSRP, SINR, and RSRQ along predefined pedestrian routes with automatic base-station handover.

The results demonstrated strong spatial correlation between simulated and measured signal patterns, with ray tracing accurately reproducing variability caused by building shadowing, reflections, and diffractions. However, point-level discrepancies were observed and attributed to simplified environmental modeling, limited knowledge of precise material properties, and the absence of stochastic effects such as small-scale fading and dynamic objects. Increasing propagation realism, including additional reflections and diffractions, was shown to improve agreement, highlighting the importance of higher-order multipath modeling.

These findings support the methodology adopted in this work. Rather than replacing measurements, ray tracing is employed as a physics-grounded digital twin generator for systematic dataset creation. While environmental simplifications—such as homogeneous materials, simplified building geometries, and omission of vegetation or dynamic elements—may introduce localized errors, deterministic ray tracing remains widely validated for coverage estimation and network planning [5,6]. Consequently, it provides a suitable foundation for training scalable machine learning surrogate models.

### 3.6. Distinction from Radio Environment Map (REM) Reconstruction Approaches

Recent work on Radio Environment Maps (REMs) has primarily focused on reconstructing spatial signal distributions from sparse or distributed measurements using statistical learning and interpolation techniques. For example, Gao et al. [26] investigate time-variant radio map reconstruction in dynamic spectrum environments using optimized distributed sensors, while Wang et al. [27] propose a hierarchical sparse Bayesian learning framework for 3D REM construction incorporating channel shadowing effects. These approaches emphasize measurement-driven map reconstruction and spatial interpolation under uncertainty. In contrast, the present study does not aim to reconstruct radio maps from sensor observations, but rather to generate a large-scale, physics-consistent propagation dataset using deterministic ray tracing and subsequently learn a surrogate model that approximates the underlying electromagnetic interactions. Instead of spatial interpolation, our framework operates at the transmitter–receiver interaction level, explicitly incorporating geometric, frequency-dependent, and multipath features derived from a structured digital twin environment. Therefore, while REM methodologies focus on data-driven reconstruction of observed coverage maps, our approach integrates physics-based simulation with transformer-based regression to enable scalable, configuration-aware wireless network planning.

### 3.7. Transformer Usage for Tabular Datasets

Transformer architectures specifically designed for structured and tabular data have shown strong performance in modeling heterogeneous feature interactions. The TabTransformer [11] and FT-Transformer [12] leverage self-attention mechanisms to capture global dependencies across diverse input variables, making them well suited for structured ray-tracing datasets where propagation characteristics depend jointly on geometry, frequency, antenna configuration, and environmental conditions. These developments motivate the adoption of transformer-based surrogate modeling within the structured digital twin framework proposed in this study.

## 4. Theoretical Foundation

### 4.1. IEEE 802.11 Standard Evolution

**Figure 3:**
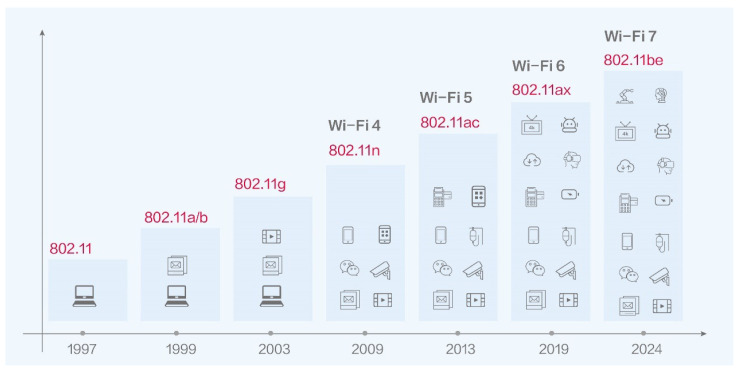
Timeline of IEEE 802.11 standard evolution from 1997 to 2024, highlighting key technical milestones and application trends from 802.11 (Wi-Fi 1) to 802.11be (Wi-Fi 7). Source: Ruijie Networks.

The IEEE 802.11 standard, commonly known as Wi-Fi, has undergone significant evolution since its inception in 1997. Initially designed for basic wireless LAN communication, each new generation has improved spectral efficiency, throughput, latency, and support for modern applications. Figure 3 summarizes the major milestones in Wi-Fi evolution.

- 802.11/Wi-Fi 1 (1997): The original Wi-Fi standard, operating in the 2.4 GHz band, with speeds up to 2 Mbps.
- 802.11a/b/Wi-Fi 2 (1999): Introduced higher speeds (up to 54 Mbps) using OFDM in the 5 GHz band (802.11a), and a more cost-effective 11 Mbps 2.4 GHz option (802.11b).
- 802.11g/Wi-Fi 3 (2003): Combined the speed of 802.11a with the range and compatibility of 802.11b in the 2.4 GHz band.
- 802.11n/Wi-Fi 4 (2009): Added MIMO (multiple input, multiple output) antennas, channel bonding, and dual-band support for higher throughput and reliability.
- 802.11ac/Wi-Fi 5 (2013): Enhanced MIMO and introduced MU-MIMO and wider channels (up to 160 MHz), offering Gbps-level throughput.
- 802.11ax/Wi-Fi 6 (2019): Focused on high-density environments with OFDMA, uplink MU-MIMO, and target wake time (TWT) for better efficiency and power saving.
- 802.11be/Wi-Fi 7 (2024): Aims to deliver extremely high throughput (EHT) using 320 MHz channels, 4096-QAM modulation, and Multi-Link Operation (MLO), supporting demanding applications such as AR/VR, 8K streaming, and real-time cloud gaming.

The evolution of IEEE 802.11 standards reflects a growing need for higher bandwidth, lower latency, and better spectrum utilization, especially in high-density urban and indoor environments. Wi-Fi 6 and Wi-Fi 7 play a crucial role in enabling next-generation applications like industrial automation, wireless AI inference, and massive IoT deployments.

#### 4.1.1. 2.4 GHz Wi-Fi Channels

The 2.4 GHz band represents the earliest and most widely deployed spectrum for Wi-Fi communications. In most regulatory domains, this band spans approximately 83.5 MHz, from 2.400 GHz to 2.4835 GHz, and is divided into 14 partially overlapping channels with center frequencies spaced 5 MHz apart. Despite the apparent availability of multiple channels, the effective usable spectrum is limited by the bandwidth requirements of Wi-Fi transmissions.

Traditional IEEE 802.11b systems employed direct-sequence spread spectrum (DSSS) with a channel bandwidth of approximately 22 MHz, while later standards such as IEEE 802.11g and IEEE 802.11n adopted orthogonal frequency-division multiplexing (OFDM) with an effective bandwidth of 20 MHz. As a consequence, adjacent channels in the 2.4 GHz band overlap significantly, leading to both adjacent-channel interference (ACI) and co-channel interference (CCI) when multiple access points operate in close proximity.

**Figure 4:**
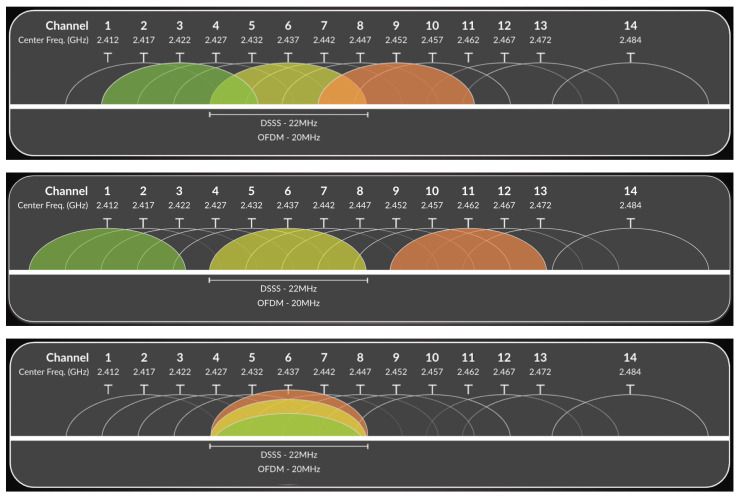
Channel allocation and interference mechanisms in the 2.4 GHz Wi-Fi band for 20–22 MHz channels. From top to bottom, the figure illustrates adjacent-channel interference, extensive channel overlap caused by 5 MHz channel spacing, and co-channel interference when multiple access points operate on the same channel. Due to the limited available spectrum, only a small number of non-overlapping channels can be used simultaneously, which significantly constrains capacity and spatial reuse in dense deployments. Images adapted from Ekahau.

Figure 4 illustrates the channel structure of the 2.4 GHz band and highlights the overlap between neighboring channels. Due to this overlap, only a limited set of non-overlapping channels, typically channels 1, 6, and 11 in most regions, can be deployed simultaneously without causing severe interference. While co-channel interference can be partially mitigated through carrier sense multiple access with collision avoidance (CSMA/CA), adjacent-channel interference is particularly detrimental, as overlapping transmissions are not coordinated by the medium access protocol.

The high susceptibility of the 2.4 GHz band to interference significantly constrains network capacity and spatial reuse in dense deployments, especially in outdoor and campus-scale environments. As a result, performance in the 2.4 GHz band is often limited not by signal strength alone, but by interference dynamics arising from channel overlap and shared medium access.

#### 4.1.2. 5 GHz Wi-Fi Channels

**Figure 5:**
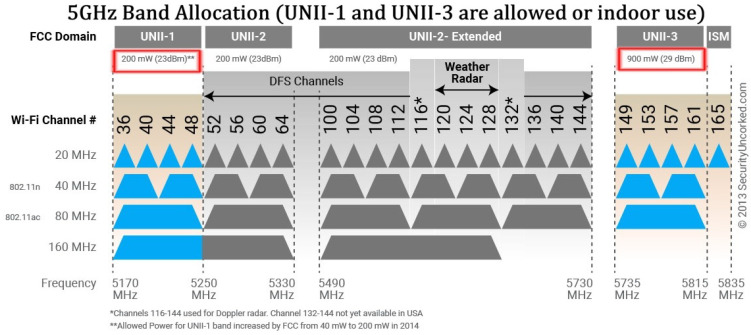
5 GHz Wi-Fi band allocation in the FCC regulatory domain, illustrating UNII-1, UNII-2, UNII-2 Extended, and UNII-3 sub-bands. The figure highlights available 20/40/80/160 MHz channel configurations, Dynamic Frequency Selection (DFS) requirements in radar-protected bands, and maximum permitted transmit power levels (e.g., 200 mW in UNII-1 and up to 900 mW in UNII-3). Image adapted from wifiadviser.com.

The expansion of Wi-Fi into the 5 GHz band significantly increased the amount of available spectrum compared to the 2.4 GHz ISM band, enabling a larger number of channels and wider channel bandwidths. Figure 5 provides an overview of the 5 GHz Wi-Fi spectrum, illustrating channel allocation across the Unlicensed National Information Infrastructure (U-NII) sub-bands, including U-NII-1, U-NII-2, U-NII-2e, and U-NII-3, as well as the presence of Dynamic Frequency Selection (DFS)-regulated frequencies.

As shown in Figure 5, the 5 GHz band supports a substantially higher number of non-overlapping channels for 20 MHz operation, which improves spatial reuse and reduces interference in dense deployments. Building on this increased spectral availability, channel bonding mechanisms introduced in IEEE 802.11n and extended in IEEE 802.11ac allow multiple adjacent channels to be combined, enabling bandwidths of 40 MHz, 80 MHz, and, in some configurations, 160 MHz.

However, the use of wider channels in the 5 GHz band introduces additional regulatory and operational constraints. A significant portion of the spectrum is subject to DFS requirements, which are intended to protect incumbent radar systems. Access points operating on DFS channels must perform channel availability checks and may be required to vacate channels upon radar detection, introducing delays and potential channel instability. As a result, despite the increased bandwidth compared to the 2.4 GHz band, effective channel availability in the 5 GHz band can be reduced in practice, particularly in outdoor and campus-scale deployments.

Overall, the 5 GHz band represents an important intermediate step in Wi-Fi evolution, alleviating many of the interference limitations of 2.4 GHz deployments while introducing new planning challenges related to regulatory constraints and channel bonding.

#### 4.1.3. 6 GHz Wi-Fi Channels

**Figure 6:**
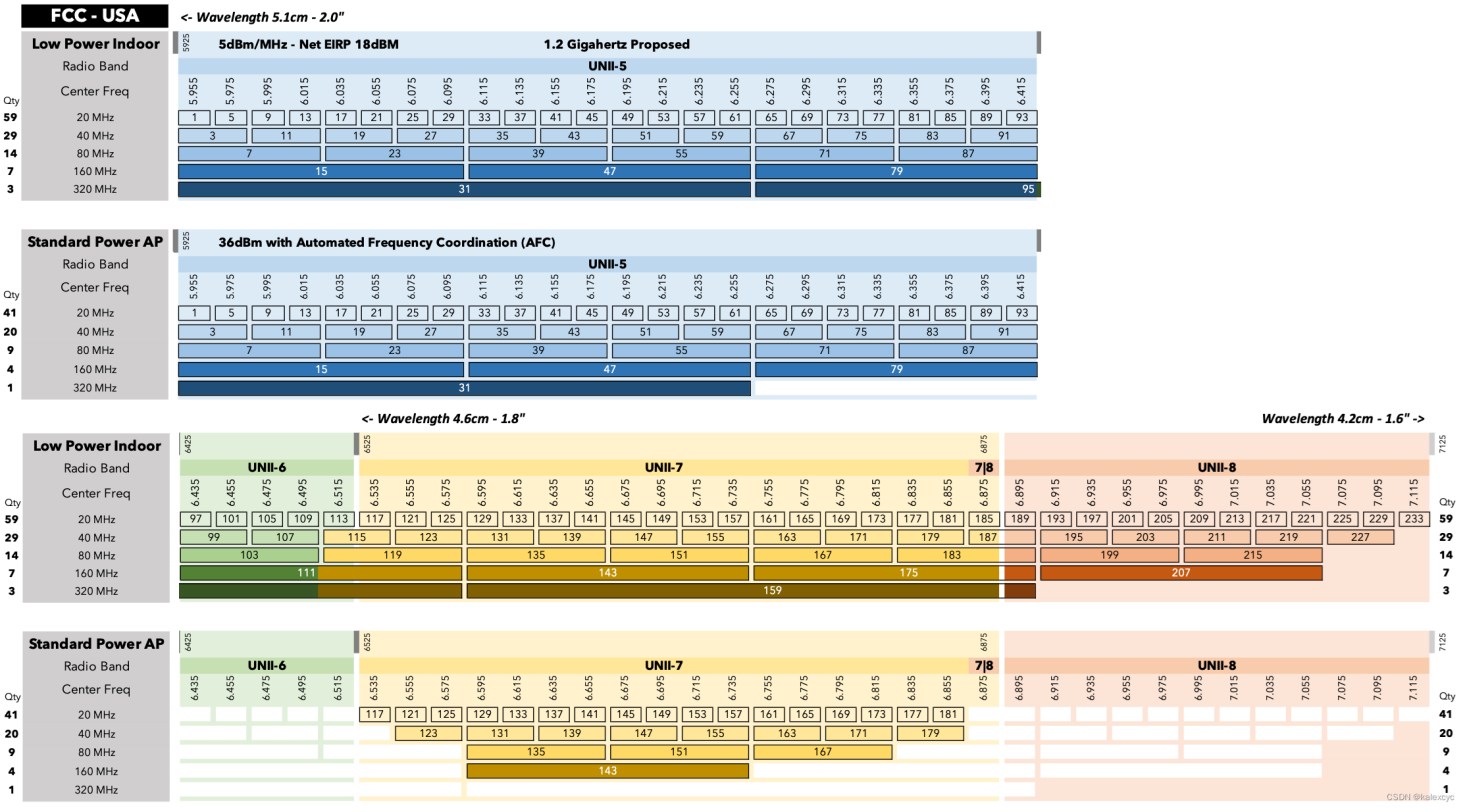
Channel allocation of the 6 GHz Wi-Fi band illustrating 20, 40, 80, 160, and 320 MHz channel groupings across the U-NII-5, U-NII-6, U-NII-7, and U-NII-8 sub-bands. The figure highlights actual channel numbers, center frequencies, and regulatory distinctions between low-power indoor (LPI) and standard-power access point operation with automated frequency coordination (AFC). The expanded contiguous spectrum enables ultra-wide 320 MHz channels introduced with IEEE 802.11be (Wi-Fi 7), supporting extremely high-throughput outdoor and campus-scale deployments. Image adapted from https://www.linkedin.com/ (accessed on 20 December 2025).

The opening of the 6 GHz band represents a major milestone in the evolution of Wi-Fi, providing a substantially larger and less congested spectrum compared to the legacy 2.4 GHz and 5 GHz bands. This spectrum expansion, introduced with Wi-Fi 6E and further extended in Wi-Fi 7, enables a dense and flexible channelization structure supporting both conventional and ultra-wide bandwidths. Figure 6 illustrates the channel allocation of the 6 GHz band, including actual channel numbers, center frequencies, and permissible channel widths across the U-NII-5, U-NII-6, U-NII-7, and U-NII-8 sub-bands.

As shown in Figure 6, the 6 GHz band supports a significantly larger number of non-overlapping 20 MHz channels, which can be aggregated to form wider channels of 40, 80, and 160 MHz without the extensive overlap constraints observed in lower-frequency bands. Most notably, the wide contiguous spectrum enables the use of 320 MHz channels, introduced with IEEE 802.11be (Wi-Fi 7), effectively doubling the maximum channel width supported by previous Wi-Fi generations.

The availability of such ultra-wide channels comes with important regulatory considerations. Depending on the region and deployment scenario, operation in the 6 GHz band may be restricted to low-power indoor (LPI) devices or may require automated frequency coordination (AFC) for standard-power access points. These constraints influence both channel availability and effective coverage, particularly in outdoor and campus-scale environments.

From a propagation perspective, the use of wider channels in the 6 GHz band shifts performance limitations away from spectral congestion toward environmental factors such as path loss, blockage, and multipath dispersion. While 320 MHz channels enable extremely high peak data rates, their practical performance is highly sensitive to three-dimensional urban geometry, reinforcing the need for accurate propagation modeling and data-driven digital twin approaches when analyzing next-generation Wi-Fi deployments.

### 4.2. Wireless Ray-Tracing

In wireless communication systems, the received power in a Line-of-Sight (LoS) scenario can be determined using the Friis transmission equation. This model provides a quantitative relationship between the transmitted power ($P_{t}$) and the received power ($P_{\mathrm{LoS}}$), considering the impact of the propagation environment and antenna characteristics. The received power is given in Equation (1).

(1) $$P_{\mathrm{LoS}}=P_{t}\cdot {\left(\frac{\lambda }{4\pi d_{\mathrm{LoS}}}\right)}^{2}\cdot G_{t}\cdot G_{r}$$

where $P_{t}$ is the transmitted power, λ is the signal wavelength, $d_{\mathrm{LoS}}$ is the transmitter–receiver separation, and $G_{t}$ and $G_{r}$ denote the transmitter and receiver antenna gains, respectively. The Friis model assumes free-space propagation with an unobstructed LoS path and therefore serves as a baseline for more complex propagation models.

In realistic outdoor and urban environments, however, wireless propagation is strongly influenced by interactions between electromagnetic waves and surrounding objects such as buildings, terrain, and vegetation. Ray-tracing techniques extend the LoS model by explicitly accounting for multipath propagation mechanisms, including specular reflections, diffractions around edges, and, in some cases, scattering from rough surfaces. Each propagation path is modeled as a ray that undergoes successive interactions with the environment before reaching the receiver.

For reflected paths, the received power contribution of the *i*-th ray can be expressed as given in Equation (2).

(2) $$P_{i}=P_{t}\cdot G_{t}\cdot G_{r}\cdot {\left(\frac{\lambda }{4\pi d_{i}}\right)}^{2}\cdot \prod_{k=1}^{N_{i}}{\left|\Gamma_{k}\right|}^{2}$$

where $d_{i}$ is the total propagation distance of the ray, $N_{i}$ is the number of reflections encountered, and $\Gamma_{k}$ denotes the Fresnel reflection coefficient at the *k*-th interaction. The reflection coefficient depends on the angle of incidence, polarization, and electromagnetic properties of the reflecting surface, such as relative permittivity and conductivity (see Appendix D for more details).

Diffraction effects become significant when the direct path is partially or fully obstructed. In ray-tracing models, diffraction is commonly approximated using knife-edge or uniform theory of diffraction (UTD) formulations, which introduce an additional diffraction loss term. For a diffracted ray, the received power can be modeled in Equation (3).

(3) $$P_{i}=P_{t}\cdot G_{t}\cdot G_{r}\cdot {\left(\frac{\lambda }{4\pi d_{i}}\right)}^{2}\cdot {\left|D\right|}^{2}$$

where *D* is the diffraction coefficient, which captures the attenuation caused by wave bending around obstacles and depends on the geometry of the diffracting edge and the wavelength.

The total received power at a given receiver location is obtained by coherently or incoherently summing the contributions of all valid rays, including the LoS component (if present), reflected rays, and diffracted rays. In practice, many ray-tracing implementations compute the received power as given by Equation (4).

(4) $$P_{\mathrm{RX}}=\sum_{i=1}^{N_{\mathrm{rays}}}P_{i}$$

where $N_{\mathrm{rays}}$ denotes the number of propagation paths considered, limited by parameters such as the maximum number of reflections and diffractions allowed in the simulation.

By explicitly modeling these propagation mechanisms, ray tracing provides a physically grounded representation of wireless channels in complex three-dimensional environments. However, the computational complexity of evaluating a large number of rays and interactions grows rapidly with environmental detail and frequency, motivating the use of data-driven surrogate models to approximate ray-tracing outputs in large-scale digital twin simulations.

### 4.3. MATLAB Ray-Tracing Support

MATLAB provides built-in support for deterministic wireless ray-tracing through its RF Propagation and Antenna Toolboxes, enabling physically grounded modeling of radio propagation in complex three-dimensional environments. Based on geometrical optics (GO) and uniform theory of diffraction (UTD) principles, the ray-tracing framework explicitly models line-of-sight (LoS), reflected, and diffracted propagation paths between a transmitter and multiple receiver locations.

**Figure 7:**
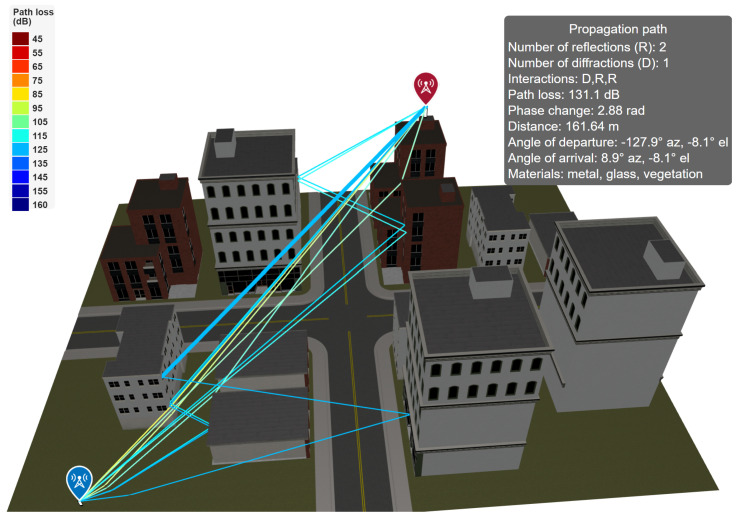
Example of deterministic ray-tracing visualization in MATLAB showing multiple propagation paths between a transmitter and a receiver in a three-dimensional urban environment. Each ray is characterized by its number of reflections and diffractions, interaction materials, path length, phase change, and angular properties. Color coding indicates path loss (dB), highlighting the attenuation differences between LoS, reflected, and diffracted paths (generated from Mathworks Inc. examples).

Figure 7 illustrates a representative ray-tracing scenario in an urban environment, where multiple propagation paths are generated between a transmitter and a receiver by accounting for reflections from building facades and diffraction around edges. Each ray corresponds to a distinct propagation path characterized by its total path length, number of reflections, number of diffractions, interaction materials, and geometric parameters such as angles of departure and arrival.

For each valid propagation path, MATLAB computes the associated path loss by combining free-space attenuation with interaction-specific losses introduced by reflections and diffractions. Reflections are modeled using Fresnel reflection coefficients derived from the electromagnetic properties of the interacting surfaces, such as relative permittivity and conductivity, while diffraction losses are calculated using UTD-based formulations. Material properties (e.g., concrete, glass, metal, vegetation) can be explicitly assigned to scene objects, allowing the ray-tracing model to capture environment-dependent attenuation effects.

In addition to path loss, MATLAB provides access to detailed per-ray metadata, including phase shifts, angles of arrival and departure, and interaction points. This information enables coherent or incoherent summation of multipath components at the receiver, as well as fine-grained analysis of multipath structure and angular dispersion. Simulation complexity is controlled through user-defined parameters such as the maximum number of reflections and diffractions considered, allowing a trade-off between physical accuracy and computational cost.

While MATLAB’s ray-tracing framework enables high-fidelity modeling of wireless propagation in realistic environments, the computational burden increases rapidly with scene complexity, frequency, and the number of allowed interactions. This limitation motivates the use of data-driven surrogate models capable of learning ray-tracing behavior from simulation data, enabling fast propagation prediction within large-scale digital twin environments.

### 4.4. Significance for Wi-Fi 7 Channel Prediction

The continuous evolution of IEEE 802.11 standards, culminating in the emergence of Wi-Fi 7 (802.11be), introduces unprecedented complexity in wireless channel behavior. With support for ultra-wide 320 MHz bandwidths, 4096-QAM modulation, multi-link operation (MLO), and low-latency scheduling, Wi-Fi 7 deployments demand highly accurate, real-time channel knowledge.

Traditional empirical or simplified propagation models may struggle to reflect the dynamic and high-resolution behavior of Wi-Fi 7 channels, especially in indoor or dense urban environments. Ray-tracing provides a physically accurate foundation for modeling such behavior, but remains computationally expensive and configuration-dependent.

To bridge this gap, our proposed method leverages supervised machine learning on ray-tracing-generated datasets to learn patterns in signal strength, path complexity, and interaction features. By doing so, we aim to develop a scalable and portable channel predictor that captures the frequency-, spatial-, and power-specific characteristics of Wi-Fi 7 environments without relying solely on repeated full-resolution simulations.

Thus, understanding the trajectory of IEEE 802.11 evolution is not just historical context, it is foundational for justifying the need for intelligent, data-driven wireless modeling in next-generation networks.

## 5. Materials and Methods

### 5.1. MATLAB Coverage Map Environment Setup

The MATLAB-based coverage map simulations were executed using MATLAB R2024a with the RF Toolbox, Antenna Toolbox, and Mapping Toolbox. This computational environment enables the visualization and analysis of radio coverage over geospatial data imported from OpenStreetMap (OSM), ensuring a reproducible and physically accurate framework for modeling Wi-Fi propagation within the Timișoara, Romania urban area.

The simulation workflow begins with the import of six OSM layers:

- timisoara_center.osm
- timisoara_north.osm
- timisoara_east.osm
- timisoara_south.osm
- timisoara_west.osm
- upt_campus.osm

These OSM layers represent the city’s central and peripheral districts, as well as the Politehnica University campus. Each dataset was read using MATLAB’s readgeotable() function with the buildingparts layer enabled. All individual maps were concatenated into a unified geotable, resulting in a 3D city model containing approximately 31,404 buildings. Material information (e.g., brick, concrete, metal, glass) was mapped to specific colors through a MATLAB dictionary to facilitate visual differentiation within the siteviewer. Loading and merging the complete geographic dataset (3D buildings) required approximately 20 min on the powerful simulation servers, due to the geometric complexity of the urban model.

A single transmitter (txsite) was placed at the Politehnica University Timișoara Campus location (45.746458° N, 21.227445° E), placed on top of Building O (Figure 1) to represent a local Wi-Fi Access Point (AP) or IoT gateway. The transmitter operated at a frequency of 2.4, 5, and 6 GHz with a power level of 100, 200, 500, or 1000 mW, and used a half-wave dipole antenna designed via design(dipole, f), mounted at a height of 5 m. The receiver sensitivity threshold was set to −90 dBm, corresponding to typical Wi-Fi device characteristics.

Signal propagation was modeled using the Shooting and Bouncing Rays (SBR) method through MATLAB’s propagationModel(“raytracing”) function. The model was configured to account for both reflections and diffractions, defined respectively by the parameters MaxNumReflections and MaxNumDiffractions. Coverage was computed using the coverage() function with a 350 m maximum range and 5 m spatial resolution. The signal strength levels were displayed between −90 dBm and −40 dBm, using a semi-transparent overlay for improved visual integration with the urban geometry.

**Figure 8:**
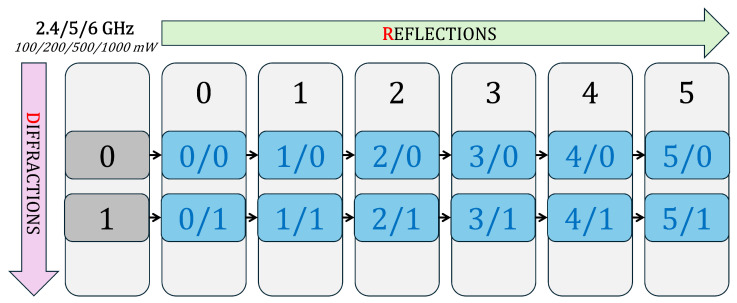
Reflection–diffraction configuration matrix used in the MATLAB coverage map simulation. Each R/D cell corresponds to a unique propagation model setup that can be repeated across frequencies (2.4, 5, and 6 GHz) and transmit power levels (100, 200, 500, and 1000 mW).

The configuration space for the simulations is illustrated in Figure 8, where each cell represents a reflection–diffraction (R/D) combination. The setup supports repetition of these simulations for all relevant frequency bands (2.4 GHz, 5 GHz, and 6 GHz) and transmitter power levels (100 mW, 200 mW, 500 mW, and 1000 mW), thereby enabling multi-scenario evaluation of propagation characteristics under varying power and frequency conditions.

The coverage environment was rendered using the OpenStreetMap basemap within MATLAB’s siteviewer, allowing interactive inspection of 3D structures and ray interactions. Execution timestamps were logged at both start and completion using datetime(‘now’) to ensure reproducibility and enable runtime benchmarking. This environment serves as the foundation for the subsequent receiver grid generation and RSSI regression modeling presented in Section 4.2 and Section 4.3.

### 5.2. 3D Coverage Map Visualization Setup

To accurately represent signal propagation in an urban environment, a three-dimensional (3D) coverage map was generated using MATLAB’s siteviewer environment. The visualization integrates topographical data, building geometries, and radio coverage results to enable interactive exploration of the simulation domain. The rendering was based on the OpenStreetMap (OSM) basemap, which provides detailed building footprints, street layouts, and vegetation features of the Politehnica University of Timișoara campus and its surrounding areas.

The imported OSM datasets include six geospatial layers covering the central, northern, eastern, southern, western, and campus regions of Timișoara. Each layer was preprocessed with the readgeotable() function, focusing on the buildingparts attributes to extract building height and material information. The combined dataset resulted in a city-scale model of 31,404 buildings, which were rendered as 3D extrusions within the viewer.

The transmitter (txsite) was placed at coordinates 45.746458° N and 21.227445° E, at a height of 5 m above the building level. The antenna configuration was defined using the MATLAB function design(dipole, f) for each operational frequency. The propagation model used the propagationModel(“raytracing”) function configured for Shooting and Bouncing Rays (SBR) analysis. This level of complexity provides a balance between physical accuracy and computational efficiency for dense urban environments.

Signal strength values were computed using the coverage() function with parameters:

- SignalStrengths: [-90:5:-40]
- MaxRange: 350 m
- Resolution: 5 m
- Transparency: 0.6

**Figure 9:**
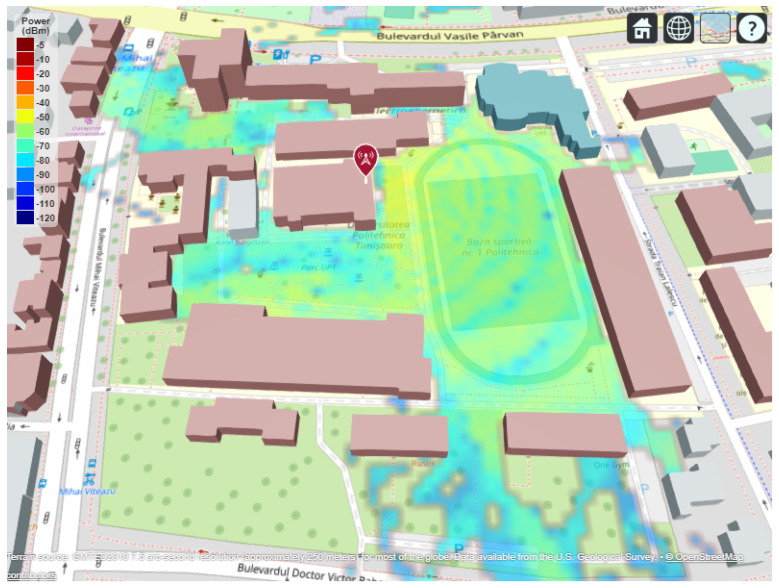
3D radio coverage map generated in MATLAB’s siteviewer environment using the ray-tracing model (MaxNumReflections = 1, MaxNumDiffractions = 1) at 2.4 GHz, 0.1 W transmit power. The transmitter is positioned on top of the Mechatronics (building O) of the Politehnica University of Timișoara campus. The color scale indicates received power (dBm).

The resulting power map (Figure 9) displays spatial variations in received signal strength across the area, where red areas correspond to strong signal coverage (−40 dBm to −60 dBm), and blue regions represent weak or obstructed reception (−90 dBm or lower). The visual output effectively correlates the influence of building density and material composition on signal attenuation and multipath effects.

This interactive 3D rendering allows for zooming, rotation, and ray inspection directly within the MATLAB environment, supporting deeper analysis of line-of-sight (LoS) and non-line-of-sight (NLoS) conditions. Consequently, the setup serves as a reliable testbed for validating propagation models under realistic urban conditions, which will be extended in subsequent sections to multi-frequency and multi-power scenarios.

### 5.3. Antenna Configuration and Theoretical Model

**Figure 10:**
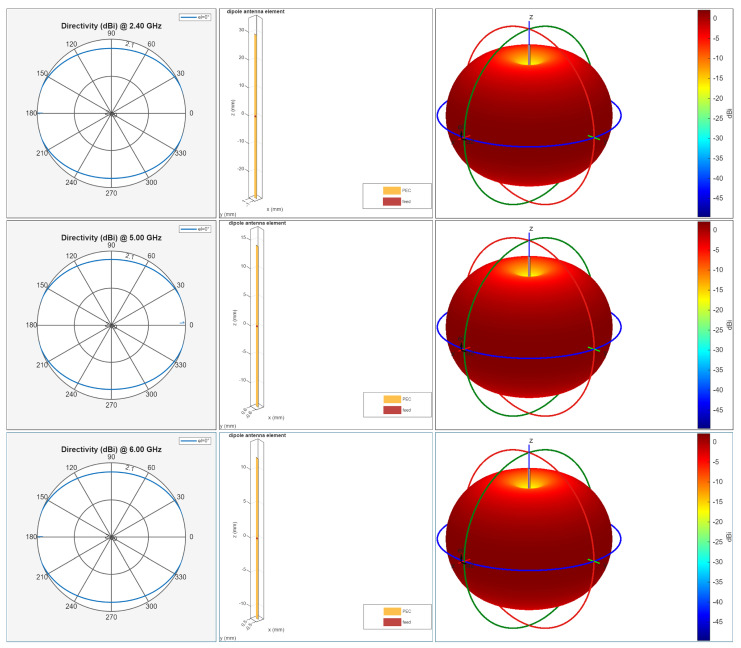
Half-wave dipole antenna models and radiation behavior at 2.4 GHz, 5.0 GHz, and 6.0 GHz. Left: azimuthal directivity cut (near-omnidirectional in the horizontal plane). Center: 3D dipole geometry generated by MATLAB (design(dipole,f)). Right: 3D directivity distribution showing the expected toroidal pattern with nulls along the dipole axis.

Figure 10 summarizes the transmitting antenna used throughout this study: a center-fed half-wave dipole designed in MATLAB for each operating band (2.4, 5.0, and 6.0 GHz) using design(dipole,f). The dipole was selected as a reference radiator because it provides a stable, well-understood omnidirectional azimuth response, making it suitable for isolating propagation effects (e.g., reflections/diffractions) from antenna-specific beamforming artifacts.

#### 5.3.1. Geometric Scaling with Frequency

For an ideal half-wave dipole, the total length *L* scales with the free-space wavelength given in Equation (5).

(5) $$L\approx \frac{\lambda }{2}=\frac{c}{2f}$$

where *c* is the speed of light and *f* is the carrier frequency. Accordingly, the dipole geometry becomes shorter at higher frequencies (Figure 10, center), while maintaining the same electrical length and resonant behavior. In our simulations, MATLAB automatically adjusts the dipole dimensions to satisfy the half-wave resonance condition at each band, using a center feed that preserves symmetry and consistent polarization.

#### 5.3.2. Radiation Characteristics and Implications for Coverage

A half-wave dipole exhibits a characteristic toroidal radiation pattern, with maximum radiation in the plane perpendicular to the antenna axis and nulls along the axis. This behavior is visible in Figure 10 (right), where the 3D pattern remains consistent across 2.4–6 GHz, and the azimuthal directivity cut (left) is close to uniform. Such a pattern is advantageous in outdoor access-point or gateway-like deployments where uniform horizontal coverage is desired.

#### 5.3.3. Justification for Simulation Use

Using a dipole as the transmit antenna provides a controlled baseline for analyzing propagation phenomena in a 3D urban/campus environment, because link variability is dominated by geometry and material interactions rather than highly directive antenna patterns. Moreover, prior studies have experimentally validated comparable half-wave dipole designs for Wi-Fi/WLAN bands, reporting gains close to the theoretical ∼2.15 dBi and radiation patterns consistent with analytical expectations [28,29]. Therefore, the MATLAB-generated dipole models offer a reproducible and physically meaningful antenna representation for the ray-tracing coverage simulations presented in this work.

### 5.4. MATLAB Data Collection Environment Setup

#### 5.4.1. Circular Receiver Array Generation

The circular receiver array represents a spatially symmetric configuration of receiver nodes uniformly distributed around a central transmitter. This topology enables isotropic coverage assessment and facilitates comparative signal strength analysis across multiple azimuth directions. The deployment is particularly suited for propagation modeling and interference analysis, where identical receiver conditions are desired around a reference point.

The array geometry is defined by a circular perimeter of radius *r*, centered at the base transmitter coordinates $\left(\phi_{0},\lambda_{0}\right)$. Each of the *N* receivers is placed at a constant angular separation of $\frac{360^{\circ }}{N}$, ensuring uniform spatial distribution. The position of each receiver is computed along a great-circle trajectory on the Earth’s surface using the geodesic direct problem formulation, as can be seen in Equation (6).

(6) $$\left(\phi_{i},\lambda_{i}\right)=\mathrm{reckon}\left(\phi_{0},\lambda_{0},\alpha ,\theta_{i}\right),$$

The expression defined in Equation (6) defines the geographic coordinates $\left(\phi_{i},\lambda_{i}\right)$ of the *i*-th receiver along a circular array using a geodesic reckoning MATLAB function. This operation computes a new location on the Earth’s surface given a starting point, an angular distance, and an azimuthal bearing. The parameters involved are described as follows:

- $\phi_{0}$: The latitude of the base (transmitter) site, expressed in degrees. This serves as the geodetic origin from which all receiver positions are derived.
- $\lambda_{0}$: The longitude of the base (transmitter) site, expressed in degrees. Together with $\phi_{0}$, it defines the center of the circular array.
- α: The angular distance (central angle) subtended by the circle at the Earth’s center, measured in degrees or radians. It is computed as described in Equation (7).
  (7) $$\alpha =\frac{r}{R_{⊕}},$$
  where *r* is the desired linear radius of the circular array (in meters), and $R_{⊕}$ is the mean Earth radius (approximately 6,371,000m). This conversion ensures accurate great-circle positioning even at non-negligible distances.
- $\theta_{i}$: The azimuth angle (or bearing) of the *i*-th receiver with respect to geographic north, measured clockwise in degrees. It determines the orientation of each receiver on the circular perimeter and is given by Equation (8).
  (8) $$\theta_{i}=\left(i-1\right)\frac{360^{\circ }}{N},$$
  where *N* is the total number of receivers in the array.
- $\left(\phi_{i},\lambda_{i}\right)$: The computed latitude and longitude of the *i*-th receiver site after applying the great-circle offset from the central transmitter.

In practical terms, the function reckon(·) in MATLAB uses a spherical Earth approximation to calculate the endpoint of a geodesic arc starting at $\left(\phi_{0},\lambda_{0}\right)$, traveling a surface distance corresponding to α, and following the azimuthal direction $\theta_{i}$. This ensures that all receiver sites are evenly distributed around the central node along a true geodesic circle, accounting for Earth curvature rather than relying on planar projections.

This geodesic approach ensures accurate placement of receivers even over large distances or in non-planar terrain, maintaining consistency with Earth curvature effects. Each receiver node is modeled as a measurement site characterized by its geographic coordinates, antenna parameters, and receiver sensitivity. In this study, a half-wave dipole antenna tuned to the operating frequency *f* was used for each receiver to approximate an omnidirectional radiation pattern suitable for general-purpose coverage evaluation.

The configuration defines an altitude threshold $h_{\max}$ that is later applied to filter out receivers positioned on top of buildings or other elevated structures. This parameter is not used during the initial array generation but serves as a post-processing criterion when populating a defined geographic area with receivers for data collection. By excluding high-elevation placements, the resulting receiver distribution remains representative of ground-level conditions, ensuring that signal measurements reflect realistic propagation environments within the selected coverage area.

**Figure 11:**
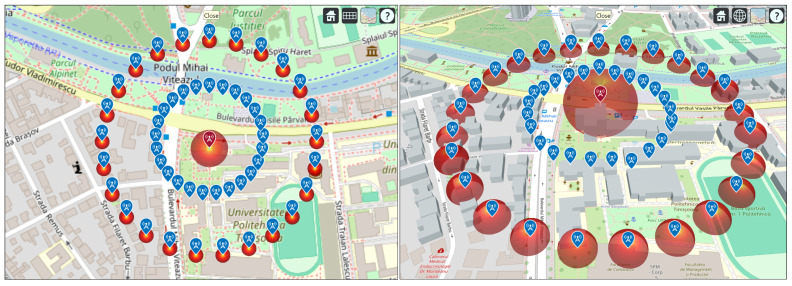
Circular receiver array MATLAB visualization around a central transmitter (Electro building B, Polytechnic University Timișoara Campus). The receivers are evenly distributed along a circular perimeter, with coverage visualization shown both in 2D (**left**) and 3D (**right**).

The resulting array forms a geodesically uniform sampling grid surrounding the transmitter, enabling comprehensive assessment of received signal strength and coverage uniformity in all directions. The geometry of the array is illustrated in Figure 11, which presents a two-dimensional overhead view (left) and a three-dimensional perspective (right) of the deployed receiver nodes around the transmitter, generated using MATLAB’s Site Viewer.

This formulation provides a mathematically consistent and computationally efficient method for generating a circular array of receivers suitable for evaluating coverage, power distribution, and signal degradation as a function of azimuth, elevation, and distance from the transmitter.

#### 5.4.2. Resolution-Driven Receiver Grid Filling via Concentric Circles

While the circular receiver array formulation in (6) establishes how a single ring of receivers can be placed around a transmitter, the practical dataset generation requires filling an entire area with receiver sites at a controllable spatial resolution. To achieve this, we implemented a resolution-driven sampling strategy that constructs multiple concentric circles around the transmitter and automatically adjusts the number of receivers per circle as a function of the desired receiver spacing. This produces an approximately uniform sampling density in the horizontal plane while preserving geodesic correctness.

A single parameter, denoted here by Δ (in meters), defines both: (i) the radial spacing between successive circles, and (ii) the arc-length spacing between adjacent receivers on the same circle. In our experiments, typical values were Δ∈{5m,2m}, corresponding to coarse and dense receiver sampling, respectively.

Given a maximum coverage radius $R_{\max}$, the number of concentric circles is computed as:

(9) $$N_{\mathrm{circles}}=\left\lfloor \frac{R_{\max}}{\Delta }\right\rfloor ,$$

so that circle *k* (with $k\in \{1,\ldots ,N_{\mathrm{circles}}\}$) has radius:

(10) $$r_{k}=k\Delta .$$

For each circle of radius $r_{k}$, the circumference is $2\pi r_{k}$. The number of receivers on that circle is selected to keep the arc-length separation close to Δ:

(11) $$N_{k}=\left\lfloor \frac{2\pi r_{k}}{\Delta }\right\rfloor .$$

Receivers are then placed at uniformly spaced azimuth angles,

(12) $$\theta_{k,i}=i\cdot \frac{360^{\circ }}{N_{k}},\;i\in \left\{1,\ldots ,N_{k}\right\},$$

and converted to geographic coordinates using MATLAB’s great-circle reckoning:

(13) $$\left(\phi_{k,i},\lambda_{k,i}\right)=\mathrm{reckon}\left(\phi_{0},\lambda_{0},\alpha_{k},\theta_{k,i}\right),\;\alpha_{k}=\frac{r_{k}}{R_{⊕}},$$

where $R_{⊕}$ denotes the mean Earth radius.

Although the circle construction is centered at the transmitter, we only retain receivers that belong to a user-defined target area, specified as a latitude/longitude polygon. For each candidate receiver, we apply an inclusion test:

(14) $$I_{\mathrm{poly}}\left(\phi_{k,i},\lambda_{k,i}\right)=\left\{\begin{array}{cc} 1, & \mathrm{if}\;(\phi_{k,i},\lambda_{k,i})\in P,\\ 0, & \mathrm{otherwise}, \end{array}\right.$$

implemented with MATLAB’s inpolygon() function (note that inpolygon() expects the input order (λ,ϕ)). Receivers outside the polygon are discarded, which allows the concentric-circle generator to fill arbitrary footprints (rectangular, campus-shaped, or street-bounded regions) while maintaining near-uniform sampling density.

In addition, we optionally enforce an elevation constraint to avoid placing receivers on elevated rooftops or terrain outliers:

(15) $$I_{h}\left(\phi_{k,i},\lambda_{k,i}\right)=\left\{\begin{array}{cc} 1, & h\left(\phi_{k,i},\lambda_{k,i}\right)\leq h_{\max},\\ 0, & \mathrm{otherwise}, \end{array}\right.$$

where h(·) is obtained via MATLAB’s elevation() query and $h_{\max}$ is a user-defined limit (disabled when $h_{\max}=-1$).

The final receiver set is therefore:

(16) $$R=\left\{\left(\phi_{k,i},\lambda_{k,i}\right)\;|\;I_{\mathrm{poly}}\left(\phi_{k,i},\lambda_{k,i}\right)=1\;\wedge \;I_{h}\left(\phi_{k,i},\lambda_{k,i}\right)=1\right\}.$$

**Figure 12:**
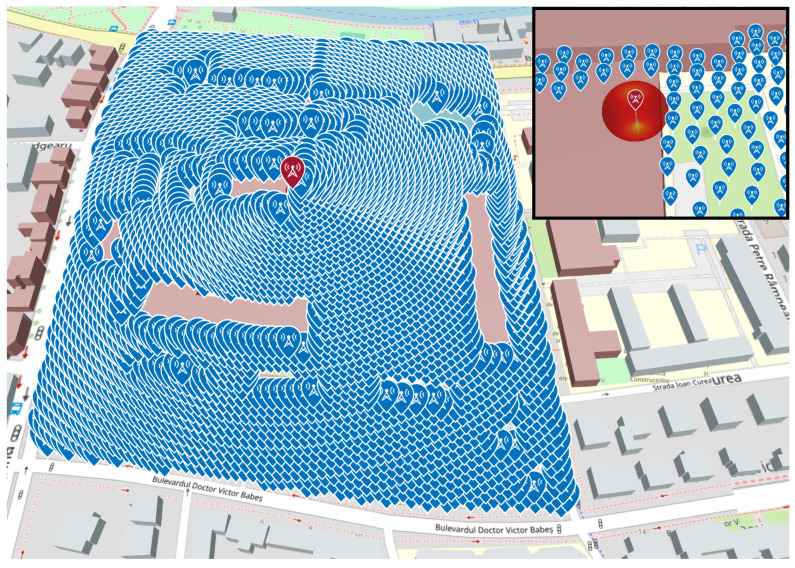
Receiver site generation using a resolution-driven concentric-circle strategy. Multiple circles are placed around the transmitter with radial spacing Δ, while the number of receivers per circle is computed from the circle circumference to maintain approximately Δ meters spacing along the arc. Receivers are retained only if they fall inside the user-defined polygon footprint (campus area) and satisfy an optional elevation threshold; the inset emphasizes the dense sampling near the transmitter.

Figure 12 illustrates the resulting receiver deployment over the campus area: the transmitter is centered in the region, and the receiver sites densely cover the polygon footprint using multiple rings. An inset view highlights how the grid density increases as Δ decreases (e.g., 2m vs. 5m), directly controlling the number of receivers and the total simulation workload.

This strategy enables controlled scaling of the dataset: reducing Δ increases both the number of circles and the number of receivers per circle, leading to a rapid growth in total receiver count (and thus ray-tracing calls). Consequently, Δ acts as the main knob for trading spatial fidelity against runtime and storage requirements, which is essential when generating millions of transmitter–receiver samples for subsequent machine learning regression.

### 5.5. Machine Learning Regression Strategy for RSSI Prediction

The objective of this study is to replace computationally expensive ray-tracing simulations with a data-driven surrogate model capable of accurately predicting the Received Signal Strength Indicator (RSSI) for Wi-Fi 7 links in a 3D urban environment. The regression task is formulated using ray-tracing outputs as ground-truth labels and aims to learn the functional relationship between transmitter–receiver geometry, propagation conditions, and received signal strength.

#### 5.5.1. Problem Formulation

Let $D={\left\{\left(x_{i},y_{i}\right)\right\}}_{i=1}^{n}$ denote a dataset of *n* wireless links generated through ray-tracing simulations. Each input vector $x_{i}\in R^{d}$ encodes the physical and geometric characteristics of the *i*-th transmitter–receiver pair, while the target value $y_{i}\in R$ corresponds to the simulated RSSI expressed in dBm.

The feature vector $x_{i}$ aggregates parameters that are known to influence radio propagation, including but not limited to the three-dimensional transmitter–receiver distance, operating frequency, transmit power, antenna heights, relative azimuth and elevation angles, and ray-tracing descriptors such as the number of reflections and diffractions. These features collectively describe the propagation scenario without explicitly modeling the electromagnetic interactions.

The learning objective is to estimate a parametric regression function:

(17) $${\hat{y}}_{i}=f_{\theta }\left(x_{i}\right),$$

where $f_{\theta }:R^{d}\to R$ is a nonlinear function parameterized by θ, and ${\hat{y}}_{i}$ denotes the predicted RSSI. The parameters θ are optimized by minimizing a regression loss over the dataset:

(18) $$\theta^{*}=\arg\min_{\theta }\frac{1}{n}\sum_{i=1}^{n}L\left(f_{\theta }\left(x_{i}\right),y_{i}\right),$$

where L(·) is a suitable loss function, such as the mean squared error.

From a physical perspective, the function $f_{\theta }$ acts as a surrogate for the ray-tracing propagation model by implicitly learning the combined effects of free-space attenuation, reflection, diffraction, and material interactions encoded in the input features. Once trained, the surrogate model enables rapid RSSI prediction for unseen transmitter–receiver configurations, reducing inference time from minutes or hours per scenario to milliseconds while preserving high-fidelity spatial coverage characteristics.

#### 5.5.2. Machine Learning Models

To approximate the ray-tracing propagation model, we evaluate several supervised regression approaches commonly used for structured and tabular data. Among these, the FT-Transformer is adopted as the primary model in this study due to its consistently superior predictive accuracy, training stability, and robustness across heterogeneous feature distributions. FT-Transformer extends transformer architectures to tabular regression by combining feature tokenization with self-attention, enabling it to capture complex nonlinear interactions between geometric, frequency-dependent, and propagation-related parameters.

For comparison, we also include the TabTransformer, which applies contextual embeddings to categorical and continuous features using self-attention, as well as a Random Forest Regressor representing classical ensemble-based machine learning. These models serve as strong baselines for assessing the benefit of attention-based architectures in learning high-dimensional propagation relationships.

In addition, selected gradient-boosting models such as LightGBM and XGBoost variants adapted for structured inputs, are optionally evaluated to provide further reference points. However, the main emphasis of the analysis remains on FT-Transformer, as it demonstrates the most favorable trade-off between prediction accuracy, generalization capability, and training stability for large-scale RSSI regression tasks.

#### 5.5.3. Transformer-Based Regression for RSSI Prediction

While ensemble tree methods and deep neural networks can accurately model nonlinear propagation effects, they are limited in their ability to capture long-range feature dependencies and contextual relationships between radio-environment variables. To overcome these limitations, we extended the regression framework with a transformer-based architecture capable of modeling feature interactions and spatial patterns relevant to the propagation process.

Transformers employ self-attention mechanisms that compute the contextual importance of each feature relative to others within a given sample. In the context of radio propagation, this allows the model to jointly reason about how parameters such as distance, frequency, transmitter height, and reflection count influence the received signal strength. Unlike convolutional or tree-based models, which primarily learn local or hierarchical relations, transformers learn global dependencies that generalize better across frequency bands, transmitter–receiver geometries, and urban layouts.

In terms of model architecture, we adopted a tabular transformer configuration inspired by the TabTransformer [11] and FT-Transformer [12] architectures. Each input feature $x_{i}=\left[x_{1},x_{2},\ldots ,x_{m}\right]$ was embedded into a *d*-dimensional vector space via a learnable linear projection:

(19) $$e_{j}=W_{j}x_{j}+b_{j},\;j=1,\ldots ,m$$

where $W_{j}\in R^{d}$ and $b_{j}$ are trainable parameters. The resulting set of feature embeddings $\left\{e_{1},\ldots ,e_{m}\right\}$ forms a token sequence processed by a stack of *L* self-attention layers:

(20) $$H^{\left(l\right)}=\mathrm{MSA}\left(\mathrm{LN}(H^{\left(l-1\right)})\right)+H^{\left(l-1\right)}$$

(21) $$H^{\left(l\right)}=\mathrm{MLP}\left(\mathrm{LN}(H^{\left(l\right)})\right)+H^{\left(l\right)}$$

where MSA denotes multi-head self-attention, LN denotes layer normalization, and MLP represents a feed-forward network. The final contextual embeddings are aggregated through average pooling and passed to a regression head producing two outputs:

(22) $$\left[{\hat{\mathrm{RSSI}}}_{1},{\hat{\mathrm{RSSI}}}_{2}\right]=W_{o}\cdot \mathrm{Pool}\left(H^{\left(L\right)}\right)+b_{o}$$

This setup supports both single- and multi-target regression, allowing simultaneous estimation of RSSI for dual-polarized or dual-band links.

The transformer model was trained using the AdamW optimizer with a cosine learning rate schedule and a mean-squared error (MSE) loss function. Dropout regularization (p=0.1) and early stopping were applied to prevent overfitting. Each training batch was normalized using feature-wise statistics from the training set only.

##### Advantages and Interpretability

Compared to conventional models, the transformer regressor offers several advantages:

- Contextual reasoning: Attention layers automatically weight relevant features (e.g., frequency and distance) depending on the environmental configuration.
- Multi-output generalization: A shared encoder can simultaneously predict multiple signal metrics (e.g., RSSI_1_, RSSI_2_) with cross-task regularization.
- Interpretability: Attention maps can be visualized to identify which propagation features most influence model predictions, providing an explainable framework complementary to SHAP analysis used for ensemble methods.

Empirically, the transformer-based regression achieved slightly lower RMSE than gradient boosting models on test links with high geometric variability, confirming its ability to generalize to unseen transmitter–receiver pairs.

### 5.6. FT-Transformer Architecture Diagram

**Figure 13:**
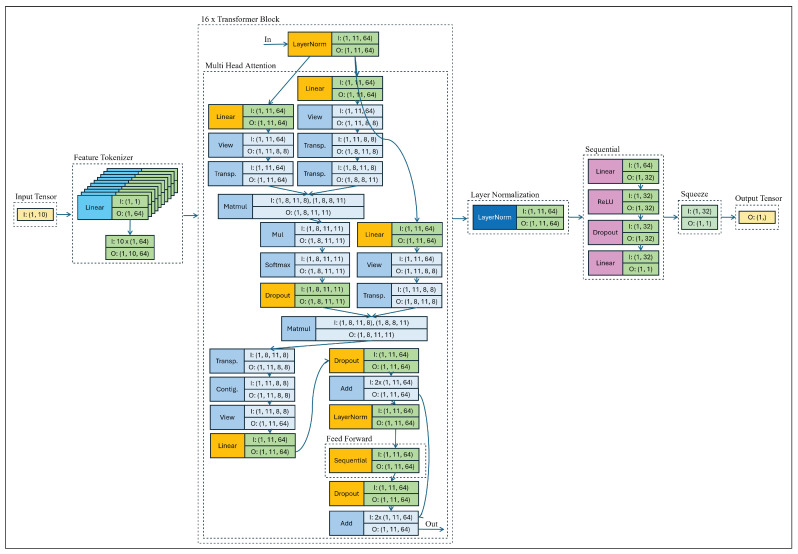
Detailed computational graph of the FT-Transformer architecture used for RSSI prediction. Each input feature is independently projected into a token embedding, followed by stacked Transformer encoder blocks with multi-head self-attention and residual connections. The [CLS] token representation is used for final regression. Tensor dimensions are shown at each processing stage.

Figure 13 illustrates the detailed computational flow of the adopted FT-Transformer architecture for structured tabular regression. The model follows a feature-tokenization paradigm, where each input feature is independently projected into a learnable embedding space before being processed by stacked self-attention layers.

Feature Tokenization. The input vector $x\in R^{F}$ (where *F* denotes the number of numerical features) is transformed into a sequence of token embeddings using individual linear projections. Each scalar feature is mapped through a learnable linear layer to a *d*-dimensional embedding, producing a tensor of shape (B,F,d), where *B* is the batch size and *d* is the token dimension. A learnable [CLS] token is prepended to the sequence, increasing the token length to F+1.

Multi-Head Self-Attention. The token sequence is processed by stacked Transformer encoder blocks. Each block begins with Layer Normalization, followed by Multi-Head Self-Attention (MHSA). Queries, keys, and values are computed through linear projections and reshaped into multiple attention heads. The scaled dot-product attention mechanism computes pairwise interactions between feature tokens, enabling the model to capture global feature dependencies. The outputs of all heads are concatenated and projected back to the token dimension.

Residual Connections and Feed-Forward Network. A residual connection is applied after the attention module, followed by another Layer Normalization layer. The Feed-Forward Network (FFN) consists of two linear transformations with a nonlinear activation (ReLU) and optional dropout regularization. This stage refines token representations independently while preserving inter-feature interactions captured by attention. A second residual connection completes the Transformer block.

Prediction Head. After the final Transformer layer, the representation corresponding to the [CLS] token is extracted as a global summary embedding. This embedding is passed through a fully connected regression head consisting of linear layers and nonlinear activation to produce the final RSSI prediction.

Overall, the architecture enables joint modeling of complex nonlinear relationships among geometric, frequency-dependent, environmental, and multipath interaction features through self-attention, while maintaining permutation invariance across structured tabular inputs.

### 5.7. Evaluation Metrics

#### 5.7.1. Coefficient of Determination ($R^{2}$)

To quantitatively assess the quality of the RSSI regression models, we employ the coefficient of determination, denoted as $R^{2}$. This metric measures how well the predicted values approximate the observed data by quantifying the proportion of variance in the target variable that is explained by the regression model.

Let ${\left\{y_{i}\right\}}_{i=1}^{n}$ denote the ground-truth RSSI values obtained from ray-tracing simulations, ${\left\{{\hat{y}}_{i}\right\}}_{i=1}^{n}$ the corresponding model predictions, and $\bar{y}$ the mean of the true values:

(23) $$\bar{y}=\frac{1}{n}\sum_{i=1}^{n}y_{i}$$

The total variability present in the data is captured by the total sum of squares (SST),

(24) $$\mathrm{SST}=\sum_{i=1}^{n}{\left(y_{i}-\bar{y}\right)}^{2}$$

which represents the variance of the target variable around its mean. The portion of this variability that remains unexplained by the model is quantified by the sum of squared errors (SSE),

(25) $$\mathrm{SSE}=\sum_{i=1}^{n}{\left(y_{i}-{\hat{y}}_{i}\right)}^{2}$$

while the variability explained by the regression model is given by the regression sum of squares (SSR),

(26) $$\mathrm{SSR}=\sum_{i=1}^{n}{\left({\hat{y}}_{i}-\bar{y}\right)}^{2}$$

**Figure 14:**
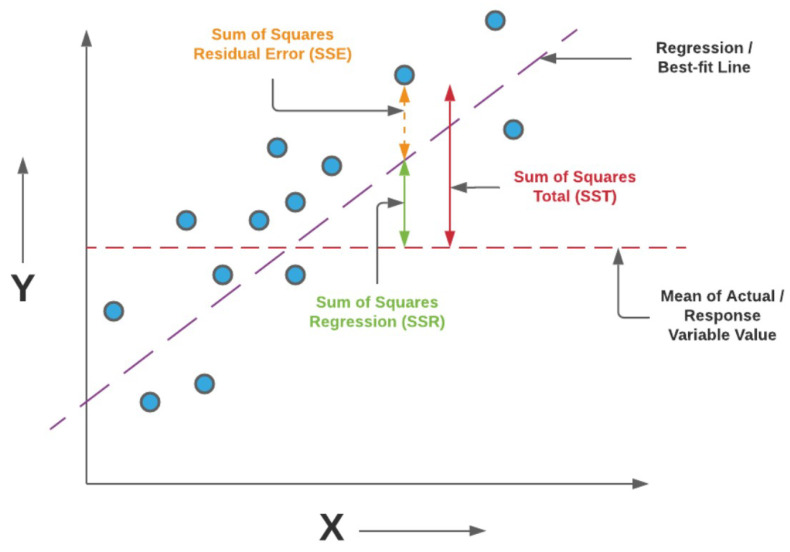
Illustration of the decomposition of variance in linear regression. The total sum of squares (SST) measures the variance of the observed data around the mean, the regression sum of squares (SSR) represents the variance explained by the model, and the sum of squared errors (SSE) captures the residual error. The coefficient of determination $R^{2}$ quantifies the ratio of explained variance to total variance (source: https://medium.com/geekculture/decomposition-of-variability-da4ba31b4ceb, accessed on 23 March 2026).

These quantities satisfy the identity SST=SSR+SSE; see Figure 14 for more details.

The coefficient of determination is then defined as:

(27) $$R^{2}=\frac{\mathrm{SSR}}{\mathrm{SST}}=1-\frac{\mathrm{SSE}}{\mathrm{SST}}=1-\frac{\sum_{i=1}^{n}{\left(y_{i}-{\hat{y}}_{i}\right)}^{2}}{\sum_{i=1}^{n}{\left(y_{i}-\bar{y}\right)}^{2}}$$

An $R^{2}$ value of 1 indicates perfect agreement between predictions and ground truth, meaning that the model explains all variance in the data. An $R^{2}$ value of 0 corresponds to a model whose predictive performance is equivalent to using the mean of the target variable as a constant predictor. Negative values of $R^{2}$ may occur when the model performs worse than this baseline, indicating poor generalization.

In the context of RSSI prediction, a high $R^{2}$ score implies that the regression model effectively captures the complex relationships between geometric configuration, frequency, antenna parameters, and propagation effects learned from ray-tracing simulations. As such, $R^{2}$ provides a meaningful measure of how accurately the surrogate model reproduces spatial signal strength variations across the deployment area.

**Figure 15:**
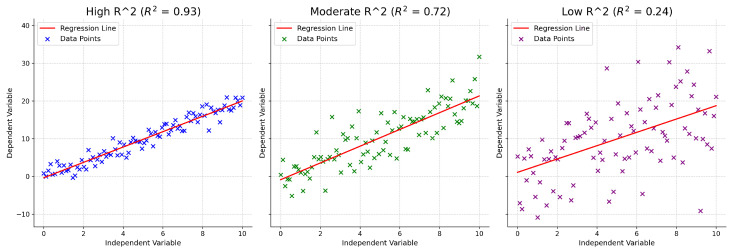
Illustrative examples of regression performance for different values of the coefficient of determination $R^{2}$. (**Left**): High $R^{2}$, where predictions closely follow the regression line and most variance is explained. (**Center**): Moderate $R^{2}$, indicating partial explanatory power with noticeable dispersion. (**Right**): Low $R^{2}$, where predictions exhibit weak alignment with observations and most variance remains unexplained.

To further illustrate the interpretation of the coefficient of determination, Figure 15 provides representative regression scenarios corresponding to high, moderate, and low $R^{2}$ values. These examples highlight how the magnitude of $R^{2}$ reflects the degree to which the model captures the variability of the target variable.

In the high-$R^{2}$ case (left), the predicted values closely follow the regression line, and the data points exhibit minimal dispersion around it. This indicates a strong relationship between the input features and the target variable, with most of the variance in the observations being explained by the model. In practical terms, such a result suggests that the regression model successfully learns the dominant factors governing signal strength variations.

The moderate-$R^{2}$ scenario (center) shows increased scatter around the regression line. While a clear trend is still present, a non-negligible portion of the variance remains unexplained. This behavior is typical of propagation scenarios where additional environmental factors or nonlinear interactions contribute to signal variability that is not fully captured by the input features.

In the low-$R^{2}$ case (right), the data points are widely dispersed with respect to the regression line, indicating a weak relationship between the predictors and the target variable. Here, the model explains only a small fraction of the total variance, suggesting limited predictive capability and poor generalization. In the context of RSSI prediction, such outcomes may arise from insufficient feature representation, excessive noise, or propagation effects that are not adequately encoded in the model inputs.

In this work, the coefficient of determination is reported separately for the training, validation, and test datasets, each of which is designed to probe a distinct aspect of model learning and generalization.

The training $R^{2}$ quantifies how well the regression model fits the baseline ray-tracing data used for parameter optimization. The training set comprises simulations conducted at 2.4, 5, and 6 GHz with a receiver spacing of 5 m and transmit power levels of 100, 200, 500, and 1000 mW. High training $R^{2}$ values indicate that the model successfully captures the dominant propagation relationships present in the multi-frequency, multi-power baseline configuration. However, excessively high training scores relative to validation performance may also signal overfitting to specific spatial or frequency-dependent patterns.

The validation $R^{2}$ evaluates the model’s ability to generalize across unseen frequency channels and operating conditions while preserving the same spatial sampling resolution. For validation, one representative channel is selected from each band—2.447 GHz (channel 8) in the 2.4 GHz band, 5.15 GHz in the 5 GHz band, and 6.905 GHz (320 MHz channel) in the 6 GHz band—all simulated at a transmit power of 1000 mW and with a receiver spacing of 5 m. This setup primarily probes the model’s capacity to interpolate across frequency and channel bandwidth variations within each band. A validation $R^{2}$ close to the training value indicates robust spectral generalization, whereas a significant drop suggests sensitivity to frequency-specific propagation effects.

The test $R^{2}$ isolates the model’s ability to generalize spatially by evaluating predictions on a denser receiver deployment than that used during training and validation. The test dataset uses the same frequencies and transmit power as the validation set but reduces the receiver spacing from 5 m to 2 m, introducing a substantially finer spatial sampling of the propagation environment. In this scenario, $R^{2}$ measures how well the learned propagation relationships extrapolate to previously unseen receiver locations rather than new spectral conditions. Strong test performance indicates that the model captures physically meaningful propagation trends and spatial smoothness, while degradation in test $R^{2}$ reveals limitations in modeling fine-grained spatial variability.

Taken together, the evolution of $R^{2}$ from training to validation and testing provides insight into the model’s capacity to generalize across frequency, power, and spatial resolution. This stratified evaluation is particularly relevant for digital twin applications, where surrogate models must remain reliable when queried at resolutions and configurations that were not explicitly simulated during training.

#### 5.7.2. Mean Absolute Error (MAE)

While the coefficient of determination ($R^{2}$) provides insight into the proportion of variance explained by the model, it does not directly quantify the magnitude of prediction errors. To complement this analysis, we evaluate model performance using the Mean Absolute Error (MAE), which measures the average absolute deviation between predicted and observed RSSI values. MAE is defined in Equation (28).

(28) $$\mathrm{MAE}=\frac{1}{n}\sum_{i=1}^{n}\left|y_{i}-{\hat{y}}_{i}\right|$$

where:

- *n* is the total number of samples;
- $y_{i}$ denotes the true RSSI value obtained from ray-tracing simulations for the *i*-th receiver;
- ${\hat{y}}_{i}$ represents the corresponding RSSI predicted by the machine learning model.

Unlike squared-error metrics, MAE penalizes all errors linearly and is therefore less sensitive to large outliers. This property is particularly relevant in urban wireless propagation scenarios, where occasional deep fades or extreme shadowing conditions may occur due to complex multipath interactions. As a result, MAE provides a robust and interpretable estimate of the typical prediction error expressed directly in decibels (dB), facilitating intuitive assessment of model accuracy.

In this study, MAE is reported separately for training, validation, and testing datasets. During training, MAE reflects the model’s ability to fit baseline ray-tracing simulations across multiple frequencies and transmit power levels. Validation MAE assesses interpolation performance across unseen channels within the same spatial resolution, while test MAE evaluates generalization to higher-resolution receiver grids. Together with $R^{2}$, MAE offers a complementary perspective on both the accuracy and stability of the learned surrogate models.

#### 5.7.3. Root Mean Squared Error (RMSE)

The Root Mean Squared Error (RMSE) is a scale-dependent regression metric that quantifies the average magnitude of prediction errors by penalizing larger deviations more strongly. Due to its sensitivity to outliers and its expression in the same physical units as the target variable, RMSE is particularly well suited for evaluating wireless signal strength prediction accuracy, where large RSSI errors may have a disproportionate impact on coverage and link reliability.

RMSE is derived from the Mean Squared Error (MSE), which measures the average of the squared residuals between the predicted and observed values, as represented in Equation (29).

(29) $$\mathrm{MSE}=\frac{1}{n}\sum_{i=1}^{n}{\left(y_{i}-{\hat{y}}_{i}\right)}^{2}$$

where $y_{i}$ denotes the ground-truth RSSI value obtained from ray-tracing simulations, ${\hat{y}}_{i}$ is the corresponding model prediction, and *n* is the total number of samples. The RMSE is then defined as the square root of MSE (Equation (30)).

(30) $$\mathrm{RMSE}=\sqrt{\frac{1}{n}\sum_{i=1}^{n}{\left(y_{i}-{\hat{y}}_{i}\right)}^{2}}$$

**Figure 16:**
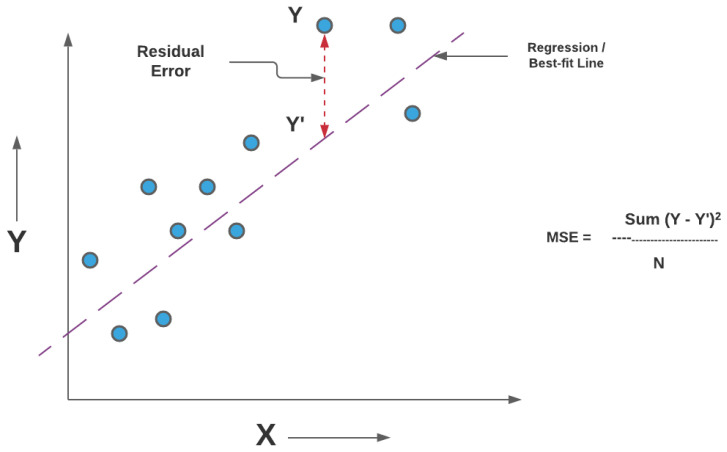
Illustration of residual errors in a regression model and their contribution to the mean squared error (MSE), which forms the basis of the Root Mean Squared Error (RMSE). Image source: https://encord.com/glossary/mean-square-error-mse/ (accessed on 23 March 2026).

Figure 16 illustrates the geometric interpretation of squared residuals in a regression setting. Each residual corresponds to the vertical distance between an observed value and the regression estimate, and squaring these residuals emphasizes larger prediction errors. This property makes RMSE more sensitive than MAE to occasional large mismatches, such as those caused by complex multipath propagation, diffraction, or abrupt shadowing effects in dense urban environments.

By taking the square root of MSE, RMSE restores the metric to the original unit of the target variable (dB), enabling direct physical interpretation. In the context of this study, RMSE represents the expected deviation (in dB) between transformer-based predictions and ray-tracing-derived RSSI values. Lower RMSE values therefore indicate a more accurate surrogate model capable of reproducing fine-grained propagation behavior across frequencies, power levels, and spatial resolutions.

Unlike MAE, which weights all errors equally, RMSE assigns disproportionately higher penalties to large deviations. In the context of wireless propagation modeling, such deviations often correspond to challenging conditions such as non-line-of-sight links, strong diffraction effects, or deep shadowing caused by building obstructions. Consequently, RMSE is particularly effective at highlighting whether a model occasionally fails in difficult propagation scenarios, even when average performance remains acceptable.

In this study, RMSE is reported for training, validation, and test datasets alongside MAE and $R^{2}$. Consistent trends between MAE and RMSE indicate stable and well-behaved predictions, whereas a substantially higher RMSE relative to MAE suggests the presence of localized high-error regions. This joint analysis enables a more nuanced assessment of model robustness and suitability for high-resolution digital twin applications.

### 5.8. Dataset Construction and Organization

This section describes the structure, generation process, and partitioning strategy of the datasets used for training, validation, and testing the Machine Learning models. All datasets are derived from high-fidelity MATLAB ray-tracing simulations and are organized to support systematic evaluation across frequency bands, transmit power levels, and spatial resolutions.

#### 5.8.1. Training Dataset

**Figure 17:**
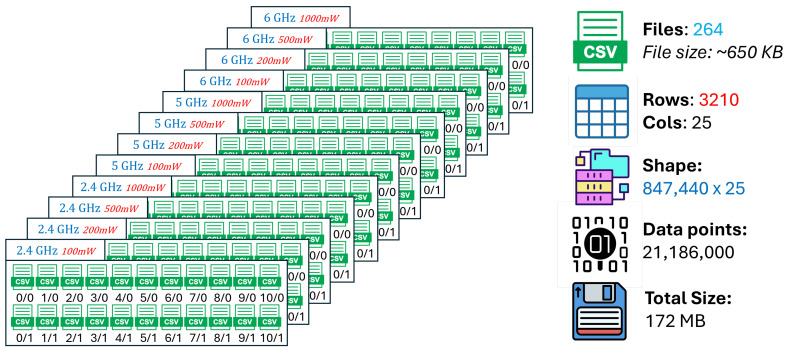
Overview of the training dataset organization. Ray-tracing simulations are generated across three frequency bands (2.4, 5, and 6 GHz) and four transmit power levels (100, 200, 500, and 1000 mW). Each configuration produces multiple CSV files corresponding to different reflection–diffraction (R/D) settings, forming the complete training data corpus.

The training dataset is composed of a comprehensive collection of ray-tracing simulation outputs generated across multiple Wi-Fi frequency bands and transmit power configurations, as illustrated in Figure 17. The objective of the training set is to expose the learning models to a wide range of propagation conditions, enabling them to capture generalizable relationships between geometric, environmental, and radio-frequency parameters and the resulting RSSI values.

Specifically, simulations were conducted at three carrier frequencies corresponding to the 2.4 GHz, 5 GHz, and 6 GHz Wi-Fi bands. For each frequency, four transmit power levels were considered: 100 mW, 200 mW, 500 mW, and 1000 mW. This results in a total of twelve distinct frequency–power configurations forming the backbone of the training data. For each configuration, receiver locations were generated using a circular multi-ring placement strategy with a fixed spatial resolution of 5 m between adjacent receivers, ensuring uniform spatial coverage of the defined urban area.

Each frequency–power combination produces multiple CSV files, corresponding to different ray-tracing configurations defined by the maximum number of reflections and diffractions allowed in the propagation model. As depicted in Figure 17, these configurations are indexed in the form (R/D), where *R* denotes the maximum number of reflections and *D* the maximum number of diffractions. This structured organization allows the model to learn not only smooth line-of-sight attenuation trends, but also complex multipath effects associated with higher-order interactions.

All training samples include a consistent set of input features describing transmitter parameters, receiver geometry, relative positioning, angular relationships, and ray-tracing interaction statistics, while the target variables correspond to the simulated RSSI values. By aggregating data across frequencies, power levels, and propagation complexities, the training dataset provides a diverse and information-rich foundation for learning a robust surrogate model capable of approximating detailed ray-tracing behavior.

#### 5.8.2. Evaluation (Validation) Dataset

**Figure 18:**
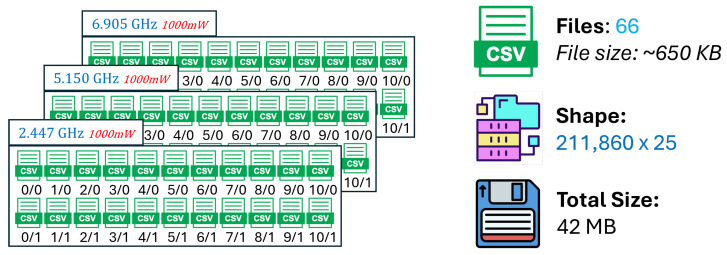
Organization of the evaluation (validation) dataset. Ray-tracing simulations are generated for three representative channels at 2.4 GHz (2.447 GHz), 5 GHz (5.15 GHz), and 6 GHz (6.905 GHz), all at a fixed transmit power of 1000 mW. Each configuration includes multiple reflection–diffraction (R/D) settings, resulting in a structured set of CSV files used for validation.

The evaluation (validation) dataset is designed to assess the model’s ability to generalize beyond the frequency–power combinations explicitly seen during training, while preserving comparable propagation complexity and spatial resolution. An overview of the validation data organization is shown in Figure 18.

In contrast to the training dataset, which spans multiple power levels and dense frequency coverage, the validation dataset consists of a reduced but representative subset of operating points. Specifically, three single-channel configurations were selected, one from each Wi-Fi band: channel 8 at 2.4 GHz (2.447 GHz), a representative channel in the 5 GHz band (5.15 GHz), and a wideband 6 GHz channel centered at 6.905 GHz. All validation simulations were performed at a fixed transmit power of 1000 mW to isolate the effect of frequency generalization.

For each frequency, multiple ray-tracing configurations were generated by varying the maximum number of reflections and diffractions allowed in the propagation model. As in the training dataset, these configurations are indexed using (R/D) notation, where *R* denotes the reflection order and *D* the diffraction order. This structure ensures that the validation set contains both low-complexity and multipath-rich propagation scenarios, enabling a robust assessment of model performance across different interaction regimes.

Receiver locations in the validation dataset follow the same circular multi-ring placement strategy as the training data, using a spatial resolution of 5 m. This consistency allows performance differences between training and validation to be attributed primarily to unseen frequency configurations rather than changes in spatial sampling density.

Overall, the validation dataset comprises a limited number of frequency–power combinations but a wide range of propagation complexities, resulting in a compact yet challenging benchmark. Performance on this dataset provides an early indication of the model’s ability to interpolate across frequency bands while maintaining stability under realistic ray-tracing conditions.

#### 5.8.3. Test Dataset

**Figure 19:**
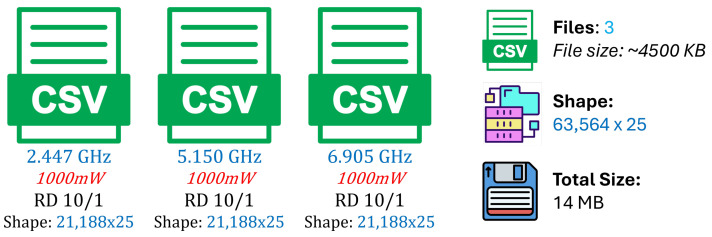
Organization of the test dataset. High-resolution ray-tracing simulations are generated at 2.447 GHz, 5.15 GHz, and 6.905 GHz, all at a transmit power of 1000 mW, using a finer receiver spacing of 2 m. Each configuration produces a large CSV file containing dense spatial RSSI samples used to assess spatial generalization performance.

The test dataset is constructed to evaluate the model’s ability to generalize across spatial resolution, rather than across frequency or transmit power. An overview of the test data organization is shown in Figure 19. This dataset represents the most challenging evaluation scenario, as it probes the surrogate model’s performance at receiver locations that were not explicitly observed during training or validation.

The test dataset uses the same three frequency–power configurations as the validation set, namely 2.447 GHz (channel 8 in the 2.4 GHz band), 5.15 GHz (5 GHz band), and 6.905 GHz (6 GHz band), all simulated at a fixed transmit power of 1000 mW. By keeping frequency and power constant, differences in predictive performance can be directly attributed to changes in spatial sampling density.

In contrast to the training and validation datasets, where receiver locations are generated with a spatial resolution of 5 m, the test dataset employs a finer receiver placement resolution of 2 m. This results in a substantially larger number of receiver locations within the same geographic area, effectively increasing the spatial granularity of the propagation field. As illustrated in Figure 19, each frequency configuration yields a large CSV file containing high-resolution RSSI measurements and associated propagation features.

All test samples are generated using the same ray-tracing configurations and feature definitions as the training and validation datasets, ensuring full compatibility at the input level. However, the denser receiver grid introduces receiver positions that do not coincide with those seen during training, thereby enforcing genuine spatial interpolation by the learning models.

Performance on this test dataset provides a critical measure of spatial generalization capability, which is essential for digital twin applications where coverage predictions may be queried at arbitrary locations and resolutions beyond those used during simulation or model training.

#### 5.8.4. Dataset Overview and Partitioning

**Figure 20:**
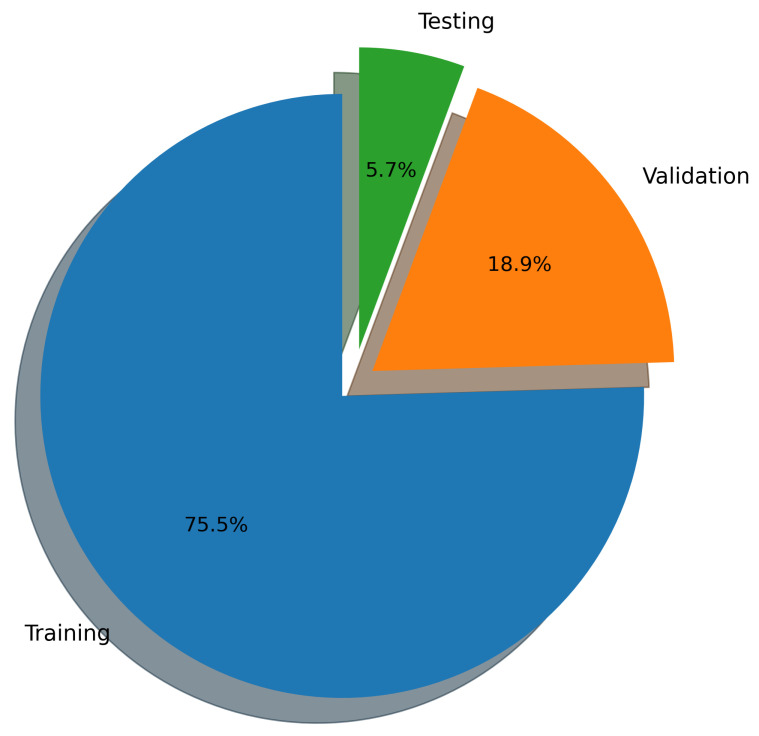
Overall dataset composition showing the proportion of samples used for training (847,440), validation (211,860), and testing (63,564).

Figure 20 summarizes the overall composition of the dataset used in this study, highlighting the relative proportions of training, validation, and test samples. In total, the dataset comprises more than one million ray-tracing-derived samples, each described by a consistent set of input features and corresponding RSSI targets.

The training dataset constitutes the majority of the data, with 847,440 samples (approximately 75.5% of the total), reflecting its role in learning robust and generalizable propagation patterns across multiple frequencies, transmit power levels, and ray-tracing configurations. The validation dataset contains 211,860 samples (18.9%), drawn from unseen frequency configurations at a fixed transmit power, and is used to guide model selection and hyperparameter tuning. Finally, the test dataset consists of 63,564 samples (5.7%), generated at a higher spatial resolution to evaluate spatial generalization beyond the receiver locations observed during training.

This stratified partitioning strategy ensures a clear separation between learning, model selection, and final evaluation phases. Moreover, the progressive reduction in dataset size from training to testing reflects an intentional increase in task difficulty, transitioning from dense multi-configuration learning to high-resolution spatial extrapolation. Such a design closely mirrors practical digital twin deployment scenarios, where surrogate models trained on large-scale simulations must deliver accurate predictions at novel locations and resolutions.

### 5.9. Machine Learning Pipeline

#### 5.9.1. Overview

**Figure 21:**
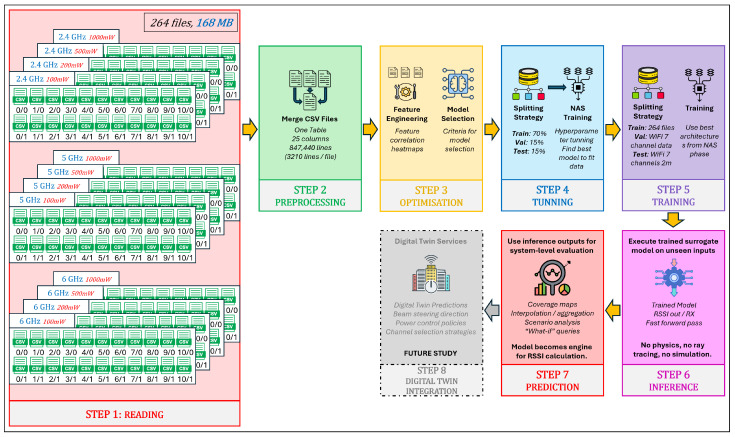
End-to-end machine learning pipeline for RSSI surrogate modeling. The workflow spans from large-scale ray-tracing data ingestion and preprocessing (Steps 1–2), through feature engineering, model selection, and NAS-based tuning (Steps 3–4), to model training and fast inference (Steps 5–6). Prediction outputs are used for coverage analysis and system-level evaluation (Step 7), with a forward-looking integration into wireless digital twin services and network intelligence applications (Step 8).

Figure 21 illustrates the complete Machine Learning pipeline adopted in this study, presenting an end-to-end workflow that spans from large-scale ray-tracing data generation to fast RSSI inference and its integration within a digital twin context. The pipeline is deliberately structured to decouple computationally expensive physics-based simulations from downstream learning, evaluation, and system-level optimization tasks.

The workflow begins with Step 1 (Reading), where ray-tracing outputs generated for multiple frequencies (2.4, 5, and 6 GHz), transmit power levels, and propagation configurations are stored as individual CSV files. Collectively, these files capture a dense spatial sampling of the wireless environment and form the raw input to the learning process.

In Step 2 (Preprocessing), all simulation outputs are merged into a single structured dataset with a fixed schema of 25 features per receiver location. This step ensures consistency across frequencies, power levels, and spatial resolutions, while also preparing the data for statistical analysis and machine learning through normalization and integrity checks.

Step 3 (Optimization) focuses on feature engineering and model selection. Feature correlation analysis and relevance inspection are used to identify informative propagation descriptors, while candidate regression models are shortlisted based on robustness, scalability, and suitability for tabular wireless data.

The learning process is refined in Step 4 (Tuning), where a Neural Architecture Search (NAS)-driven strategy is employed to optimize model hyperparameters and architectures. The dataset is partitioned into training, validation, and test subsets according to the strategy described in Section 5.8, enabling controlled evaluation across frequencies, transmit power levels, and spatial resolutions.

In Step 5 (Training), the best-performing architectures identified during the tuning phase are trained on the full training dataset. This step yields surrogate models capable of approximating ray-tracing outputs with high fidelity, while significantly reducing computational complexity.

Once trained, the models are deployed in Step 6 (Inference), where RSSI predictions are generated for previously unseen inputs using a single forward pass through the network. This step replaces repeated ray-tracing simulations with near-instantaneous predictions, enabling rapid exploration of large spatial domains.

Step 7 (Prediction) leverages inference outputs to construct higher-level system representations, including coverage maps, interpolated RSSI fields, and aggregated performance metrics. At this stage, the learned model effectively acts as an RSSI computation engine, supporting scenario-based and “what-if” analyses without invoking physics-based solvers.

Finally, Step 8 (Digital Twin Integration) illustrates how the proposed surrogate modeling framework can be embedded within a broader wireless digital twin architecture. Predicted RSSI fields can serve as inputs for advanced network intelligence tasks such as beam steering optimization, power control policy evaluation, channel selection strategies, and large-scale scenario assessment. Although presented as a forward-looking extension, this step highlights the potential of the proposed model as a foundational component for AI-native wireless digital twins.

#### 5.9.2. Feature Correlation Analysis

To better understand the relationships between ray-tracing-derived input variables and the received signal strength, a comprehensive feature correlation analysis was performed prior to model training. The objective of this analysis is not feature construction, but rather interpretability, redundancy assessment, and physical validation of the simulated propagation behavior.

Correlation coefficients were computed between each input feature and the two target variables, RSSI1 and RSSI2, using three complementary statistical measures: Pearson, Spearman, and Kendall correlations. Pearson correlation captures linear dependencies, while Spearman and Kendall correlations assess monotonic relationships and are more robust to nonlinear effects and outliers. Employing all three metrics provides a more complete characterization of feature–target dependencies in complex radio environments.

**Figure 22:**
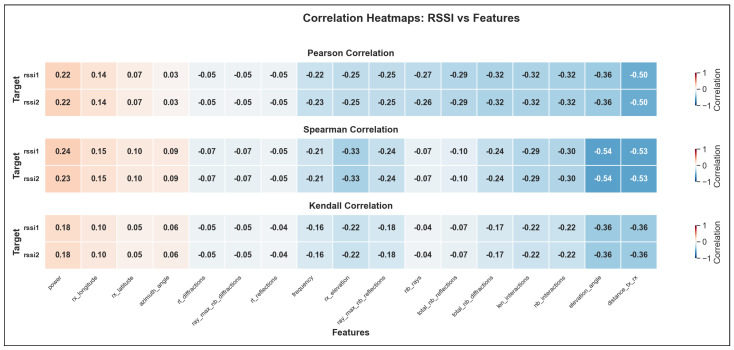
Feature–target correlation heatmaps showing Pearson, Spearman, and Kendall correlation coefficients between ray-tracing-derived input features and the RSSI targets (RSSI1, RSSI2). Positive and negative correlations reflect physical propagation effects such as transmit power scaling, distance-dependent path loss, frequency-dependent attenuation, and multipath interactions.

Figure 22 illustrates the resulting correlation heatmaps, with RSSI targets shown on the vertical axis and input features on the horizontal axis. A total of 25 columns are present in the dataset; however, six features contained only missing values in the analyzed subset and were excluded from the visualization. The two target variables themselves (RSSI1 and RSSI2), as well as auxiliary metadata fields such as receiver identifiers and elapsed simulation time, were also omitted, resulting in 23 physically meaningful input features used in the correlation analysis.

Strong negative correlations are observed for distance-related features, particularly the transmitter–receiver separation, which exhibits the highest magnitude correlation across all three methods. This behavior is consistent with established path-loss models, where received power decays logarithmically with distance. Similarly, frequency shows a moderate negative correlation with RSSI, reflecting increased free-space and material attenuation at higher carrier frequencies. Features related to ray complexity, such as the number of interactions, reflections, and diffractions, also display negative correlations, indicating increased signal degradation in non-line-of-sight and multipath-dominated scenarios.

Conversely, transmit power exhibits a positive correlation with RSSI, as expected from basic link budget considerations. Geometric parameters such as elevation and azimuth angles show weaker but non-negligible correlations with the received signal strength. In the present study, these angular features do not reflect changes in antenna orientation or steering, which are fixed, but instead arise from the relative transmitter–receiver geometry. As such, their influence on RSSI is indirect and mediated through propagation conditions such as line-of-sight availability, blockage, and the presence of reflections or diffractions, resulting in relationships that are not strictly linear. The consistency of correlation signs across Pearson, Spearman, and Kendall metrics supports the robustness of these observations under the adopted simulation configuration.

Importantly, no features were removed solely based on correlation magnitude. The purpose of the correlation analysis is not feature elimination, but validation of the physical plausibility and internal consistency of the simulated dataset. This analysis also informs subsequent model selection by highlighting potential dependencies and interactions among variables. Transformer-based architectures, in particular, are well suited to handling correlated and interacting features without requiring aggressive manual feature pruning. Consequently, all physically meaningful ray-tracing outputs are retained to preserve the descriptive richness of the data.

Angular features, particularly the azimuth angle, exhibit relatively weak correlations with the RSSI targets in the current dataset. This behavior is expected given the experimental design adopted in this study. All simulations employ a fixed, center-fed half-wave dipole antenna positioned at a static location with a fixed orientation. As a result, azimuth and elevation angles vary only as a function of receiver placement, rather than as controllable transmission parameters. Under these conditions, angular variables primarily encode spatial geometry rather than directional gain, leading to a limited direct influence on received power.

In future studies, where antenna placement, orientation, and beam steering are explicitly varied across the urban environment, angular features are expected to play a substantially more prominent role. In such scenarios, azimuth and elevation angles will directly interact with antenna radiation patterns, polarization effects, and directional gain, thereby exerting stronger influence on link quality and coverage characteristics. The inclusion of angular features in the present study therefore serves both as a geometric descriptor of receiver positioning and as a forward-compatible design choice, enabling seamless extension of the framework toward adaptive antennas, directional transmissions, and digital twin-driven network optimization.

#### 5.9.3. NAS-Style Hyperparameter Exploration for FT-Transformer

To obtain a robust and high-performing surrogate for RSSI prediction, we adopted a NAS-style (Neural Architecture Search) exploration strategy centered on systematic hyperparameter sweeping for the FT-Transformer. Rather than searching over arbitrary network graphs, the exploration is formulated as a discrete parameter-grid search over architectural capacity and training dynamics, where each configuration is trained and evaluated under an identical protocol and logged for later ranking. This approach is well aligned with tabular transformer regressors, whose accuracy and stability are strongly influenced by token dimensionality, attention configuration, and depth [12].

Search space (parameter grid). The explored grid spans tokenization width, transformer depth/width, nonlinearity choice, and regularization/optimization parameters:

$d_{\mathrm{token}}$: {16,32,64,96,128,192,256,512}
*L*: {2,3,4,5,6,8}
*H*: {1,2,4,8,16,32}
$d_{\mathrm{ff}}$: $\alpha \cdot d_{\mathrm{token}},\;\alpha \in \left\{2,3,4,6,8\right\}$
$p_{\mathrm{drop}}$: {0.0,0.1,0.2}
activation: {ReLU,GELU}
batch size: 256
η: $\left\{10^{-4},5\;\cdot \;10^{-4},10^{-3},2\;\cdot \;10^{-3},3\;\cdot \;10^{-3}\right\}$
$\lambda_{\mathrm{wd}}$: $\left\{0,10^{-5},10^{-4},10^{-3}\right\}$

Here, $d_{\mathrm{token}}$ controls the dimensionality of each feature token, *L* is the number of transformer blocks, *H* is the number of attention heads, and $d_{\mathrm{ff}}$ is the hidden width of the feed-forward sublayer. The dropout probability $p_{\mathrm{drop}}$, learning rate η, and weight decay $\lambda_{\mathrm{wd}}$ govern regularization and optimization. The batch size is fixed to 256 due to GPU memory constraints in large-scale sweeps.

The full grid contains

|G|=8×6×6×5×3×2×1×5×4=17,280

candidate configurations. To make this exploration tractable, we execute the NAS script in parallel batches, typically fixing a single $d_{\mathrm{token}}$ value per run (e.g., $d_{\mathrm{token}}=16$, then 32, 64, etc.) while sweeping the remaining hyperparameters. This yields a clear stratified comparison of model capacity versus generalization as $d_{\mathrm{token}}$ increases, and it avoids creating an excessively large monolithic job. For reproducibility and traceability, each run writes a meta-dataset in .csv format, and the output filename suffix encodes the selected $d_{\mathrm{token}}$ (and optionally other run identifiers), allowing straightforward aggregation and ranking across runs.

The FT-Transformer is implemented in PyTorch 2.8.0 using a pre-norm transformer backbone. Continuous radio-environment features are mapped to a sequence of learnable feature tokens via feature-wise linear projections and additive feature biases. A learnable [CLS] token is prepended to provide a global aggregation mechanism. The resulting token sequence is processed by *L* stacked blocks, each consisting of multi-head self-attention (MSA) and a position-wise feed-forward network (FFN) with residual connections and dropout. The final [CLS] embedding is passed to a regression head to output the predicted RSSI. Given the heterogeneous magnitudes of ray-tracing features, robust scaling is applied before training. Optimization uses AdamW with learning-rate scheduling and early stopping to improve stability across thousands of candidate configurations.

For each grid configuration, we log split-wise performance ($R^{2}$, MAE, RMSE) on training, validation, and test sets. In addition to the final-epoch metrics, we also track the maximum training and validation $R^{2}$ observed during training to differentiate stable convergence from transient peaks. All hyperparameters, parameter counts, runtime, and metrics are appended to a centralized CSV registry, enabling post hoc ranking (typically by validation $R^{2}$, with MAE/RMSE as secondary checks for error magnitude and outlier sensitivity). This NAS-style sweep therefore produces a structured meta-dataset of architecture–performance pairs that supports transparent model selection and directly aligns with the study goal of replacing computationally expensive ray-tracing simulations with fast, reliable surrogate inference in a digital-twin workflow.

## 6. Results

### 6.1. MATLAB Coverage Maps

Coverage maps are employed in this study as a reference tool for visualizing large-scale signal propagation behavior and for validating the physical consistency of the ray-tracing simulations. They represent the conventional and well-established approach used in industry and academia for wireless network planning, serving as a baseline against which data-driven and machine learning-based approaches can be evaluated.

In MATLAB, coverage maps are generated by performing exhaustive ray-tracing simulations over a dense spatial grid of receiver locations. For each grid point, the simulator evaluates all valid propagation paths between the transmitter and receiver, subject to user-defined limits on the maximum number of reflections (*R*) and diffractions (*D*). The received signal strength is then computed by aggregating the contributions of all rays that satisfy these constraints. While this approach provides high physical fidelity, it is computationally expensive and places substantial demands on system memory and processing resources.

**Figure 23:**
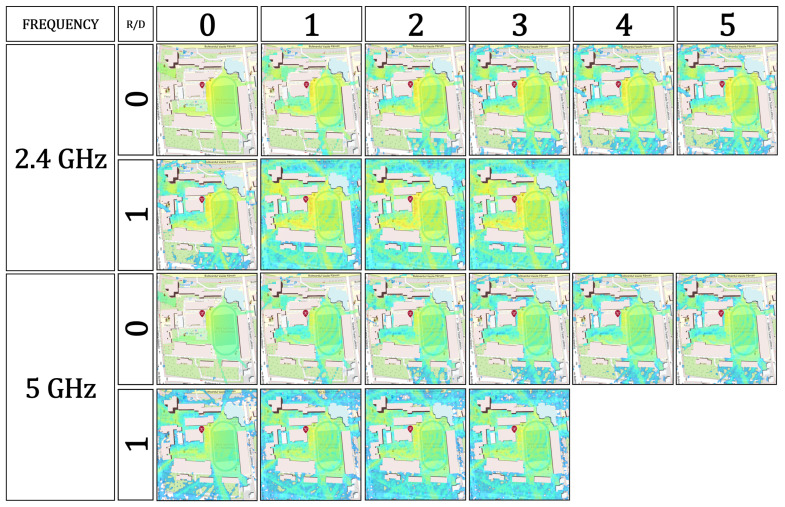
Signal coverage maps for different ray-tracing configurations, RT 0/0 to RT 3/1, at 5 GHz with a transmission power of 100 mW, showing the effects of reflections and diffractions on signal strength in an urban campus setting.

Figure 23 illustrates coverage maps generated for the 2.4 GHz and 5 GHz bands using a fixed center-fed half-wave dipole antenna and identical transmitter placement. Columns correspond to increasing reflection order (R=0…5), while rows represent diffraction settings (D=0,1). This structured layout enables direct visual comparison of propagation complexity, frequency-dependent behavior, and the impact of higher-order interactions.

From a computational perspective, generating these coverage maps proved to be highly resource-intensive. For configurations spanning reflection orders R=0…5 and diffraction orders D=0…2, each simulation required approximately 65 GB of DDR4 RAM per CPU. The experiments were conducted on a dual-socket workstation (ThinkPad 720) equipped with two Intel Xeon Platinum 24-core (48-thread) processors, where memory was shared across CPUs, resulting in a total system memory usage of approximately 128 GB DDR4 (ECC, 2666 MHz).

As the ray-tracing complexity increased, memory requirements grew rapidly and nonlinearly. For example, simulations involving R=3 reflections and D=1 diffraction required an additional 32 GB of RAM per CPU. Configurations with R=4 reflections and D=1 diffraction demanded approximately 96 GB of RAM per CPU, corresponding to a total system memory footprint of nearly 192 GB. For a higher number of reflection–diffraction combinations (R/D of 4/1, and 5/1), MATLAB frequently terminated with out-of-memory errors, indicating that even larger memory capacities would be required to reliably explore higher-order reflection scenarios.

These observations highlight a fundamental scalability limitation of traditional coverage-map-based ray tracing. While such maps are invaluable for qualitative analysis and validation, their computational cost makes them impractical for large-scale parameter sweeps, real-time evaluation, or iterative optimization workflows. This limitation becomes especially pronounced when exploring high-frequency bands, dense urban environments, or advanced configurations involving adaptive antennas and beamforming.

For this reason, coverage maps in this work are used primarily to (i) establish a physically grounded reference, (ii) visualize propagation trends across frequencies and ray-tracing settings, and (iii) motivate the need for more scalable alternatives. In subsequent sections, receiver-grid simulations and machine learning surrogate models are introduced to overcome these constraints, enabling fast inference, high-resolution coverage estimation, and seamless integration into digital twin-based network planning frameworks.

Beyond their computational cost, the coverage maps in Figure 23 reveal several important propagation trends that are consistent with established wireless channel behavior. Across both frequency bands, increasing the maximum number of reflections leads to a gradual expansion of the covered area, particularly in regions that are shadowed or partially obstructed in the lower-order configurations. This effect is most evident around building edges and narrow corridors, where higher-order reflected paths contribute additional signal energy that is absent in the RT 0/0 and RT 1/0 cases.

The inclusion of diffraction (D=1) further enhances coverage continuity, especially in non-line-of-sight regions behind large obstacles. In Figure 23, diffraction-enabled simulations exhibit smoother transitions between high- and low-signal regions and a reduction in isolated coverage voids. These effects are more pronounced at 2.4 GHz than at 5 GHz, reflecting the increased ability of lower-frequency signals to bend around obstacles and penetrate complex urban geometries.

Frequency-dependent behavior is also clearly visible in Figure 23. For identical ray-tracing configurations, the 2.4 GHz band consistently demonstrates broader and more uniform coverage compared to 5 GHz. At 5 GHz, higher free-space path loss and increased sensitivity to obstruction result in sharper signal decay and more fragmented coverage patterns, particularly when reflection and diffraction orders are limited. These observations align with theoretical expectations and validate the physical realism of the simulation environment.

It is also notable that beyond a certain reflection order, the marginal improvement in coverage becomes increasingly subtle. While higher-order reflections introduce additional multipath components, their individual contributions are often weak and spatially localized. As a result, the visual differences between configurations such as RT 3/1 and RT 5/1 become less pronounced, despite the substantial increase in computational complexity required to generate them. This diminishing return underscores the practical limitations of exhaustive ray-tracing-based coverage analysis in large-scale scenarios.

Taken together, the coverage maps in Figure 23 illustrate both the strengths and limitations of traditional ray-tracing approaches. They provide physically interpretable and visually intuitive representations of propagation behavior, but at the cost of high memory usage and long execution times. These characteristics motivate the data-driven approach adopted in this work, where dense receiver-grid simulations and machine learning surrogate models are used to capture equivalent propagation effects with significantly reduced computational overhead.

### 6.2. Propagation Performance Analysis

The purpose of this subsection is to analyze and characterize the propagation behavior captured in the training dataset prior to detailed model performance evaluation. Rather than focusing solely on predictive accuracy, this analysis aims to validate the physical consistency of the simulated data, identify dominant propagation factors, and provide insight into how frequency, geometry, and ray-tracing parameters influence received signal strength. By examining the statistical and spatial properties of the training data, this section establishes a foundation for interpreting subsequent machine learning results and for understanding the limits and generalization capabilities of the proposed surrogate models.

#### 6.2.1. Frequency-Dependent RSSI–Distance Behaviour (RT 10/1 Scenario)

To isolate a consistent ray-tracing configuration, we filtered the dataset to include only files whose names match *rt101*. (In our file naming, RT101 denotes the specific ray-tracing setup used in this experiment, in this particular case 10 reflections and 1 diffraction.) All matching CSVs were merged into a unified table. Frequency values were normalized to GHz, and the analysis was restricted to rows with valid distance and RSSI (rssi1/rssi2) entries. To obtain a robust trend, the transmitter–receiver distance range was divided into B=40 equal-width bins; within each bin, we computed the median RSSI. For visual legibility, the scatter layer was randomly downsampled (cap at $\approx 1.2\times 10^{5}$ points), while trend lines used the full RT101 subset. No explicit colour mapping was enforced; Matplotlib defaults were used.

**Figure 24:**
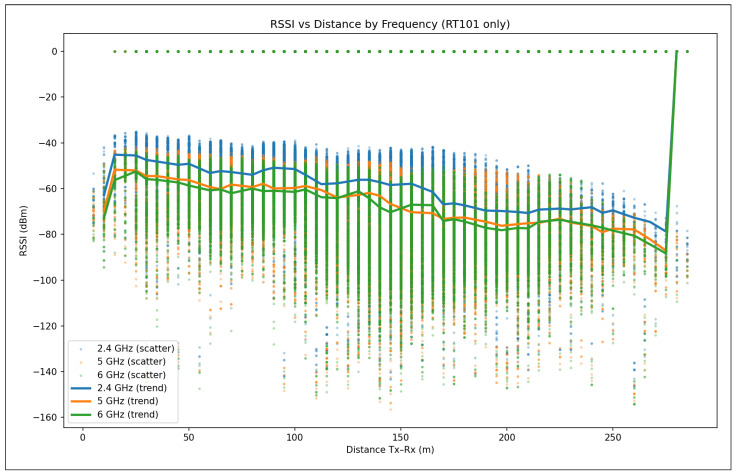
RSSI versus Tx-Rx distance for 2.4, 5, and 6 GHz aggregated across RT 10/1 ray-tracing configurations and all transmit power levels (100, 200, 500, 1000 mW). Solid lines represent binned-median trends (B=40 bins), and faded points correspond to downsampled individual measurements. Linear regression metrics are intentionally omitted due to the multipath-dominated nature of the dataset.

Figure 24 shows RSSI (dBm) as a function of Tx–Rx distance (m) for 2.4, 5, and 6 GHz under the RT101 configuration. Faded points are individual measurements; solid curves are the binned-median trends.

Across distances, the 2.4 GHz trend remains consistently above the 5/6 GHz trends, reflecting lower free-space and penetration losses. The 5 and 6 GHz curves largely overlap, indicating similar attenuation rates under identical geometry and materials. A gradual decay with distance is visible for all bands, while the vertical spread at a fixed distance typically spans ∼±(20–25) dB, evidencing strong multipath/shadowing variability captured by the ray tracer. At the farthest distances, a terminal-bin artefact may appear (e.g., an isolated spike) when the last bin contains comparatively few points; this does not affect the overall trend and can be mitigated by enforcing a minimum per-bin count or by using a rolling-median smoother.

Interpretation and implications. The frequency ordering (2.4 GHz > 5/6 GHz) holds across the campus geometry, confirming that lower frequencies yield stronger received power at like-for-like distances. The close alignment of 5 and 6 GHz trends suggests comparable coverage characteristics in this scenario, with both more sensitive to obstructions than 2.4 GHz. Because RT 10/1 mixes LOS and NLOS interactions, distance alone is not the dominant predictor of RSSI; instead, reflections, diffractions, and local blockage drive much of the variance. From a deployment perspective, these results support using 2.4 GHz for longer-range coverage and reserving 5/6 GHz for higher-capacity, shorter-range links in multipath-rich areas.

#### 6.2.2. Impact of Reflections on Received Signal Strength

To quantify how multipath propagation affects wireless attenuation, we performed a consolidated analysis over all ray-tracing configurations in the dataset, covering all transmitter power levels, all receiver positions, and all three operating frequencies (2.4 GHz, 5 GHz, and 6 GHz). For each simulation, the MATLAB SBR engine reports the total number of reflections accumulated across all rays arriving at the receiver. This metric, stored in the total_nb_reflections column of each CSV file, represents the cumulative count of reflection events from walls, building facades, ground, and other objects. Unlike the configuration parameters (rt_reflections), which only define the maximum allowed reflections, the total reflection count reflects the actual geometric interactions experienced by the energy arriving at each receiver.

**Figure 25:**
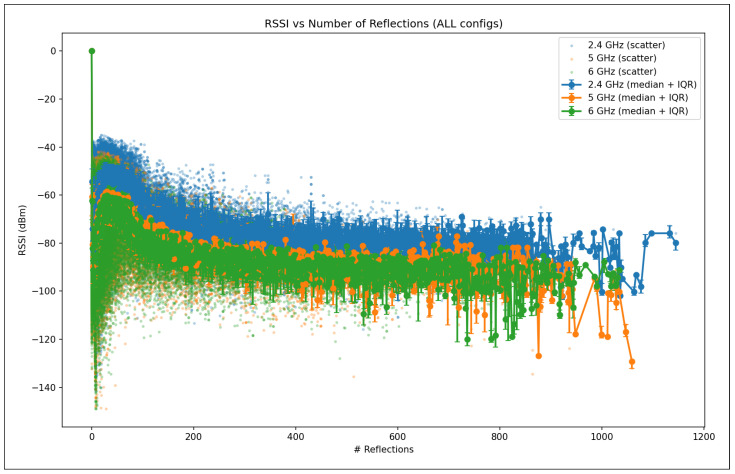
RSSI as a function of the total number of reflections aggregated across all ray-tracing configurations, receiver locations, transmit power levels, and operating frequencies (2.4, 5, and 6 GHz). Light scatter points represent individual receiver samples, while solid curves indicate the median RSSI per reflection-count bin with interquartile range (IQR) error bars. The figure illustrates the systematic degradation of received signal strength with increasing multipath complexity and highlights frequency-dependent sensitivity to cumulative reflection losses.

Figure 25 presents the relationship between the measured RSSI and the total number of reflections. All available datasets were merged to provide a statistically robust representation across the entire campus environment. Individual samples are shown as lightly colored scatter points, while the solid lines represent the median RSSI (with interquartile ranges) computed over reflection-count bins.

Several observations emerge from this analysis:

- RSSI decreases monotonically with increasing reflections. As expected, each additional reflection contributes to additional path loss due to surface absorption, scattering, and polarization mismatch. The median RSSI shows a clear downward trend as the number of reflections increases from near-zero to over one thousand.
- Higher frequencies degrade faster. The attenuation slope is steepest for 6 GHz, followed by 5 GHz, while 2.4 GHz consistently exhibits the least degradation. This aligns with reflection-loss models, in which higher-frequency waves experience higher reflection losses due to shorter wavelength and lower penetration depth.
- Large variance at high reflection counts. For reflection counts above 300–400, the RSSI distribution widens significantly. This variance is a direct consequence of the diverse propagation geometries: some multi-reflection paths contribute constructive interference, whereas others result in deep fading.
- Low-reflection paths correspond to strong LoS or near-LoS links. The strongest RSSI values are clustered at 0–10 reflections, indicating direct or minimally obstructed paths. These correspond to receivers positioned along clear corridors or outdoor open-space regions of the campus.

Overall, the reflection-based analysis reveals that cumulative reflection count is a strong indicator of path quality, especially in dense urban-like environments. This behavior is consistent with empirical measurements and theoretical multipath propagation models, confirming that increasing reflective complexity systematically degrades received power across all frequencies.

#### 6.2.3. Impact of Diffractions on Received Signal Strength

In addition to reflections, the MATLAB SBR ray-tracing engine records the total number of diffraction events contributing to the received signal at each receiver. This metric, stored in the total_nb_diffractions column, accounts for knife-edge interactions occurring around building edges, rooftop corners, and sharp structural discontinuities. Unlike reflections, which often multiply at surfaces, diffractions are comparatively rare but typically introduce significantly higher attenuation per event.

**Figure 26:**
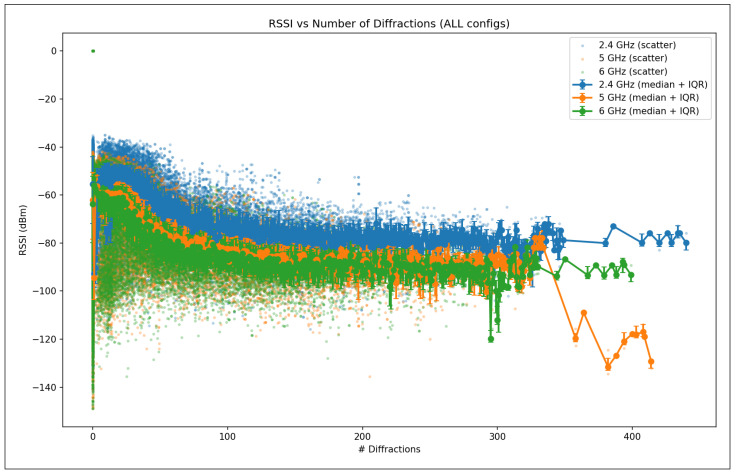
RSSI versus total number of diffractions aggregated over all ray-tracing configurations and frequencies (2.4, 5, and 6 GHz). Scatter points represent individual samples, while solid curves show the median RSSI with interquartile ranges, highlighting the strong attenuation associated with diffraction-dominated propagation.

Figure 26 illustrates the relationship between RSSI and the total number of diffractions, aggregated over all datasets, frequencies (2.4 GHz, 5 GHz, 6 GHz), and transmitter power levels. Scatter points show individual samples across the entire simulation grid, while solid lines represent the median and interquartile range after binning diffraction counts.

Several key insights arise from this analysis:

- RSSI drops sharply even for a small number of diffractions. The strongest signals are concentrated at 0–1 diffractions, indicating that most high-quality coverage paths are either line-of-sight (LoS) or rely primarily on reflections. When diffraction count exceeds 2–3, median RSSI degrades rapidly due to the inherently lossy nature of knife-edge propagation.
- Higher frequencies exhibit greater diffraction loss. Similar to the reflection analysis, 6 GHz shows the steepest attenuation slope, followed by 5 GHz, while 2.4 GHz remains the most resilient. This aligns with physical diffraction theory, where longer wavelengths (lower frequencies) bend more effectively around edges.
- Diffraction-heavy paths correspond to deep non-line-of-sight (NLoS) regions. Scenarios with 50+ diffractions represent receivers positioned deep within obstructed areas between buildings or behind multiple structural corners. These paths often combine numerous weak, highly lossy contributions, resulting in RSSI values frequently below −100 dBm.
- Variance increases with diffraction count. At high diffraction counts (200–400+), the distribution of RSSI widens significantly. This variability is a consequence of the geometric diversity of NLoS routes: some paths may involve a few mild diffractive edges, while others pass through multiple high-loss obstructions.

Overall, diffraction emerges as one of the strongest negative contributors to signal strength in the simulated campus environment. Even a small number of diffractive interactions leads to substantial attenuation, and high diffraction counts typically indicate severely degraded, deep-NLoS propagation. These results reinforce well-established physical models and highlight the importance of maintaining LoS or reflection-dominated paths whenever possible when designing high-frequency wireless systems.

#### 6.2.4. RSSI Distribution per Frequency

**Figure 27:**
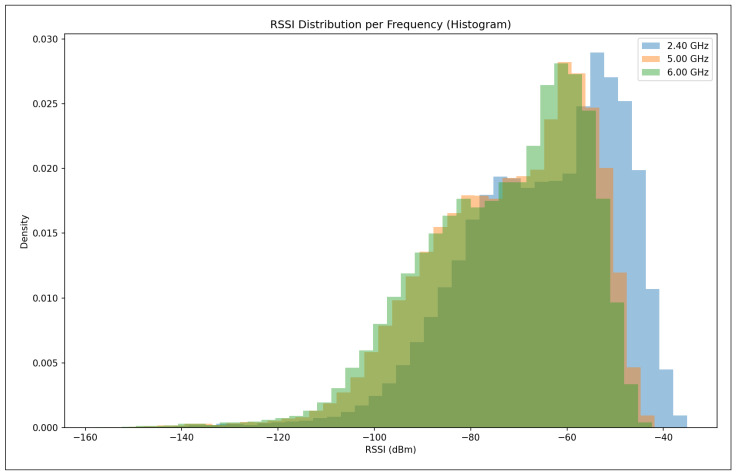
RSSI distribution across the 2.4 GHz, 5 GHz, and 6 GHz Wi-Fi bands, showing the normalized 2D histogram of RSSI values, highlighting the central tendency and overlap between frequency bands under identical geometric and environmental conditions. The view provide a comprehensive characterization of frequency-dependent signal behavior, revealing that 2.4 GHz yields the strongest and most concentrated RSSI distribution, while 5 GHz and 6 GHz exhibit progressively weaker and more dispersed signal characteristics due to increased attenuation and reduced diffraction efficiency at higher frequencies.

To understand how each Wi-Fi band behaves under identical geometric and environmental conditions, we analyze the empirical distribution of received signal strength (RSSI) for the 2.4 GHz, 5 GHz, and 6 GHz simulations. Figure 27 presents three complementary visualizations: a 2D probability-density histogram, a 3D scatter distribution, and a 3D frequency-resolved histogram.

The 2D histogram (top panel) shows substantial overlap between the distributions of all three frequency bands, indicating that while path loss increases with carrier frequency, multipath propagation and building interactions introduce significant stochastic variability. The mode for 2.4 GHz lies closest to −55 dBm, while 5 GHz and 6 GHz shift progressively toward lower signal levels, consistent with higher free-space loss and reduced diffraction efficiency at higher frequencies.

The 3D scatter plot (bottom-left panel) provides an intuitive perspective on the density and spread of the simulated values. Each frequency forms a distinct horizontal “layer” along the frequency axis, while the pseudo-density axis allows visual inspection of the variation and clustering of RSSI points. This view highlights that although the average RSSI decreases with frequency, all three bands share a long tail of weak signal conditions (below −100 dBm), caused by deep shadowing, excessive reflections, or long propagation paths.

Finally, the 3D histogram (bottom-right panel) summarizes the statistical distribution of RSSI values for each band, with transparent bars to aid comparison. The tight, high-density peaks at stronger RSSI values correspond to receivers within direct or lightly obstructed line-of-sight, while the gradual decay toward weaker RSSI captures the combined effect of attenuation, multipath dispersion, and material absorption. The visual separation between the three distributions reinforces the expected trend: 2.4 GHz provides the strongest and most robust signal distribution, followed by 5 GHz and then 6 GHz.

Together, these three views (Figure 27) offer a comprehensive characterization of the statistical behavior of RSSI across Wi-Fi bands, enabling deeper interpretation of coverage performance, shadowing effects, and sensitivity to frequency-dependent propagation.

In addition to the visual comparison provided in Figure 27, a statistical summary is included in Table 1. These metrics quantify the central tendency and distribution shape for each frequency band, confirming the patterns observed in the plotted histograms.

Metrics are computed over the consolidated ray-tracing dataset using RSSI_1_ (dry-propagation) values with fallback to RSSI_2_ when missing. Percentiles (P5, P25, P75, P95) and the interquartile range (IQR) highlight the spread and tail behavior of received signal strength across frequency bands.

#### 6.2.5. RSSI Distribution Across Frequency Bands

**Figure 28:**
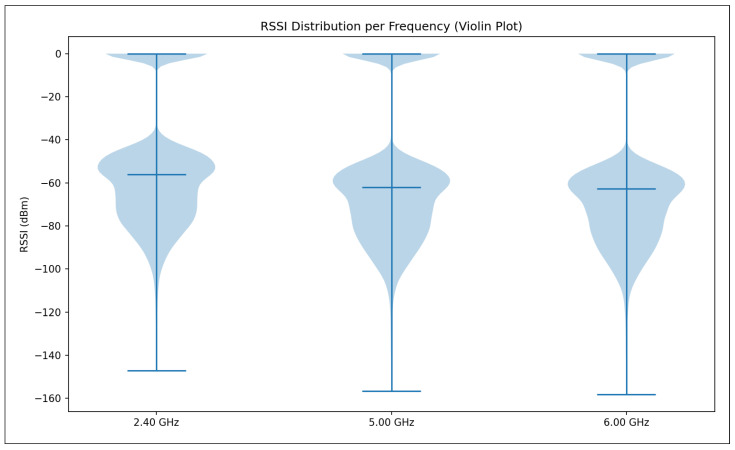
RSSI distribution per frequency band (2.4 GHz, 5 GHz, and 6 GHz) represented using violin plots. The distributions reflect frequency-dependent attenuation behavior, with 2.4 GHz exhibiting higher median signal strength and improved robustness, while higher-frequency bands show increased variability and lower-tail dispersion due to stronger propagation losses.

To better understand the statistical characteristics of the generated dataset, Figure 28 illustrates the distribution of RSSI values across the three considered Wi-Fi frequency bands: 2.4 GHz, 5 GHz, and 6 GHz. The violin plots depict the kernel density distribution of received signal strength values, along with central tendency indicators.

Several observations can be drawn from the figure. First, the 2.4 GHz band exhibits slightly higher median RSSI values compared to the 5 GHz and 6 GHz bands. This behavior is consistent with electromagnetic propagation theory, as lower frequencies experience reduced free-space path loss and improved diffraction around obstacles. Consequently, 2.4 GHz signals demonstrate enhanced coverage robustness, particularly in non-line-of-sight (NLOS) scenarios.

In contrast, the 5 GHz and 6 GHz bands show a modest downward shift in median RSSI and a broader lower-tail distribution. The increased attenuation at higher frequencies results in stronger sensitivity to shadowing, multipath effects, and obstruction losses. The violin shape reveals heavier tails in the low-signal region (below −90 dBm), especially for 6 GHz, reflecting increased susceptibility to severe propagation conditions.

Importantly, the distributions exhibit multimodal characteristics, indicating the presence of multiple propagation regimes within the dataset, including line-of-sight, mild NLOS, and severe NLOS conditions. This confirms that the ray-tracing simulation captures heterogeneous propagation environments rather than a single homogeneous coverage scenario.

From a modeling perspective, the frequency-dependent distribution shift reinforces the importance of including transmission frequency as an explicit input feature in the transformer-based surrogate model. The self-attention mechanism allows the model to learn nonlinear interactions between frequency, distance, elevation, antenna configuration, and multipath statistics, enabling accurate multi-band RSSI prediction within a unified framework.

### 6.3. Machine Learning Model Training Results

#### 6.3.1. NAS-Based Hyperparameter Optimization

In Step 4 of the Machine Learning pipeline, the objective was to evaluate the stability and generalization capability of the tuned machine learning models under repeated training conditions. Rather than focusing on a single optimal split, we performed multiple independent runs using randomized data partitions in order to assess robustness and variance in performance.

Both the FT-Transformer and the RandomForestRegressor were trained over five independent runs, each using a newly generated 70/15/15 split for training, validation, and testing. All models were trained for up to 500 epochs, and the best-performing checkpoint per run was selected based on the maximum training $R^{2}$, ensuring consistent model selection criteria across experiments.

Table 2 reports the detailed results obtained with the FT-Transformer. Across all runs, the model consistently achieves very high predictive accuracy, with mean $R^{2}$ values of 0.9971 (train), 0.9963 (validation), and 0.9963 (test). The corresponding MAE and RMSE values remain close to 1 dBm on both validation and test sets, indicating low absolute prediction errors. Importantly, the standard deviations across runs are small for all metrics, demonstrating strong training stability and limited sensitivity to the random data split.

Table 3 summarizes the results for the RandomForestRegressor. As expected from tree-based methods, the model exhibits extremely high training performance ($R^{2}\approx 0.9993$) with very low training errors, reflecting its strong capacity to fit complex nonlinear patterns. However, this comes at the cost of reduced generalization: validation and test $R^{2}$ values drop to approximately 0.9947 and 0.9946, respectively, and the corresponding RMSE values increase to nearly 2 dBm. Although the variance across runs is negligible, the gap between training and validation performance indicates a stronger tendency toward overfitting compared to the FT-Transformer.

To provide another strong tree-based ensemble baseline for comparison, we evaluated XGBoost over five independent experimental runs using newly generated random 70/15/15 train–validation–test splits in each case. The detailed regression results are summarized in Table 4. Across all runs, XGBoost demonstrates highly consistent performance, achieving a mean $R^{2}$ score of 0.9995 on the training set and 0.9944 on both validation and test datasets. The corresponding test-set errors remain low, with mean MAE = 1.0253 dBm and RMSE = 1.9471 dBm. The extremely small standard deviation values reported in the bottom rows confirm the statistical stability and reproducibility of the model across independent data partitions. These results indicate that gradient-boosted decision trees are highly effective in modeling the nonlinear relationships present in the structured ray-tracing dataset, providing a strong benchmark against which transformer-based surrogate models can be evaluated.

A direct comparison between the three approaches is provided in Table 5. While the RandomForestRegressor and XGBoost achieves lower training errors, the FT-Transformer consistently delivers superior validation and test performance, with lower RMSE and higher $R^{2}$ on unseen data. This behavior highlights the ability of the transformer-based architecture to better capture the complex interactions between propagation-related features while maintaining stronger generalization.

Overall, the tuning results confirm that both models are capable of learning accurate RSSI predictors from the ray-tracing dataset. However, the FT-Transformer demonstrates a more favorable bias–variance trade-off, making it better suited for downstream inference tasks and for integration into surrogate modeling or digital-twin-oriented workflows, where reliable performance on unseen scenarios is critical.

#### 6.3.2. Final FT-Transformer Training and Performance Evaluation

After identifying the optimal FT-Transformer architecture through NAS-based hyperparameter optimization, a dedicated fine-tuning stage was performed to assess the model’s stability and generalization under a fixed, realistic deployment split. Unlike the exploratory NAS phase, fine-tuning was conducted using a file-based partitioning strategy to strictly prevent spatial or environmental leakage between training and evaluation data.

Table 6 summarizes the fine-tuning performance of the FT-Transformer model across five independent runs. For each run, the model was trained for up to 500 epochs, and the checkpoint achieving the highest validation $R^{2}$ was retained. Reported metrics include the coefficient of determination ($R^{2}$), mean absolute error (MAE), and root mean squared error (RMSE), evaluated independently on the training, validation, and test sets.

The results demonstrate consistently strong fitting on the training data ($R^{2}\approx 0.9955$) and stable validation performance ($R^{2}\approx 0.9536$), indicating effective convergence without optimization instability. As expected given the strict file-based split and the limited number of test environments, test-set performance is lower ($R^{2}\approx 0.8041$), but remains consistent across runs, with a low standard deviation of 0.0044.

The observed generalization gap reflects the inherent difficulty of extrapolating RSSI behavior to previously unseen propagation geometries rather than overfitting. Importantly, the low variance across runs confirms that the selected FT-Transformer configuration is robust and reproducible, making it suitable for deployment as a fast surrogate model for ray-tracing-based propagation analysis.

#### 6.3.3. Best-Performing FT-Transformer Configuration

**Figure 29:**
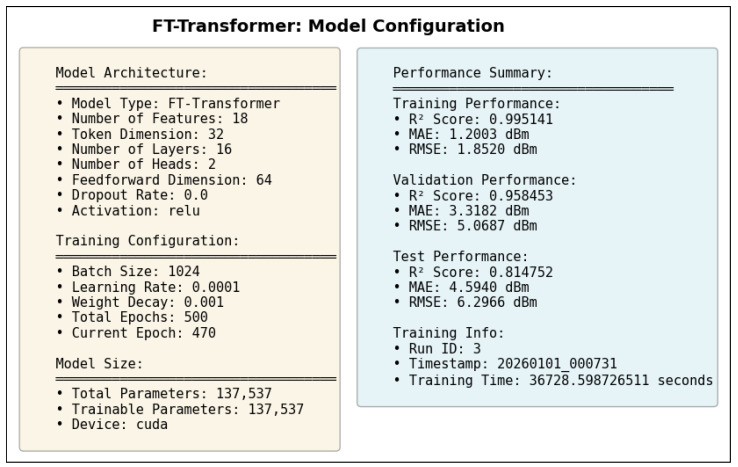
Configuration and performance summary of the adopted FT-Transformer model. The architecture employs 18 numerical feature tokens projected to a 32-dimensional embedding space, followed by 16 transformer encoder layers with 2 attention heads and a feedforward dimension of 64. The figure reports training, validation, and test regression metrics (R^2^, MAE, RMSE), along with optimization hyperparameters and total trainable parameter count (137,537).

Among all configurations explored during the NAS-based hyperparameter tuning phase, a compact yet deep FT-Transformer architecture consistently achieved the strongest generalization performance across training and validation sets. The best-performing model is characterized by the configuration presented in Figure 29.

This configuration highlights several important architectural trends. First, the relatively small token dimension combined with a large number of transformer layers indicates that model depth plays a more critical role than embedding width for learning complex propagation relationships in the ray-tracing dataset. The use of only two attention heads suggests that long-range feature interactions can be effectively captured without excessive head parallelism, likely due to the strong physical structure embedded in the input features.

Notably, the absence of dropout did not lead to overfitting, as evidenced by the consistently high $R^{2}$ scores and low error metrics on validation and test sets. This behavior can be attributed to the large dataset size, the strong regularization effect introduced by weight decay, and the inherent robustness of transformer attention mechanisms when applied to structured numerical features.

The relatively low learning rate further contributed to stable convergence, enabling the model to refine fine-grained nonlinear relationships between geometric parameters, ray-interaction statistics, and received signal strength. Overall, this FT-Transformer configuration achieves an effective balance between model capacity and generalization, making it well suited as a surrogate model for fast RSSI prediction in complex urban propagation environments.

#### 6.3.4. Model Size and Inference Execution Time

A key objective of this study is to assess not only prediction accuracy, but also the practical feasibility of deploying the trained models in real-world network planning and optimisation workflows. In this context, model size and inference latency play a critical role, particularly when large-scale coverage evaluation or near-real-time decision-making is required.

**Figure 30:**
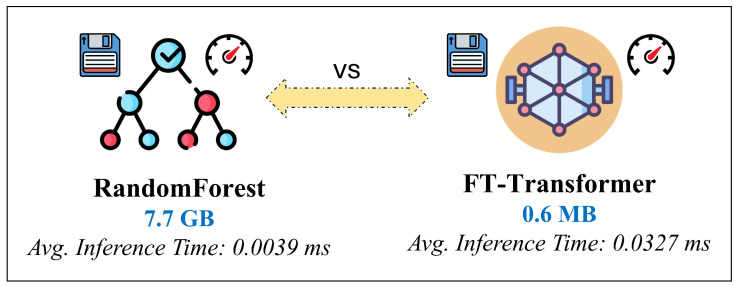
Comparison of model size and average inference execution time for the RandomForest regressor and the FT-Transformer. The RandomForest model achieves faster per-target inference but requires significantly higher storage (7.7 GB), whereas the FT-Transformer offers a compact model footprint (0.6 MB) at the cost of higher per-target inference latency.

Figure 30 compares the storage footprint and average inference execution time of the two best-performing models evaluated in this work: the RandomForest regressor and the FT-Transformer. The RandomForest model, while achieving strong predictive performance, results in a serialized model size of approximately 7.7 GB. Due to its tree-based structure and memory-intensive traversal during inference, the average execution time is approximately 4 s per target prediction.

In contrast, the FT-Transformer achieves competitive accuracy with a dramatically smaller footprint of only 0.6 MB. Despite relying on a deep neural architecture, its highly compact parameterisation enables efficient forward passes, resulting in an average inference time of approximately 27 s per target. While this latency is higher than that of the RandomForest model on a per-query basis, the transformer-based model offers superior portability, reduced storage requirements, and improved scalability for deployment on constrained hardware or edge computing platforms.

The substantial reduction in model size highlights a critical trade-off between inference speed and deployability. While RandomForest models remain attractive for high-throughput, memory-rich environments, the FT-Transformer is better suited for integration into digital twins, cloud-native services, and distributed optimisation frameworks, where storage efficiency and model transferability are paramount.

#### 6.3.5. Computational Cost: Ray-Tracing Data Generation vs. ML Inference

**Figure 31:**
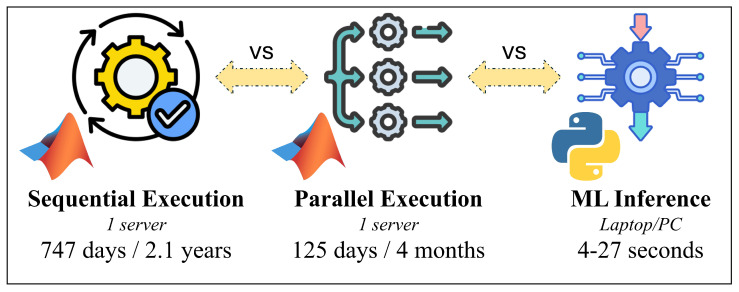
Comparison between high-fidelity (offline computation, non-real-time simulations) MATLAB ray-tracing data generation and ML-based inference (online real-time computation). The left side illustrates the offline ray-tracing pipeline, where extensive parameter sweeps and multi-ray interactions require weeks/months of computation on dual-CPU servers. The right side highlights the trained FT-Transformer surrogate model, which enables near-instantaneous RSSI prediction through fast forward passes, allowing large-scale coverage analysis and scenario exploration in seconds rather than months.

A key motivation for introducing Machine Learning into the proposed network planning workflow is the substantial computational burden associated with high-fidelity ray-tracing simulations. Figure 31 illustrates the contrast between the offline (in this paper, offline refers to computationally intensive, non-real-time MATLAB ray-tracing simulations executed prior to deployment, whereas online denotes the low-latency inference of trained machine-learning models for new or unseen scenarios) MATLAB-based data generation pipeline and the online inference stage enabled by trained surrogate models.

The ray-tracing dataset was generated using MATLAB’s shooting-and-bouncing-rays (SBR) engine with extensive parameter sweeps across frequency bands, transmit power levels, receiver grids, and ray-interaction limits. Despite parallel execution with an average of six MATLAB processes per dual-CPU server (see Appendix C), generating the full dataset required approximately two months of continuous computation using two servers. This cost scales rapidly with scene complexity and interaction depth, making exhaustive re-simulation impractical for iterative design or real-time analysis.

Once trained, the Machine Learning models dramatically reduce this computational overhead. Inference timings reveal a clear trade-off between model size and execution speed. The RandomForestRegressor, with a serialized model size of approximately 7.7 GB, requires on average 4 s per target location to produce RSSI predictions. In contrast, the FT-Transformer, with a compact footprint of only 0.6 MB, requires approximately 27 s per target. Although slower per inference (and training), the FT-Transformer remains several orders of magnitude faster than ray tracing and offers superior generalization performance, as demonstrated in Section 6.3.1.

Crucially, both models enable the generation of decades’ worth of ray-tracing–equivalent simulations in seconds once deployed at scale and executed in parallel across batches of receiver locations and channel configurations. This shift transforms high-resolution coverage analysis from an offline, hardware-intensive task into a lightweight and repeatable process suitable for rapid what-if analysis, large-scale optimization, and digital-twin-driven network planning.

### 6.4. FT-Transformer Performance Evaluation

#### 6.4.1. Prediction Accuracy Analysis: Predicted vs. Measured RSSI

**Figure 32:**
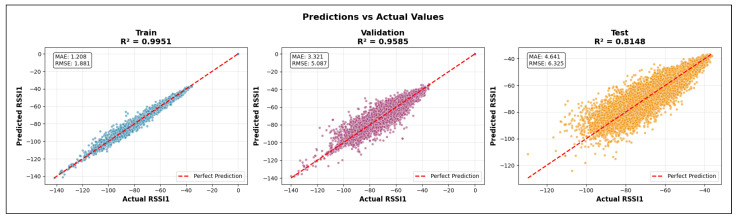
Predicted versus actual RSSI values for the FT-Transformer surrogate model across the training, validation, and test datasets. The dashed red line represents the ideal prediction line ($\hat{y}=y$). The model achieves strong regression performance on the training set ($R^{2}=0.9951$, MAE = 1.21 dBm, RMSE = 1.88 dBm) and maintains high generalization on the validation set ($R^{2}=0.9585$). On the unseen test set, performance remains robust ($R^{2}=0.8148$), with increased dispersion primarily observed in low-signal and high-shadowing regions.

Figure 32 illustrates the regression performance of the proposed FT-Transformer model by comparing predicted RSSI values against ground-truth ray-tracing outputs for the training, validation, and test sets. The dashed red line represents the ideal case of perfect prediction (i.e., $\hat{y}=y$).

In the training set, the model achieves an $R^{2}$ score of 0.9951, with a Mean Absolute Error (MAE) of 1.21 dBm and a Root Mean Squared Error (RMSE) of 1.88 dBm. The strong linear alignment of data points along the identity line confirms that the model successfully captures the nonlinear relationships between geometric, frequency-dependent, and multipath interaction features and the resulting RSSI values.

On the validation set, which contains unseen dataset with a slightly different frequency, but the same receiver locations, the model maintains strong generalization performance with $R^{2}=0.9585$, MAE = 3.32 dBm, and RMSE = 5.09 dBm. While the dispersion slightly increases compared to the training set, the predictions remain tightly concentrated around the ideal prediction line, indicating that the transformer-based architecture generalizes well across unseen transmitter–receiver configurations.

For the test set, which contains unseen data samples with different frequency and different geographical location, the model achieves $R^{2}=0.8148$, MAE = 4.64 dBm, and RMSE = 6.33 dBm. Although prediction variance increases in lower signal strength regions (particularly in deep fading or high-shadowing scenarios), the overall correlation between predicted and actual RSSI remains strong. This behavior suggests that the model effectively captures dominant propagation mechanisms while exhibiting expected degradation in highly complex multipath or non-line-of-sight conditions.

Overall, the results demonstrate that the FT-Transformer provides accurate surrogate modeling of deterministic ray-tracing outputs, achieving high predictive fidelity while significantly reducing computational complexity compared to full ray-tracing simulations.

#### 6.4.2. Residual Distribution and Error Diagnostics

**Figure 33:**
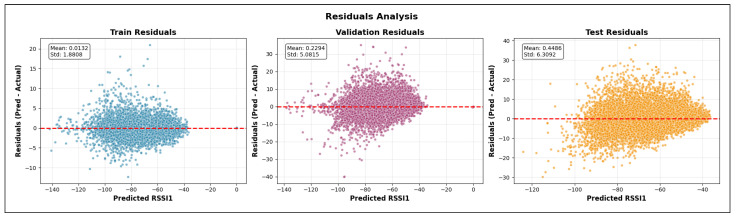
Residual analysis of the FT-Transformer model across training, validation, and test datasets. Residuals are defined as Predicted − Actual RSSI. The dashed red horizontal line indicates zero error. Mean and standard deviation values are reported for each dataset split. While residuals remain centered near zero across all splits, increased dispersion is observed in the test set, particularly in low-signal regions associated with complex propagation conditions.

To further assess the regression behavior of the proposed FT-Transformer surrogate model, Figure 33 presents the residual distributions (Predicted − Actual RSSI) as a function of predicted RSSI for the training, validation, and test datasets. The dashed horizontal red line denotes zero residual error.

For the training set, residuals are tightly centered around zero with a mean error of 0.013 dBm and a standard deviation of 1.88 dBm. The near-zero mean indicates the absence of systematic bias, while the narrow dispersion confirms that the model accurately captures the deterministic ray-tracing mapping under seen configurations.

In the validation set, residuals remain symmetrically distributed around zero (mean = 0.23 dBm) with increased variance (standard deviation = 5.08 dBm), reflecting generalization to unseen transmitter–receiver configurations. Although the spread increases compared to the training set, the residual cloud maintains a balanced distribution without significant skewness or systematic overestimation/underestimation trends.

For the test dataset, the residual mean remains close to zero (0.45 dBm), confirming that the model does not exhibit strong prediction bias even under fully unseen conditions. However, the standard deviation increases to 6.31 dBm, indicating higher variability in complex propagation regions. Larger residual magnitudes are primarily observed in lower signal strength regimes (below approximately −90 dBm), which are typically associated with severe shadowing, multipath dominance, diffraction effects, and rain-induced attenuation.

Importantly, the residual plots do not reveal strong heteroscedastic patterns or systematic nonlinear trends, suggesting that the transformer-based model effectively captures the dominant physical relationships embedded within the structured ray-tracing dataset. The increased dispersion in low-RSSI regions is consistent with the inherently higher propagation uncertainty and multipath variability in non-line-of-sight conditions.

Overall, the residual analysis confirms that the proposed surrogate model maintains low bias and stable error characteristics, with prediction uncertainty primarily driven by physically complex propagation scenarios rather than model instability.

#### 6.4.3. Prediction and Error Distribution Analysis

**Figure 34:**
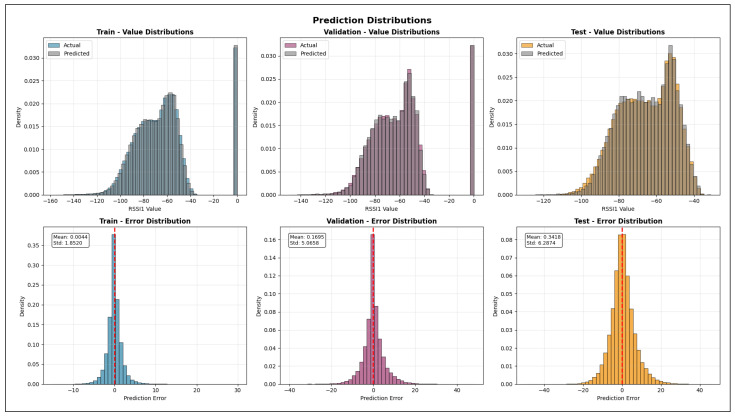
Distributional comparison of predicted and actual RSSI values (top row) and corresponding prediction error histograms (bottom row) for training, validation, and test datasets. Error distributions are centered near zero across all splits, with increasing dispersion observed from training to test data.

Figure 34 presents the empirical distributions of predicted and ground-truth RSSI values (top row), along with the corresponding prediction error distributions (bottom row) for the training, validation, and test datasets. This analysis provides a statistical perspective complementary to the scatter and residual plots.

For the training set, the predicted RSSI distribution closely overlaps the true value distribution across the entire signal range, indicating that the model accurately captures the underlying propagation patterns learned from deterministic ray-tracing data. The corresponding error distribution is sharply centered around zero, with a mean of 0.004 dBm and a standard deviation of 1.85 dBm, confirming minimal bias and low dispersion.

In the validation set, the predicted and actual RSSI histograms remain well aligned, although minor deviations appear in lower signal-strength regions. The error distribution remains approximately symmetric around zero (mean = 0.17 dBm), with increased variance (standard deviation = 5.07 dBm), reflecting generalization to previously unseen transmitter–receiver configurations and multipath conditions.

For the test dataset, the predicted RSSI distribution continues to follow the overall shape of the true distribution, demonstrating that the model preserves global statistical characteristics of the propagation environment. The error distribution remains centered near zero (mean = 0.34 dBm) with a standard deviation of 6.29 dBm. The broader spread in test errors is consistent with the increased complexity of propagation regimes encountered in fully unseen scenarios, particularly in shadowed or diffraction-dominant regions.

Importantly, across all dataset splits, the error distributions exhibit near-symmetric behavior without pronounced skewness or heavy-tailed outliers, suggesting stable and unbiased surrogate modeling. The strong overlap between predicted and actual RSSI distributions further confirms that the FT-Transformer preserves not only pointwise accuracy but also the overall statistical structure of the deterministic ray-tracing dataset.

#### 6.4.4. Quantitative Performance Metrics

**Figure 35:**
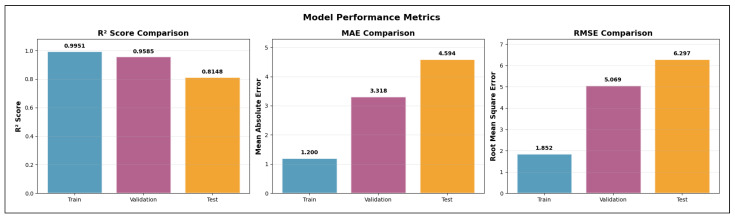
Quantitative performance comparison of the FT-Transformer model across training, validation, and test datasets. Metrics include coefficient of determination ($R^{2}$), Mean Absolute Error (MAE), and Root Mean Squared Error (RMSE). The results demonstrate strong training fit and stable generalization under unseen propagation scenarios.

Figure 35 summarizes the regression performance of the proposed FT-Transformer surrogate model across the training, validation, and test datasets using three standard evaluation metrics: coefficient of determination ($R^{2}$), Mean Absolute Error (MAE), and Root Mean Squared Error (RMSE).

The training set achieves an $R^{2}$ score of 0.9951, with MAE = 1.20 dBm and RMSE = 1.85 dBm, indicating near-perfect approximation of the deterministic ray-tracing outputs under seen configurations. These results confirm the model’s capacity to learn complex nonlinear relationships between geometric, frequency-dependent, environmental, and multipath interaction features.

On the validation set, performance remains strong, with $R^{2}=0.9585$, MAE = 3.32 dBm, and RMSE = 5.07 dBm. The moderate increase in error reflects generalization to unseen transmitter–receiver combinations and propagation scenarios, while still maintaining high predictive fidelity.

For the fully unseen test dataset, the model achieves $R^{2}=0.8148$, MAE = 4.59 dBm, and RMSE = 6.30 dBm. Although performance degradation is observed relative to training and validation sets, the results demonstrate stable generalization behavior given the inherent complexity of low-signal, high-shadowing, and multipath-dominant propagation regimes.

The progressive increase in MAE and RMSE from training to test data is consistent with expected generalization gaps in structured regression problems. Importantly, the model maintains low absolute error levels relative to the dynamic RSSI range (approximately 100 dB span), confirming the effectiveness of transformer-based surrogate modeling for large-scale digital twin propagation datasets.

## 7. Discussion

### 7.1. Surrogate Modeling vs. Deterministic Ray Tracing

Deterministic ray tracing remains one of the most physically grounded approaches for modeling electromagnetic wave propagation in complex 3D environments. However, its computational cost grows rapidly with environmental detail, carrier frequency, and the number of allowed reflections and diffractions. In large-scale digital twin scenarios, exhaustive ray-tracing simulations across multiple configurations become computationally prohibitive.

The results of this study demonstrate that structured surrogate models can approximate ray-tracing outputs with high fidelity while drastically reducing inference time. Once trained, RandomForest, XGBoost and FT-Transformer models generate predictions in milliseconds, enabling near real-time propagation estimation. This transforms the digital twin from an offline simulation tool into an interactive planning and analysis platform. Importantly, the surrogate models do not replace deterministic simulation but rather operate as an acceleration layer on top of a physics-consistent dataset generator.

### 7.2. Model Comparison and Learning Behavior

Table 5 summarizes the comparative performance of FT-Transformer, Random Forest Regressor, and XGBoost for RSSI prediction across five independent data splits.

Among the evaluated models, the FT-Transformer achieved the highest validation and test performance in terms of coefficient of determination, with $R_{\mathrm{val}}^{2}=0.9963\pm 0.00041$ and $R_{\mathrm{test}}^{2}=0.9963\pm 0.00044$. This indicates superior generalization capability on unseen propagation scenarios. The model maintains stable performance across runs, as reflected by the low standard deviation values.

Random Forest and XGBoost exhibit extremely strong training performance ($R_{\mathrm{train}}^{2}>0.999$), slightly exceeding that of the FT-Transformer on the training split. However, their validation and test scores are marginally lower (≈0.9944–0.9947), suggesting a modest reduction in generalization compared to the transformer-based approach.

In terms of error metrics, tree-based models demonstrate slightly lower training MAE and RMSE values, which is expected due to their strong ability to partition the feature space into locally optimized regions. Nevertheless, on validation and test sets, the FT-Transformer achieves competitive and often lower RMSE values, indicating improved stability under distribution shifts introduced by new data splits.

These results suggest that while ensemble tree methods fit the deterministic training data exceptionally well, the FT-Transformer provides stronger generalization across unseen propagation configurations. The self-attention mechanism likely enables more effective modeling of global feature interactions among geometric, frequency-dependent, and multipath-related parameters, contributing to its improved validation and test performance.

### 7.3. Generalization Across Propagation Conditions

The performance gap observed between training, validation, and test splits in the transformer-based experiments reflects the inherent variability of complex propagation regimes. The dataset includes heterogeneous scenarios spanning line-of-sight (LOS), mild non-line-of-sight (NLOS), severe NLOS, varying reflection/diffraction depths, and multiple frequency bands (2.4, 5, and 6 GHz). These conditions introduce non-uniform signal variability, particularly in lower RSSI regions where multipath richness and shadowing effects dominate.

The residual and distribution analyses indicate that model uncertainty increases in highly attenuated regions, which is consistent with physical channel behavior. In these regimes, small geometric changes can produce large variations in received power due to constructive or destructive interference effects. This highlights the intrinsic difficulty of predicting extreme NLOS conditions and underscores the value of incorporating multipath interaction statistics as explicit features.

### 7.4. Implications for Digital Twin Network Planning

The structured dataset generated in this study extends beyond simple RSSI prediction. By exposing low-level ray interaction metadata (e.g., reflection counts, diffraction depth, interaction density), the framework enables multi-task learning capabilities, including:

- Propagation regime classification (LOS/NLOS, urban canyon, open field);
- Channel complexity estimation (multipath richness indices);
- Rain attenuation modeling;
- Reliability and coverage probability assessment;
- Directional beam alignment and sector classification.

This elevates the digital twin abstraction from map reconstruction to propagation-aware decision modeling. Such a framework is particularly relevant for next-generation Wi-Fi 7 and emerging 6G environments, where higher frequencies, wider bandwidths, and dense deployments increase sensitivity to environmental detail.

### 7.5. Limitations and Modeling Assumptions

Although the ray-tracing engine provides high-fidelity deterministic modeling, the accuracy of the generated dataset depends on environmental reconstruction quality. Simplified building geometries, homogeneous material assumptions, and the absence of dynamic elements (e.g., vehicles, pedestrians, vegetation movement) may introduce deviations from real-world measurements. Additionally, stochastic fading effects and frequency-selective small-scale variations are not explicitly modeled in the deterministic framework.

Nevertheless, validation studies in the literature indicate that deterministic ray tracing achieves strong spatial correlation with measured data when sufficient geometric detail is incorporated. In this context, the present work adopts ray tracing as a physics-grounded digital twin generator, acknowledging that improvements in 3D environmental modeling and material parameter estimation will further enhance surrogate model realism.

### 7.6. Outlook

The results suggest that structured digital twin datasets combined with advanced machine learning models provide a viable pathway toward scalable, AI-assisted wireless network planning. As simulation engines evolve to incorporate richer environmental detail and hybrid deterministic–stochastic channel models, the surrogate modeling framework proposed in this work can be readily extended to support multi-objective optimization and cross-scenario generalization in future studies.

## 8. Limitations

Although the proposed framework enables scalable and physically grounded wireless propagation dataset generation, several limitations related to environmental modeling fidelity, propagation mechanism representation, and validation scope should be acknowledged.

The accuracy of deterministic ray-tracing simulations is strongly influenced by the level of geometric and electromagnetic detail included in the environmental model. In this study, the simulated urban environment was constructed using MATLAB geographic ray-tracing capabilities combined with OpenStreetMap-derived building data. While this approach enables automated and scalable 3D environment reconstruction across large areas, it represents buildings using simplified geometric structures (rectangles) and uniform material properties (concrete or bricks for buildings). In real environments, building facades consist of heterogeneous materials such as concrete, bricks, glass windows, metallic components, insulation layers, and architectural irregularities, each exhibiting distinct electromagnetic characteristics. The use of homogeneous material assumptions may therefore limit the ability to capture fine-grained signal scattering and penetration effects.

In addition, the employed ray-tracing configuration primarily models dominant deterministic propagation mechanisms, including reflections and diffractions. Other propagation phenomena, such as diffuse scattering caused by rough surfaces and vegetation, as well as detailed transmission and refraction through heterogeneous materials, are not represented as of this time due to a lack of MATLAB implementation for such features. These effects can contribute to small-scale fading and additional multipath variability observed in real-world wireless propagation environments.

Another limitation arises from environmental feature representation. Vegetation, foliage attenuation, and small-scale structural objects are not currently supported within MATLAB geographic ray-tracing modeling. Such environmental elements may influence signal attenuation and scattering behavior, particularly in campus environments that contain green areas and irregular urban structures.

Furthermore, this study focuses on simulation-driven digital twin modeling and Machine Learning surrogate dataset generation. Direct large-scale experimental validation of the generated radio maps using real-world measurement campaigns was outside the scope of the present work. While deterministic ray tracing is widely recognized as one of the most physically grounded propagation modeling approaches available, experimental validation (see [13]) would provide additional quantitative assessment of localized prediction accuracy.

Finally, the trade-off between environmental realism and simulation scalability represents an inherent limitation of large-scale propagation modeling. High-fidelity reconstruction of real-world environments using detailed architectural 3D models can significantly improve simulation accuracy but requires extensive modeling effort, specialized design data, and increased computational complexity (see Section 9).

These limitations reflect practical modeling assumptions required to enable large-scale dataset generation and scalable Machine Learning-based wireless propagation analysis. The results presented in this work should therefore be interpreted within the context of digital twin-based simulation frameworks designed to support network planning, research experimentation, and academic wireless communication analysis.

## 9. Future Work

While the results presented in this work demonstrate the effectiveness of combining deterministic ray tracing with Machine Learning for scalable Wi-Fi 7 network analysis, several important extensions are envisioned to further enhance realism, generality, and applicability.

### 9.1. High-Fidelity 3D Campus Environment Regeneration

**Figure 36:**
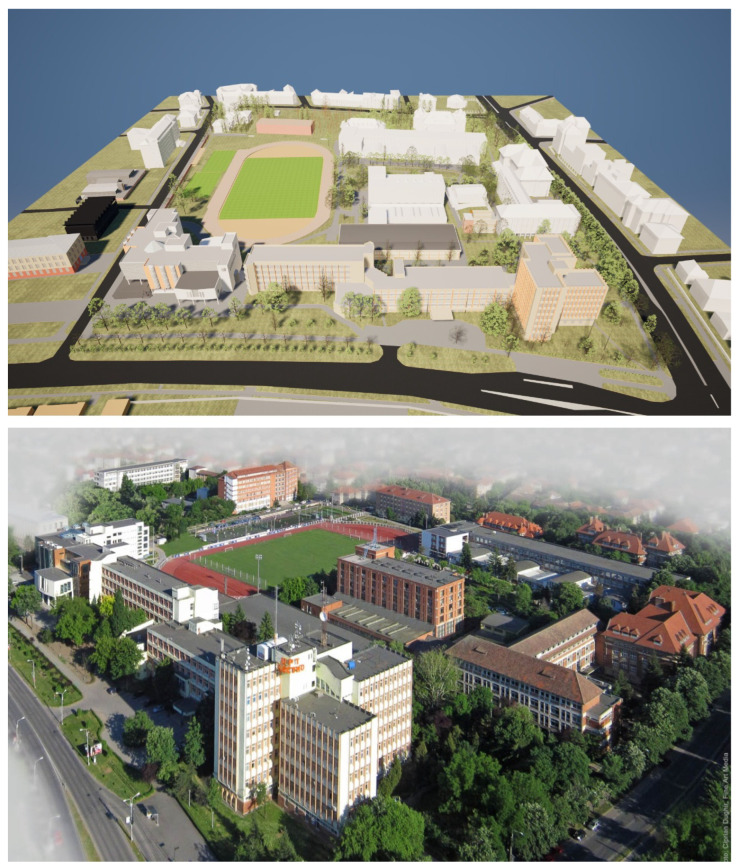
Comparison between the high-resolution 3D digital model (**top**) and the real aerial view (**bottom**) of the Polytechnic University of Timișoara campus. The digital twin, obtained from architectural designs and LiDAR-based reconstructions, closely matches the real-world layout in terms of building geometry, open areas, and vegetation distribution.

A key next step is the regeneration of the entire dataset using a significantly more realistic three-dimensional representation of the Politehnica University of Timișoara campus. Figure 36 illustrates a high-resolution 3D model of the campus, provided in .stl format by the Architecture Faculty of Polytechnic University Timisoara. The model captures fine-grained outdoor building geometry, including detailed façades, rooftops, and surrounding urban structures reconstructed from LiDAR scans. In addition, the environment explicitly includes volumetric representations of vegetation, such as trees in the surrounding park areas, which are absent from the simplified models used in the current study.

Incorporating this realistic geometry is expected to substantially alter the propagation characteristics, particularly with respect to reflection density, diffraction paths, and shadowing effects. Vegetation, in particular, introduces frequency-dependent attenuation and scattering phenomena that are not captured in building-only models. Regenerating the dataset within this environment will therefore provide a more faithful representation of real-world outdoor deployments and will enable the trained surrogate models to generalize better to practical scenarios.

**Figure 37:**
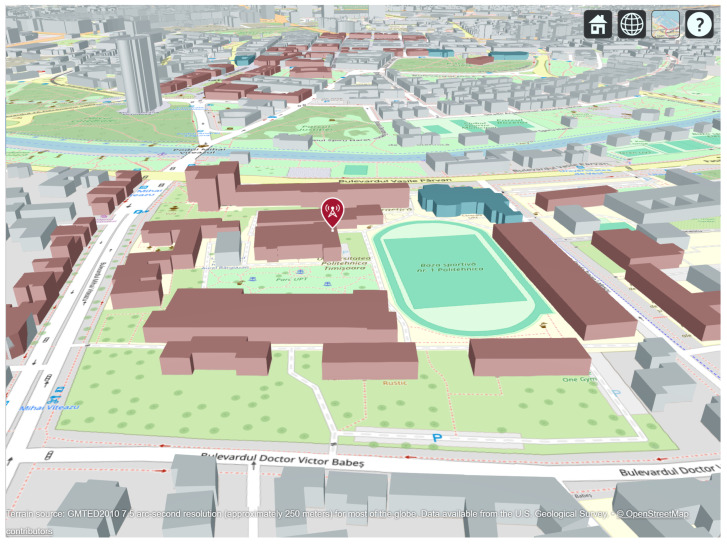
OpenStreetMap (OSM)-derived geographic 3D reconstruction of the Campus used in this study. The OSM model represents buildings as simplified extruded volumes, while the .stl model in Figure 36 incorporates detailed façades, rooftops, and vegetation reconstructed by the Architecture Faculty of Polytechnic University Timisoara. The visual comparison highlights the increased geometric fidelity of the .stl environment.

For comparison purposes, Figure 37 also includes the OpenStreetMap (OSM)-derived geographic 3D reconstruction used in the present study. Unlike the high-fidelity .stl model, the OSM-based environment represents buildings primarily as simplified extruded blocks with limited façade detail and without explicit vegetation modeling. This side-by-side visualization enables qualitative assessment of geometric differences between the simplified geographic representation and the architecturally detailed STL (Cartesian) model. The comparison illustrates the substantially higher structural realism of the STL model, which was developed through architectural design data and LiDAR-supported reconstruction by the Architecture Faculty of Polytechnic University Timisoara.

### 9.2. Toward 6G and mmWave Micro/Pico-Cell Evaluation

The proposed framework opens the door to early-stage exploration of beyond-Wi-Fi 7 and pre-6G scenarios. A particularly relevant direction is the evaluation of Voice over New Radio (VoNR) micro- and pico-cell deployments operating at millimeter-wave (mmWave) frequencies (24–100 GHz). At such frequencies, propagation becomes highly sensitive to blockage, diffraction scarcity, and beam alignment, making exhaustive ray tracing prohibitively expensive at scale. The combination of high-fidelity ray-traced data and Machine Learning-based surrogate modeling offers a promising approach for rapid performance assessment, what-if analysis, and network planning in future ultra-dense 6G environments.

### 9.3. Extension to Beamforming and Intelligent Transmission Strategies

Beyond omnidirectional transmission with fixed dipole antennas, future work will investigate adaptive antenna systems and beamforming strategies. By extending the ray-tracing simulations to directional antennas and electronically steerable beams, the dataset can be enriched with angular power distributions, beam indices, and directional gain patterns. Machine Learning models trained on such data could then be used to predict optimal beam directions, evaluate coverage trade-offs, and support fast beam selection in dense urban environments. This naturally aligns with digital twin-driven network optimization, where learned surrogate models act as real-time engines for coverage estimation and configuration exploration.

## 10. Conclusions

This work presented a comprehensive framework for 3D outdoor Wi-Fi 7 network planning and analysis that integrates high-fidelity MATLAB ray-tracing simulations with data-driven Machine Learning surrogate models. Using a realistic urban campus environment, extensive ray-tracing datasets were generated across multiple frequencies (2.4, 5, and 6 GHz), transmit power levels, and propagation configurations, capturing the complex effects of reflections, diffractions, and spatial geometry on received signal strength.

To address the prohibitive computational cost of exhaustive ray-tracing simulations, Transformer-based regression models were investigated as fast and accurate surrogates. Among the evaluated approaches, the FT-Transformer demonstrated a strong balance between predictive accuracy, robustness, and computational efficiency. It achieved high $R^{2}$ scores on both validation and test sets while maintaining a compact model size and low inference latency, enabling RSSI predictions in seconds compared to months of offline ray-tracing execution on multi-CPU servers.

The results confirm that Machine Learning models can effectively learn the underlying structure of ray-tracing-derived propagation data and generalize across frequencies, power levels, and spatial receiver configurations. This capability enables rapid scenario exploration, high-resolution coverage estimation, and scalable what-if analysis that would be infeasible using physics-based simulations alone. Rather than replacing ray tracing, the proposed approach complements it by transforming computationally intensive offline simulations into efficient, reusable digital surrogates.

Overall, the presented framework establishes a practical foundation for AI-assisted wireless network planning and digital twin applications. By combining physically grounded simulations with modern transformer architectures, it opens the path toward real-time, data-driven optimization of future Wi-Fi and beyond-5G/6G networks in complex urban environments.

## Figures and Tables

**Table 1:** Statistical summary of RSSI distributions (in dBm) for the 2.4 GHz, 5 GHz, and 6 GHz Wi-Fi bands across all ray-tracing configurations.

Frequency [GHz]	Count	Mean	Median	Std	Min	P5	P25	P75	P95	Max	IQR
2.4	1,076,698	−65.05	−62.82	16.06	−150.12	−92.96	−75.96	−52.16	−43.93	−35.06	23.80
5.0	1,081,920	−72.00	−69.47	16.37	−156.58	−100.42	−83.31	−58.79	−50.42	−41.84	24.52
6.0	1,049,220	−73.34	−70.56	16.48	−158.17	−102.25	−84.65	−60.13	−51.88	−42.40	24.52

**Table 2:** FT-Transformer RSSI [dBm] results over five independent runs. Metrics reported per split; bottom rows show mean and standard deviation across runs.

Run	$R_{\mathrm{train}}^{2}$	$R_{\mathrm{val}}^{2}$	$R_{\mathrm{test}}^{2}$	MAE_train_ [dBm]	RMSE_train_ [dBm]	MAE_val_ [dBm]	RMSE_val_ [dBm]	MAE_test_ [dBm]	RMSE_test_ [dBm]
1	0.9971	0.9962	0.9962	0.9536	1.4632	1.0510	1.6675	1.0502	1.6670
2	0.9967	0.9958	0.9958	1.0158	1.5602	1.1067	1.7559	1.1093	1.7494
3	0.9969	0.9960	0.9960	0.9936	1.5124	1.0872	1.7178	1.0858	1.7153
4	0.9976	0.9968	0.9968	0.8735	1.3382	0.9579	1.5333	0.9576	1.5252
5	0.9974	0.9966	0.9967	0.9045	1.3849	0.9908	1.5795	0.9903	1.5654
**Mean**	**0.9971**	**0.9963**	**0.9963**	**0.9482**	**1.4518**	**1.0387**	**1.6508**	**1.0386**	**1.6445**
**Std**	**0.00036**	**0.00041**	**0.00044**	**0.05948**	**0.09074**	**0.06315**	**0.09314**	**0.06375**	**0.09619**

Each run was trained for up to 500 epochs. A new random 70/15/15 data split (train/validation/test) was generated for every run, ensuring statistical independence across experiments. The best model was selected (among the 500 epochs) based on maximum training $R^{2}$.

**Table 3:** RandomForestRegressor RSSI [dBm] results over five independent runs. Metrics reported per split; bottom rows show mean and standard deviation across runs.

Run	$R_{\mathrm{train}}^{2}$	$R_{\mathrm{val}}^{2}$	$R_{\mathrm{test}}^{2}$	MAE_train_ [dBm]	RMSE_train_ [dBm]	MAE_val_ [dBm]	RMSE_val_ [dBm]	MAE_test_ [dBm]	RMSE_test_ [dBm]
1	0.9993	0.9947	0.9947	0.3329	0.7318	0.9017	1.9685	0.8946	1.9625
2	0.9993	0.9947	0.9946	0.3337	0.7332	0.9029	1.9695	0.8975	1.9676
3	0.9993	0.9947	0.9946	0.3334	0.7331	0.9017	1.9669	0.8969	1.9681
4	0.9993	0.9947	0.9947	0.3326	0.7316	0.9011	1.9676	0.8952	1.9624
5	0.9993	0.9947	0.9946	0.3334	0.7335	0.9019	1.9683	0.8956	1.9669
**Mean**	**0.9993**	**0.9947**	**0.9946**	**0.3334**	**0.7331**	**0.9017**	**1.9683**	**0.8956**	**1.9669**
**Std**	**0.00000**	**0.00000**	**0.00005**	**0.00044**	**0.00087**	**0.00065**	**0.00098**	**0.00121**	**0.00282**

A new random 70/15/15 data split (train/validation/test) was generated for every run, ensuring statistical independence across experiments.

**Table 4:** XGBoost RSSI [dBm] results over five independent runs. Metrics reported per split; bottom rows show mean and standard deviation across runs.

Run	$R_{\mathrm{train}}^{2}$	$R_{\mathrm{val}}^{2}$	$R_{\mathrm{test}}^{2}$	MAE_train_ [dBm]	RMSE_train_ [dBm]	MAE_val_ [dBm]	RMSE_val_ [dBm]	MAE_test_ [dBm]	RMSE_test_ [dBm]
1	0.9995	0.9945	0.9945	0.4044	0.5999	1.0024	1.9263	1.0078	1.9307
2	0.9995	0.9944	0.9944	0.4010	0.5957	1.0073	1.9378	1.0149	1.9425
3	0.9995	0.9943	0.9942	0.4070	0.6034	1.0458	1.9573	1.0547	1.9733
4	0.9995	0.9944	0.99444	0.3997	0.5884	1.0236	1.9345	1.0281	1.9362
5	0.9995	0.9944	0.9943	0.4085	0.6041	1.0143	1.9351	1.0208	1.9528
**Mean**	**0.9995**	**0.9944**	**0.9944**	**0.4041**	**0.5983**	**1.0187**	**1.9382**	**1.0253**	**1.9471**
**Std**	**0.00001**	**0.00007**	**0.00010**	**0.0038**	**0.0065**	**0.0171**	**0.0115**	**0.0181**	**0.0168**

A new random 70/15/15 data split (train/validation/test) was generated for every run, ensuring statistical independence across experiments.

**Table 5:** Comparison of FT-Transformer RandomForestRegressor, and XGBoost on RSSI [dBm] prediction. Values shown are mean ± standard deviation over five independent runs.

Model	$R_{\mathrm{train}}^{2}$	$R_{\mathrm{val}}^{2}$	$R_{\mathrm{test}}^{2}$	MAE_train_	RMSE_train_	MAE_val_	RMSE_val_	MAE_test_	RMSE_test_
**FT-Transformer**	0.9971±0.00036	0.9963±0.00041	0.9963±0.00044	0.9482±0.05948	1.4518±0.09074	1.0387±0.06315	1.6508±0.09314	1.0386±0.06375	1.6445±0.09619
**RF-Regressor**	0.9993±0.00000	0.9947±0.00000	0.9946±0.00005	0.3334±0.00044	0.7331±0.00087	0.9017±0.00065	1.9683±0.00098	0.8956±0.00121	1.9669±0.00282
**XGBoost**	0.9995±0.00001	0.9944±0.00007	0.9944±0.00010	0.4041±0.0038	0.5983±0.0065	1.0187±0.0171	1.9382±0.0115	1.0253±0.0181	1.9471±0.0168

**Table 6:** FT-Transformer fine-tuning results for RSSI prediction [dBm] over five independent runs using a fixed file-based split.

Run	$R_{\mathrm{train}}^{2}$	$R_{\mathrm{val}}^{2}$	$R_{\mathrm{test}}^{2}$	MAE_train_	RMSE_train_	MAE_val_	RMSE_val_	MAE_test_	RMSE_test_
Run #1	0.9955	0.9532	0.8003	1.1605	1.7763	3.5178	5.3811	4.7906	6.5370
Run #2	0.9953	0.9536	0.8041	1.1860	1.8130	3.4920	5.3570	4.7410	6.4750
Run #3	0.9968	0.9559	0.8103	0.9734	1.5080	3.3892	5.2230	4.6377	6.3727
Run #4	0.9955	0.9532	0.8003	1.6052	1.7763	3.5178	5.3811	4.7906	6.5371
Run #5	0.9954	0.9564	0.8074	1.1646	1.7955	3.3962	5.1931	4.7101	6.4201
**Avg**	**0.9955**	**0.9536**	**0.8041**	**1.1646**	**1.7763**	**3.4920**	**5.3570**	**4.7410**	**6.4750**
**Std**	0.00062	0.00156	0.00440	0.23288	0.12716	0.06472	0.09153	0.06384	0.07239

**Figure A1:**
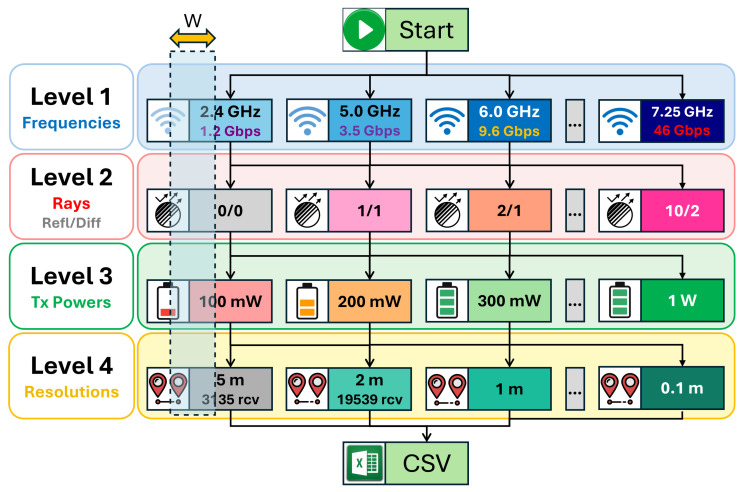
Hierarchical configuration flowchart used for generating and managing ray-tracing simulation scenarios. Each path from top to bottom defines a unique simulation configuration exported as a CSV file.

**Figure A2:**
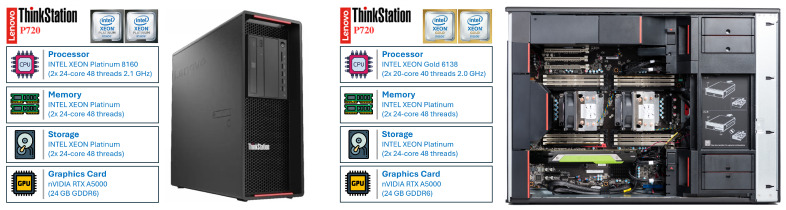
Hardware configuration of the primary ray-tracing and machine learning simulation servers.

**Figure A3:**
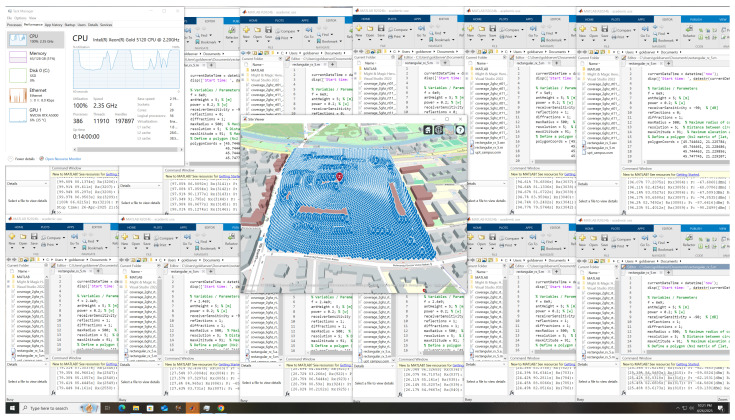
Screenshot of 12 parallel MATLAB R2024b instances running on the Lenovo ThinkStation P720, powered by 2 x Intel Xeon Gold 6138 CPUs. Each instance executes a distinct ray-tracing configuration, achieving full CPU utilization across all logical cores.

**Figure A4:**
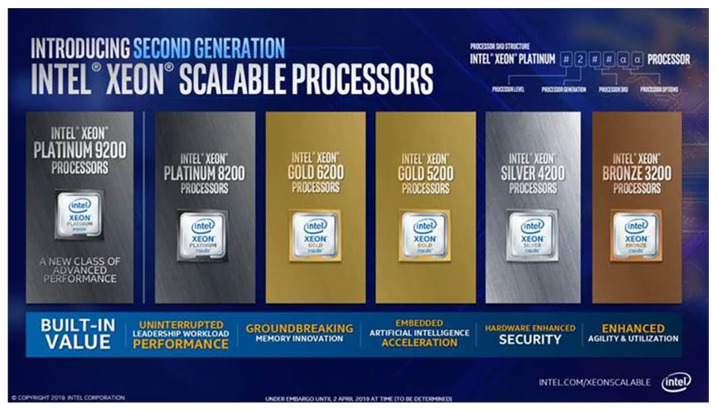
Reference diagram illustrating Intel Xeon Scalable processor tiers. This architecture overview highlights the positioning of Platinum- and Gold-series CPUs used in this study. One of our primary simulation servers (Platinum Server) was equipped with two Intel Xeon Platinum CPUs (8160, 24-core, 2.1 GHz, 33 MB cache), while the secondary server (Gold Server) used an Intel Xeon Gold-class CPU (6138, 20-core 2.00 GHz, 27.5 MB cache). This distinction reflects a performance hierarchy in compute capacity and parallel instance throughput.

## Data Availability

The data presented in this study are available from the corresponding author upon reasonable request.

## Abbreviations

The following abbreviations are used in this manuscript:

DTC	Digital Twin Channel
DTWC	Digital Twin Wireless Channel
AP	Access Point
RT	Ray-tracing
R/D	Reflections/Diffractions
REM	Radio Environment Map
RSS	Received Signal Strength
RSSI	Received Signal Strength Indicator
ML	Machine Learning
AI	Artificial Intelligence
IoT	Internet of Things
OSM	OpenStreetMap
Tx	Transmitter
Rx	Receiver
CPU	Central Processing Unit
CSV	Comma-Separated Values
MLO	Multi-Link Operation
QAM	Quadrature Amplitude Modulation

## Appendix A. Ray-Tracing Execution Setup

To efficiently explore the parameter space of our ray-tracing simulations, we implemented a hierarchical configuration strategy and executed it in parallel across multiple servers and MATLAB instances. The structure of this process is illustrated in Figure A1.

The configuration space is organized into four levels:

- Level 1—Frequencies: We simulate signal propagation for wireless frequencies including 2.4 GHz, 5.0 GHz, and 6.0 GHz. Each frequency corresponds to a nominal modulation capacity (e.g., 1.2 Gbps for 2.4 GHz, 9.6 Gbps for 6.0 GHz). Additional higher-frequency bands such as 7.25 GHz may also be considered in extended studies.
- Level 2—Ray Configurations: MATLAB’s ray-tracing engine supports a maximum of 10 reflections and 2 diffractions. However, due to the significant increase in computational time when simulating two diffractions, we limited this study to a maximum of 10 reflections and 1 diffraction. Each configuration is represented as R/D, denoting the number of allowed reflections (R) and diffractions (D), e.g., 0/0, 1/0, *…*, 10/0, and 0/1, 1/1, *…*, 10/1.
- Level 3—Transmit Power Levels: For each ray configuration, we simulate four transmit power levels: 0.1 W, 0.2 W, 0.3 W, and 1.0 W. These settings allow us to investigate signal propagation under various transmission conditions.
- Level 4—Spatial Resolutions: Receiver positions are sampled with spatial resolutions of 5 m, 2 m, 1 m, and 0.1 m. The number of receivers increases accordingly, from 135 receivers at 5 m spacing to over 19,000 at 2 m resolution. The 0.1 m setting was primarily used in isolated test cases due to its high computational cost.

To manage the large number of simulations, we distributed the workload across four parallel servers. Each server executed 3, 6, 9, or 12 MATLAB instances concurrently, depending on resource availability. Each instance processed up to 3210 receiver positions per configuration, iterating through all selected combinations of frequency, propagation complexity, transmit power, and spatial resolution.

Simulation outputs were saved as structured CSV files, one per configuration, for reproducibility and downstream analysis. This hierarchical, parallelized setup enabled wide coverage of the parameter space within practical hardware and software limits.

## Appendix B. Dataset Description and Feature Definitions

This appendix provides a detailed description of the dataset generated and used in this study. The dataset was obtained through extensive MATLAB-based ray-tracing simulations conducted over a large-scale urban campus environment and serves as the foundation for all machine learning experiments presented in the paper.

Each row in the dataset corresponds to a single transmitter–receiver (Tx–Rx) link evaluated under a specific combination of frequency band, transmit power, ray-tracing configuration, and receiver location. The dataset is designed to preserve both the physical interpretability of wireless propagation and the statistical richness required for data-driven modeling, neural architecture search, and digital twin integration.

### Appendix B.1. Dataset Design Philosophy

The dataset follows three core design principles:

- Physics-preserving: All features originate directly from deterministic ray-tracing outputs or geometric calculations, ensuring alignment with electromagnetic propagation theory.
- Model-agnostic: No handcrafted feature engineering or dimensionality reduction was applied prior to learning, allowing different model families (tree-based, neural, transformer-based) to exploit the data according to their inductive biases.
- Scalable and extensible: The feature schema supports future extensions such as adaptive antennas, beamforming, mobility, and dynamic environmental changes.

This design enables the dataset to function not only as a training corpus, but also as a reusable asset for future studies on network optimization and AI-driven radio planning.

### Appendix B.2. Feature Categories

For interpretability and clarity, the dataset columns can be grouped into the following categories:

- Identifiers and metadata, used for indexing, bookkeeping, and traceability.
- Transmitter and receiver geometry, describing spatial placement, distance, and relative orientation.
- Radio configuration parameters, defining frequency, transmit power, and antenna height.
- Ray-tracing interaction statistics, capturing multipath complexity through reflections and diffractions.
- Target variables, representing received signal strength.
- Auxiliary performance metrics, such as simulation runtime.

This categorization mirrors the structure of the MATLAB simulation pipeline and facilitates direct interpretation of learned feature importance and correlations.

### Appendix B.3. Complete Feature List

Table A1 provides the complete list of dataset columns, along with concise descriptions and measurement units.

### Appendix B.4. Target Variables

The supervised learning targets in this study are rssi1 and rssi2, which represent two independently computed received power estimates provided by MATLAB’s propagation framework. Unless explicitly stated otherwise, rssi1 is used as the primary regression target due to its numerical stability and consistent behavior across simulation configurations. Both targets are retained to support future multi-output learning scenarios and uncertainty analysis.

**Table A1:** Complete list of dataset columns and descriptions used in this study.

#	Column Name	Description
1	receiver_index	Unique identifier for each receiver position within the circular grid layout
2	tx_latitude	Latitude of the transmitter in decimal degrees [°]
3	tx_longitude	Longitude of the transmitter in decimal degrees [°]
4	tx_frequency	Transmission frequency [Hz], spanning 2.4, 5, and 6 GHz bands
5	tx_power	Transmit power [W] (100–1000 mW)
6	tx_antenna_height	Transmitter antenna height above ground [m]
7	tx_elevation	Terrain elevation at transmitter site [m]
8	rx_latitude	Receiver latitude in decimal degrees [°]
9	rx_longitude	Receiver longitude in decimal degrees [°]
10	rx_antenna_height	Receiver antenna height above ground [m]
11	rx_elevation	Terrain elevation at receiver location [m]
12	rx_sensitivity	Receiver sensitivity threshold [dBm]
13	distance_tx_rx	Tx–Rx separation distance [m]
14	azimuth_angle	Horizontal bearing from transmitter to receiver [°]
15	elevation_angle	Vertical angle between transmitter and receiver [°]
16	rt_reflections	Maximum number of reflections allowed by the simulation configuration
17	rt_diffractions	Maximum number of diffractions allowed by the simulation configuration
18	nb_rays	Total number of rays launched by the SBR engine
19	nb_interactions	Total number of ray interactions (reflections + diffractions)
20	len_interactions	Number of interaction hops along the dominant ray path
21	ray_max_nb_reflections	Maximum reflections encountered by any single ray
22	ray_max_nb_diffractions	Maximum diffractions encountered by any single ray
23	total_nb_reflections	Total reflection events aggregated across all rays
24	total_nb_diffractions	Total diffraction events aggregated across all rays
25	rssi1	Received Signal Strength Indicator (RSSI) [dBm]
26	rssi2	Received Signal Strength Indicator (RSSI), heavy rain [dBm]
27	elapsed_time	Simulation runtime per receiver position [ms]

### Appendix B.5. Feature Usage in Machine Learning

All numerical features, excluding identifiers and runtime metadata, are retained during training. Columns such as receiver_index and elapsed_time are excluded from the learning process but preserved for traceability, debugging, and computational analysis.

No features were removed solely based on correlation strength. This decision reflects the adoption of transformer-based architectures, which are capable of learning complex, nonlinear interactions between correlated inputs without manual feature pruning. As a result, the dataset maintains its full descriptive richness while enabling the model to learn both dominant and subtle propagation effects.

### Appendix B.6. Forward Compatibility

Although the current dataset uses a fixed, center-fed dipole antenna at a static transmitter location, the feature schema is intentionally forward-compatible. Angular features, interaction counts, and geometric descriptors are expected to play a significantly larger role in future extensions involving directional antennas, beamforming, mobility, and adaptive transmission strategies within a digital twin framework.

This makes the dataset suitable not only for the experiments presented in this paper, but also for continued research on AI-native wireless network planning and optimization.

## Appendix C. Server Infrastructure and Parallel Execution Strategy

To manage the large-scale parameter space of the ray-tracing simulations, we deployed a parallel execution framework across four dedicated simulation servers. Each server was equipped with multi-core CPUs capable of running several independent MATLAB R2024b instances simultaneously. Every instance executed a distinct configuration from the hierarchical space detailed in Appendix A.

The configuration distribution was performed manually by partitioning sets of receiver positions and ray-tracing parameters, including combinations of frequency (2.4 GHz, 5.0 GHz, 6.0 GHz), transmit power levels, and propagation complexity (reflections/diffractions). Each MATLAB process independently loaded a receiver grid and completed a batch of simulations, writing outputs to structured CSV files.

- Platinum Server: Lenovo ThinkStation P720, powered by an Intel Xeon Platinum 8160 CPU with 24 cores. It was configured to run 12 parallel MATLAB instances at 100% CPU utilization. This high-end workstation was the most productive node, suitable for long-duration, high-resolution runs.
- Gold Server: Lenovo ThinkStation P720 with Intel Xeon Gold 6138 (20 cores). Typically ran 3–12 MATLAB instances concurrently.
- Other Nodes: Two additional servers with similar Intel Xeon CPUs (Silver-series), configured for 3–6 MATLAB windows depending on core count and thermal limits.

Each MATLAB window acted independently, ensuring robustness and parallelism without shared memory dependencies. Simulation logs were saved per instance, and failures could be recovered by restarting individual windows. This parallel strategy enabled the evaluation of thousands of configurations in a fraction of the time required for sequential execution.

Figure A3 shows the Gold server running 12 MATLAB R2024b instances in parallel, actively processing spatially distributed receiver points across the defined simulation region.

### CPU Classes and Server Roles

The simulation workloads in this study were executed across two primary server types, each equipped with different classes of Intel Xeon CPUs. The primary node, referred to as the Platinum Server, is a Lenovo ThinkStation P720 configured with two Intel Xeon Platinum 8160 processors (24 cores, 48 threads each). This dual-socket configuration offered 96 logical threads, allowing for highly parallelized MATLAB execution across up to 12 simultaneous instances with sustained performance.

In contrast, the secondary node, referred to as the Gold Server, is a Lenovo ThinkStation P720 equipped with a single Intel Xeon Gold 6138 CPU (20 cores, 40 threads). Although still capable of multi-instance execution, its total compute capacity was comparable to Platinum, supporting between 3 and 12 parallel MATLAB instances depending on ray-tracing configuration (the higher the number of reflections and diffractions used, the lower the number of parallel MATLAB instances used).

Figure A4 provides an architectural overview of Intel Xeon Scalable processor tiers. It highlights the relative positioning of the Gold and Silver series CPUs used in this study, emphasizing the intended segmentation of compute performance and scalability in server-grade hardware.

Intel’s second-generation Xeon Scalable processors are organized into performance tiers designed for various levels of compute intensity and scalability, as illustrated in Figure A4. The processors range from Bronze for entry-level workloads to Platinum for mission-critical multi-socket applications.

The simulations in this study were executed on 2 servers, one equipped with 2 Intel Xeon Platinum 8160 CPUs which belong to the Platinum 8200 series, another one equipped with 2 Intel Xeon Gold 6138 CPUs which belong to the Platinum 6200 series. These processors support Intel^®^ Deep Learning Boost (DL Boost) via Vector Neural Network Instructions (VNNI), providing native acceleration for deep learning inference tasks. This enables moderate AI workloads, such as signal classification or anomaly detection, to be executed on the CPU without requiring a discrete GPU, reducing latency and power consumption.

While the focus of this study was ray-tracing simulation, the choice of hardware positions the system for future integration of AI-assisted modeling, hybrid simulation-learning workflows, or adaptive signal processing pipelines that benefit from on-chip AI capabilities.

## Appendix D. Electromagnetic Material Parameters Used in Ray-Tracing Simulations

### Appendix D.1. Material Assignment from OpenStreetMap Data

The three-dimensional urban environment used in this study was reconstructed from OpenStreetMap (OSM) building layers and imported into MATLAB’s siteviewer environment. During the import process, building objects were automatically classified according to available OSM material tags. Electromagnetic parameters were then assigned to each material category.

The material categories were detected and used in the simulations are presented in Table A2.

**Table A2:** Electromagnetic properties assigned to the material labels used in the simulations.

Material Label	$\varepsilon_{r}^{\prime }$	σ [S/m]	$\varepsilon_{r}^{*}$
"" (unspecified)	1.0	0	1+0i
brick	3.91	0.027379	3.91−0.20506i
concrete	5.31	0.0326	5.31−0.244i
glass	6.31	0.011629	6.31−0.0871i
concrete/glass	1.0	0	1+0i

When material information was missing or ambiguous (e.g., empty string or combined tags such as “concrete/glass”), MATLAB assigned default free-space parameters ($\varepsilon_{r}=1$, σ=0). This approach ensures numerical stability of the ray-tracing engine while preserving deterministic propagation behavior.

### Appendix D.2. Complex Permittivity Formulation and Electromagnetic Interpretation

In frequency-domain electromagnetic modeling, material response is characterized using the complex relative permittivity:

(A1) $$\varepsilon_{r}^{*}=\varepsilon_{r}^{\prime }-j\frac{\sigma }{\omega \varepsilon_{0}}$$

where:

- $\varepsilon_{r}^{\prime }$ is the real relative permittivity;
- σ is the electrical conductivity (S/m);
- ω=2πf is the angular frequency;
- $\varepsilon_{0}$ is the vacuum permittivity;
- *j* is the imaginary unit.

The imaginary term represents dielectric losses associated with finite conductivity. The magnitude of this loss component increases with conductivity and frequency.

For the materials used in this study, the complex permittivity values at WiFi frequencies (2.4–6 GHz) are:

(A2) $$\;\;\;\;\;\;\;\;\;\;\;\mathrm{Brick}:\;\varepsilon_{r}^{*}=3.91-j\;0.20506$$

(A3) $$\mathrm{Concrete}:\;\varepsilon_{r}^{*}=5.31-j\;0.244$$

(A4) $$\;\;\;\;\;\;\;\;\;\;\;\mathrm{Glass}:\;\varepsilon_{r}^{*}=6.31-j\;0.0871$$

These complex permittivity values are internally used by MATLAB’s ray-tracing engine to compute Fresnel reflection coefficients for each specular interaction. Both the magnitude and phase of the reflection coefficient depend directly on $\varepsilon_{r}^{*}$, thereby influencing:

- Reflection loss;
- Phase shift per interaction;
- Multipath interference behavior.

Consequently, material-dependent electromagnetic attenuation is inherently incorporated into each reflected propagation path within the deterministic ray-tracing framework.

## Appendix E. Dataset Multiple Learning Paradigms

### Appendix E.1. Regression Tasks

#### Appendix E.1.1. Signal-Level Predictions

Path loss quantifies the attenuation experienced by a radio signal as it propagates from the transmitter to the receiver. It is commonly expressed in decibels (dB) as the difference between the transmitted power and the received power:

(A5) $$PL\;\left[\mathrm{dB}\right]=P_{tx}\;\left[\mathrm{dBm}\right]-P_{rx}\;\left[\mathrm{dBm}\right],$$

where $P_{tx}$ denotes the transmit power in dBm and $P_{rx}$ corresponds to the received signal strength indicator (RSSI).

In the context of the present dataset, path loss can be directly computed from the available transmit power and RSSI values without additional simulations. Unlike RSSI, which depends on the selected transmit power configuration, path loss characterizes the intrinsic propagation channel and is therefore a power-independent descriptor of environmental attenuation effects. Predicting path loss enables standardized channel modeling, multi-frequency comparison, and improved generalization across different transmission configurations.

Link margin represents the reliability buffer of a wireless communication link and is defined as the difference between the received power and the receiver sensitivity threshold (−90 in our case):

(A6) $$LM\;\left[\mathrm{dB}\right]=P_{rx}\;\left[\mathrm{dBm}\right]-P_{sens}\;\left[\mathrm{dBm}\right],$$

where $P_{rx}$ is the received signal strength (RSSI) and $P_{sens}$ denotes the minimum receiver sensitivity required for successful signal demodulation and decoding.

A positive link margin (LM>0) indicates that the received signal exceeds the minimum required threshold and the link is expected to be operational, while a negative link margin (LM<0) implies insufficient signal strength and potential communication failure.

Given that both RSSI and receiver sensitivity are available in the dataset, link margin can be directly computed and predicted as a regression or classification target. Modeling link margin is particularly valuable for wireless network planning, as it enables coverage reliability assessment, robustness analysis under varying environmental conditions (e.g., rain attenuation), and optimization of transmit power and antenna configurations to ensure adequate communication safety margins.

The signal-to-noise ratio (SNR) quantifies the quality of a wireless communication link by measuring the difference between the received signal power and the total noise power at the receiver input. It is expressed in decibels (dB) as:

(A7) $$\mathrm{SNR}\;\left[\mathrm{dB}\right]=P_{rx}\;\left[\mathrm{dBm}\right]-P_{noise}\;\left[\mathrm{dBm}\right],$$

where $P_{rx}$ denotes the received signal strength (RSSI) and $P_{noise}$ represents the effective noise power within the receiver bandwidth.

The noise power can be approximated using the thermal noise density integrated over the channel bandwidth *B* and the receiver noise figure NF:

(A8) $$P_{noise}\;\left[\mathrm{dBm}\right]=-174+10\log_{10}\left(B\;\left[\mathrm{Hz}\right]\right)+NF\;\left[\mathrm{dB}\right],$$

where −174 dBm/Hz corresponds to the thermal noise floor at room temperature (approximately 290 K).

Although the present dataset does not explicitly include measured noise or interference power, SNR can be derived from the available RSSI values under assumed receiver bandwidth and noise figure parameters consistent with the target Wi-Fi configuration. This enables SNR to be modeled as a regression target using the existing feature set. Predicting SNR provides additional practical value beyond RSSI alone, as it directly relates to modulation and coding scheme (MCS) selection, achievable throughput, link reliability assessment, and coverage quality classification in wireless network planning applications.

Furthermore, by using the rain-affected RSSI values available in the dataset, a weather-dependent SNR estimate can be obtained as:

(A9) $${\mathrm{SNR}}_{rain}=P_{rx,rain}-P_{noise},$$

enabling analysis of environmental robustness and climate-induced degradation effects on link performance.

Coverage probability represents the likelihood that a wireless link satisfies a predefined reliability threshold and is widely used as a network-level performance metric. For a received signal power threshold γ, coverage probability is defined as:

(A10) $$P_{cov}=P\left(P_{rx}>\gamma \right),$$

where $P_{rx}$ denotes the received power (RSSI). When using receiver sensitivity as the reliability criterion, coverage probability can be equivalently expressed in terms of link margin as:

(A11) $$P_{cov}=P\left(LM>0\right),$$

where $LM=P_{rx}-P_{sens}$.

Given that both RSSI and receiver sensitivity are available in the dataset, coverage probability can be directly computed under different transmission configurations, frequency bands, and environmental conditions (e.g., heavy rain). This enables statistical reliability analysis beyond pointwise signal prediction and supports comparative evaluation of multi-band deployment strategies, transmit power optimization, and environmental robustness assessment within the digital twin framework.

Rain attenuation difference quantifies the additional signal degradation introduced by adverse weather conditions. Let $P_{rx}^{normal}$ denote the received power (RSSI) under normal atmospheric conditions and $P_{rx}^{rain}$ represent the received power under heavy rain. The rain-induced attenuation difference is defined as:

(A12) $$\Delta_{rain}\;\left[\mathrm{dB}\right]=P_{rx}^{normal}-P_{rx}^{rain}.$$

A positive value of $\Delta_{rain}$ indicates additional attenuation due to rainfall. Because both normal and rain-affected RSSI values are available in the dataset, $\Delta_{rain}$ can be directly computed without additional simulation effort.

Modeling rain attenuation difference provides important practical insights beyond signal strength prediction alone. It enables quantitative assessment of weather robustness, frequency-dependent atmospheric sensitivity analysis, and reliability degradation estimation under adverse environmental conditions. Furthermore, $\Delta_{rain}$ supports rain-aware coverage probability analysis and informed network planning decisions, particularly for multi-band deployments where higher frequencies exhibit increased susceptibility to precipitation-induced attenuation.

#### Appendix E.1.2. Channel Complexity Predictions

Multipath richness characterizes the structural complexity of the propagation channel by quantifying the density and depth of ray interactions between transmitter and receiver. Using the ray-tracing metadata available in the dataset, a multipath Richness Index (MRI) can be defined to capture interaction intensity. A basic formulation is:

(A13) $$MRI=\frac{N_{int}}{N_{rays}},$$

where $N_{int}$ denotes the total number of ray interactions (reflections and diffractions) and $N_{rays}$ represents the total number of launched rays.

To account for diffraction-dominated propagation scenarios, a weighted formulation may also be used:

(A14) $$MRI=\alpha \frac{N_{ref}}{N_{rays}}+\beta \frac{N_{diff}}{N_{rays}},$$

where $N_{ref}$ and $N_{diff}$ denote total reflections and diffractions, respectively, and α, β are weighting coefficients reflecting their relative impact on channel dispersion.

Higher MRI values indicate increased multipath complexity, typically associated with non-line-of-sight (NLOS) conditions, enhanced scattering, and potential fading susceptibility. Predicting MRI enables channel complexity prediction, propagation regime classification, and reliability assessment within the digital twin framework.

Effective Interaction Depth (EID) characterizes propagation complexity by quantifying the number of interaction hops required for the dominant propagation path between transmitter and receiver. Using the ray-tracing metadata available in the dataset, a dominant-path effective interaction depth can be defined as:

(A15) $$EID_{dom}=L_{dom},$$

where $L_{dom}$ corresponds to the interaction depth along the dominant ray path (stored as len_interactions). To enable comparison across different ray-tracing configurations, a normalized depth metric can be defined as:

(A16) $$EID_{norm}=\frac{L_{dom}}{R_{max}+D_{max}},$$

where $R_{max}$ and $D_{max}$ denote the maximum numbers of reflections and diffractions allowed by the simulation setup (stored as rt_reflections and rt_diffractions). Higher effective interaction depth values indicate increased propagation complexity and are typically associated with non-line-of-sight (NLOS) scenarios, larger attenuation, and increased multipath dispersion. Predicting effective interaction depth therefore supports propagation regime characterization, channel complexity prediction, and reliability-oriented network planning within the proposed digital twin framework.

Dominant Path Length (DPL) characterizes the geometric extent of the most influential propagation path between transmitter and receiver. In non-line-of-sight (NLOS) conditions, the dominant ray often follows a detoured trajectory due to reflections and diffractions, resulting in an effective propagation distance larger than the Euclidean transmitter–receiver separation. Although the present dataset does not explicitly store per-ray geometric path length, a dominant-path length proxy can be defined using the available transmitter–receiver distance $d_{tx,rx}$ and the dominant interaction depth $L_{dom}$:

(A17) $$DPL=d_{tx,rx}\left(1+\kappa \;L_{dom}\right),$$

where $d_{tx,rx}$ corresponds to distance_tx_rx dataset column, $L_{dom}$ corresponds to len_interactions column in the dataset, and κ is a scaling factor reflecting the average detour contribution per interaction. Alternatively, a dimensionless detour factor can be defined as:

(A18) $$\eta_{d}=\frac{DPL}{d_{tx,rx}},$$

which enables comparison across links of different spatial separations. Higher dominant path length (or detour factor) values indicate increased propagation complexity and are typically associated with additional attenuation and increased channel dispersion. Predicting dominant path length therefore supports propagation regime characterization and reliability-oriented network planning within the proposed structured digital twin framework.

Ray Interaction Density (RID) quantifies the concentration of propagation interactions relative to the transmitter–receiver separation distance and provides a physically interpretable measure of environmental scattering intensity. Using the total number of interactions recorded in the ray-tracing simulation, RID can be defined as:

(A19) $$RID=\frac{N_{int}}{d_{tx,rx}},$$

where $N_{int}$ denotes the total number of ray interactions (reflections and diffractions) and $d_{tx,rx}$ corresponds to the Euclidean transmitter–receiver separation distance (stored as distance_tx_rx column in the dataset).

To account for variations in the number of launched rays, a normalized formulation may also be defined as:

(A20) $$RID_{norm}=\frac{N_{int}}{N_{rays}\cdot d_{tx,rx}},$$

where $N_{rays}$ represents the total number of rays launched by the SBR engine.

Unlike absolute reflection or diffraction counts, RID normalizes interaction intensity by spatial distance, enabling fair comparison across links of different lengths and propagation geometries. Higher RID values indicate denser interaction environments, typically associated with urban canyon scenarios, increased scattering, and elevated multipath-induced fading susceptibility. Consequently, modeling ray interaction density supports propagation regime classification, complexity-aware reliability assessment, and data-driven estimation of fading risk within the proposed structured digital twin framework.

The NLOS Severity Score (NSS) quantifies the degree to which a propagation link is dominated by non-line-of-sight (NLOS) mechanisms. Since diffraction events are typically associated with signal obstruction and shadowing, a diffraction-based severity metric can be defined as:

(A21) $$NSS=\frac{N_{diff}}{N_{int}},$$

where $N_{diff}$ denotes the total number of diffraction events and $N_{int}$ represents the total number of interactions (reflections and diffractions).

To incorporate dominant path depth, a depth-weighted formulation may be defined as:

(A22) $$NSS=\frac{N_{diff}}{N_{int}}\cdot \frac{L_{dom}}{L_{max}},$$

where $L_{dom}$ corresponds to the dominant interaction depth (stored as len_interactions column in the dataset) and $L_{max}$ denotes the maximum interaction depth permitted by the simulation configuration.

Higher NSS values indicate stronger diffraction dominance and deeper interaction chains, typically associated with severe obstruction, increased attenuation, and elevated fading susceptibility. Unlike binary LOS/NLOS classification, NSS provides a continuous measure of obstruction intensity, enabling propagation regime characterization, reliability degradation analysis, and complexity-aware network planning within the structured digital twin framework.

Channel dispersion characterizes the temporal spreading of multipath components and is commonly associated with delay spread and coherence bandwidth in wireless channels. Although the present dataset does not explicitly provide per-ray delay information, a physically motivated dispersion proxy can be derived from the available interaction metadata. A composite Channel Dispersion Proxy (CDP) may be defined as:

(A23) $$CDP=\frac{N_{int}}{N_{rays}}\cdot \frac{L_{dom}}{d_{tx,rx}},$$

where $N_{int}$ denotes the total number of interactions, $N_{rays}$ represents the number of launched rays, $L_{dom}$ corresponds to the dominant interaction depth (stored as len_interactions), and $d_{tx,rx}$ is the transmitter–receiver separation distance.

Higher CDP values indicate increased multipath interaction density and deeper propagation chains relative to spatial separation, typically associated with broader delay spread, reduced coherence bandwidth, and elevated inter-symbol interference risk. Modeling this proxy enables dispersion-aware channel characterization and supports reliability and throughput-oriented network planning within the proposed structured digital twin framework.

### Appendix E.2. Classification Tasks

#### Appendix E.2.1. Line-of-Sight vs. Non-Line-of-Sight Classification

Line-of-sight (LOS) versus non-line-of-sight (NLOS) classification is a fundamental task in wireless propagation modeling, as NLOS conditions typically introduce additional attenuation, stronger fading, and increased channel dispersion. When an explicit LOS indicator is not directly exported by the ray-tracing engine, LOS/NLOS labels can be derived deterministically from ray interaction metadata. In this study, a binary LOS/NLOS label can be defined based on the presence of diffraction events and the dominant interaction depth:

(A24) $$\mathrm{LOS}\Leftrightarrow \left(N_{diff}=0\right)\wedge \left(L_{dom}=0\right),$$

(A25) $$\mathrm{NLOS}\Leftrightarrow \left(N_{diff}>0\right)\vee \left(L_{dom}>0\right),$$

where $N_{diff}$ denotes the number of diffraction events (e.g., total_nb_diffractions) and $L_{dom}$ corresponds to the dominant interaction depth (e.g., len_interactions). These labels enable supervised classification using the structured feature set and support fast propagation regime identification for network planning within the digital twin framework.

Binary LOS/NLOS classification does not fully capture the diversity of non-line-of-sight propagation conditions. In practice, NLOS links may exhibit varying degrees of obstruction severity depending on diffraction dominance and interaction depth. To enable multi-class propagation regime identification, a severity-based classification can be derived from the NLOS Severity Score (NSS).

Using the previously defined NSS metric, links may be categorized as:

- LOS: NSS=0;
- Mild NLOS: $0<NSS\leq \tau_{1}$;
- Moderate NLOS: $\tau_{1}<NSS\leq \tau_{2}$;
- Severe NLOS: $NSS>\tau_{2}$.

Here, $\tau_{1}$ and $\tau_{2}$ are empirically selected thresholds or data-driven percentile values. Severe NLOS conditions typically correspond to diffraction-dominated propagation with deeper interaction chains, resulting in increased attenuation, reduced link margin, and elevated dispersion risk. This multi-level classification provides finer-grained propagation regime characterization compared to binary LOS/NLOS labeling and supports reliability-aware network planning within the digital twin framework.

#### Appendix E.2.2. Propagation Regime Classification

Urban canyon environments are characterized by street-level waveguiding effects induced by tall buildings aligned along narrow corridors. From a propagation perspective, such scenarios typically exhibit reflection-dominated multipath behavior, elevated interaction density, and moderate dominant path depth. Although explicit building morphology parameters are not directly stored in the dataset, urban canyon conditions can be approximated using ray interaction metadata.

A composite Urban Canyon Index (UCI) may be defined as:

(A26) $$UCI=\alpha \cdot \frac{N_{refl}}{N_{int}}+\beta \cdot \frac{N_{int}}{d_{tx,rx}}+\gamma \cdot \frac{L_{dom}}{L_{max}},$$

where $N_{refl}$ denotes the total number of reflections, $N_{int}$ represents total interactions, $d_{tx,rx}$ is the transmitter–receiver separation distance, and $L_{dom}$ is the dominant interaction depth. The weighting coefficients α, β, and γ may be selected based on normalization or empirical calibration.

Higher UCI values indicate reflection-dominated, spatially confined propagation conditions consistent with urban canyon morphology. This classification extends beyond simple LOS/NLOS labeling and enables environment-aware propagation regime identification within the structured digital twin framework.

Open field environments correspond to propagation scenarios with minimal obstruction and weak multipath interaction, where attenuation is primarily distance- and frequency-driven. From a ray-tracing perspective, such links are typically characterized by low interaction counts, negligible diffraction events, low dominant interaction depth, and low interaction density. Using the interaction metadata available in the dataset, open field conditions can be approximated through a composite Open Field Index (OFI) defined as:

(A27) $$OFI=1-\left(\alpha \cdot \frac{N_{int}}{N_{rays}}+\beta \cdot \frac{L_{dom}}{L_{max}}+\gamma \cdot \frac{N_{diff}}{N_{int}+\epsilon }\right),$$

where $N_{int}$ denotes the total number of interactions, $N_{rays}$ is the number of launched rays, $L_{dom}$ is the dominant interaction depth, $N_{diff}$ is the total number of diffraction events, and ϵ is a small constant to avoid division by zero. Higher OFI values indicate increasingly open propagation conditions with sparse multipath structure.

This regime classification extends beyond LOS/NLOS labeling by explicitly capturing the absence of dense scattering and obstruction mechanisms, enabling environment-aware channel characterization and complexity-adaptive network planning within the structured digital twin framework.

High multipath regions are characterized by elevated scattering intensity and dense interaction structures between transmitter and receiver. Such conditions are typically associated with increased fading susceptibility, broader delay spread, and reduced coherence bandwidth. Using the ray-tracing metadata available in the dataset, a High Multipath Index (HMI) can be defined as:

(A28) $$HMI=\frac{N_{int}}{N_{rays}},$$

where $N_{int}$ denotes the total number of interactions (reflections and diffractions) and $N_{rays}$ represents the number of launched rays. To account for spatial separation, a distance-normalized formulation may also be defined as:

(A29) $$HMI_{norm}=\frac{N_{int}}{N_{rays}\cdot d_{tx,rx}},$$

where $d_{tx,rx}$ is the transmitter–receiver separation distance.

Higher HMI values indicate increased multipath richness and scattering density, typically associated with enhanced small-scale fading and elevated dispersion risk. Classification into low-, moderate-, and high-multipath regimes may be performed using percentile-based or empirically selected thresholds. This regime identification extends beyond simple LOS/NLOS labeling and enables dispersion-aware and reliability-oriented network planning within the structured digital twin framework.

Shadowed regions correspond to propagation scenarios in which strong obstruction results in significant attenuation and reduced link reliability. Such conditions are typically associated with diffraction-dominated propagation, elevated excess path loss, and degraded link margin. A shadowing condition can be approximated using interaction and power-domain metrics derived from the dataset.

Let PL denote the path loss and $PL_{FS}$ the free-space path loss. The excess attenuation due to environmental obstruction is defined as:

(A30) $$PL_{excess}=PL-PL_{FS}.$$

Additionally, diffraction dominance may be quantified as:

(A31) $$D_{ratio}=\frac{N_{diff}}{N_{int}},$$

where $N_{diff}$ denotes total diffraction events and $N_{int}$ represents total interactions. A link may be classified as shadowed if it exhibits elevated excess attenuation and reduced link margin:

(A32) $$LM=P_{rx}-P_{sens}\leq \tau_{LM},$$

where $\tau_{LM}$ is a predefined reliability threshold.

This classification isolates deep obstruction scenarios beyond simple LOS/NLOS labeling and enables blockage-aware reliability assessment, outage risk identification, and environment-sensitive network planning within the structured digital twin framework.

#### Appendix E.2.3. Coverage Status Classification

Coverage Status Classification determines whether a receiver location satisfies minimum communication reliability requirements. Using the received signal strength $P_{rx}$ and receiver sensitivity $P_{sens}$ available in the dataset, link margin is defined as:

(A33) $$LM=P_{rx}-P_{sens}.$$

A receiver is classified as covered if:

(A34) LM>0,

and uncovered otherwise.

For finer-grained reliability assessment, multi-level coverage classes may be defined as:

- Outage: LM≤0;
- Marginal coverage: 0<LM≤τ;
- Robust coverage: LM>τ.

Here, τ is a predefined safety margin (e.g., 10 dB).

Since both normal and rain-affected RSSI values are available in the dataset, coverage status can also be evaluated under adverse weather conditions, enabling rain-aware reliability assessment and environment-sensitive network planning within the structured digital twin framework.

Reliable vs. Unreliable Link Classification. Beyond binary coverage status, practical network planning requires distinguishing links that are merely operational from those that provide sufficient robustness against fading and environmental variability. Using the received power $P_{rx}$ and receiver sensitivity $P_{sens}$, link margin is defined as:

(A35) $$LM=P_{rx}-P_{sens}.$$

A link can be classified as reliable if it exceeds a predefined safety margin $\tau_{rel}$:

(A36) $$\mathrm{Reliable}\Leftrightarrow LM\geq \tau_{rel},$$

and unreliable otherwise (optionally treating LM≤0 as an outage class). Typical planning values for $\tau_{rel}$ are in the range of 5–10 dB depending on desired robustness.

Since the dataset also includes rain-affected RSSI values, a weather-aware reliability criterion may be defined using:

(A37) $$LM_{rain}=P_{rx,rain}-P_{sens},$$

allowing classification of links that remain reliable under adverse conditions. This reliability-oriented labeling provides a stronger planning signal than nominal coverage alone and supports robust AP placement, power configuration, and multi-band deployment analysis within the structured digital twin framework.

High-quality vs Low-quality Channel Classification. Channel quality extends beyond received power and encompasses reliability margin, obstruction severity, and multipath dispersion characteristics. A composite Channel Quality Score (CQS) may be defined by integrating power-domain and interaction-domain descriptors:

(A38) $$CQS=\alpha \cdot LM_{norm}-\beta \cdot NSS-\gamma \cdot CDP-\delta \cdot \Delta_{rain},$$

where $LM_{norm}$ denotes normalized link margin, NSS is the NLOS Severity Score, CDP represents the Channel Dispersion Proxy, and $\Delta_{rain}$ is the rain-induced RSSI degradation. The coefficients α, β, γ, and δ may be selected through normalization or empirical calibration.

Higher CQS values correspond to channels exhibiting strong reliability margin, low obstruction severity, limited dispersion risk, and minimal weather sensitivity. Classification into high-, moderate-, and low-quality channel regimes enables quality-aware network planning and complements power-only coverage assessment within the structured digital twin framework.

#### Appendix E.2.4. Beam Alignment/Directional Classification

Dominant Direction Sector Classification, Directional communication systems rely on accurate beam alignment to maximize received power and link reliability. Using the azimuth angle θ between transmitter and receiver (stored as azimuth_angle column in the dataset), the angular domain may be partitioned into *K* sectors:

(A39) $$S_{k}=\left[\frac{360(k-1)}{K},\frac{360k}{K}\right),\;k=1,\ldots ,K.$$

For each transmitter–receiver pair, a dominant sector label may be assigned based on received power:

(A40) $$S_{dom}=\arg\max_{k}\;P_{rx}\left(\theta_{k}\right),$$

or alternatively using link margin for reliability-aware beam selection:

(A41) $$S_{dom}=\arg\max_{k}\;LM_{k}.$$

This multi-class classification task enables beam alignment prediction and directional propagation intelligence within the digital twin framework, supporting sectorized access point deployment and advanced beam management strategies in Wi-Fi 7 and future directional wireless systems.

Beam Steering Region Selection. Beyond selecting a single dominant angular sector, practical beamforming systems require identification of angular regions that provide stable and reliable performance. Let θ denote the azimuth angle and consider candidate steering regions defined as:

(A42) $$R_{k}=\left[\theta_{k}-\Delta ,\theta_{k}+\Delta \right],$$

where $\theta_{k}$ is a candidate beam center and Δ represents half the beamwidth.

For each region, a Beam Steering Region Score (BSRS) may be defined as:

(A43) $$BSRS_{k}=\alpha \cdot {\bar{LM}}_{k}-\beta \cdot {\bar{NSS}}_{k}-\gamma \cdot {\bar{CDP}}_{k}-\delta \cdot {\bar{\Delta_{rain}}}_{k},$$

where ${\bar{LM}}_{k}$ denotes the average link margin within region $R_{k}$, NSS is the NLOS Severity Score, CDP is the Channel Dispersion Proxy, and $\Delta_{rain}$ represents rain-induced signal degradation.

The optimal steering region is selected as:

(A44) $$R_{opt}=\arg\max_{k}BSRS_{k}.$$

This classification enables reliability-aware and dispersion-aware beam management within the structured digital twin framework, supporting sectorized Wi-Fi 7 deployments and advanced directional planning.
